# Nonsense-Mediated Decay Targeted RNA (ntRNA): Proposal of a ntRNA–miRNA–lncRNA Triple Regulatory Network Usable as Biomarker of Prognostic Risk in Patients with Kidney Cancer

**DOI:** 10.3390/genes13091656

**Published:** 2022-09-15

**Authors:** Zhiyue Zhou, Fuyan Hu, Dan Huang, Qingjia Chi, Nelson L. S. Tang

**Affiliations:** 1 Department of Statistics, School of Science, Wuhan University of Technology, 122 Luoshi Road, Wuhan 430070, China; 2 Department of Biology, Southern University of Science and Technology, Shenzhen 518055, China; 3 Department of Engineering Structure and Mechanics, School of Science, Wuhan University of Technology, Wuhan 430070, China; 4 Department of Chemical Pathology and Li Ka Shing Institute of Health Sciences, Faculty of Medicine, The Chinese University of Hong Kong, Hong Kong SAR, China; 5 Functional Genomics and Biostatistical Computing Laboratory, CUHK Shenzhen Research Institute, Shenzhen 518000, China; 6 Hong Kong Branch of CAS Center for Excellence in Animal Evolution and Genetics, School of Biomedical Sciences, The Chinese University of Hong Kong, Hong Kong SAR, China

**Keywords:** kidney renal clear cell carcinoma, nonsense-mediated decay targeted RNA, ntRNA–miRNA–lncRNA triple regulatory network, prognostic model

## Abstract

The most prevalent subtype of renal cell carcinoma (RCC), kidney renal clear cell carcinoma (KIRC) may be associated with a poor prognosis in a high number of cases, with a stage-specific prognostic stratification currently in use. No reliable biomarkers have been utilized so far in clinical practice despite the efforts in biomarker research in the last years. Nonsense-mediated mRNA decay (NMD) is a critical safeguard against erroneous transcripts, particularly mRNA transcripts containing premature termination codons (called nonsense-mediated decay targeted RNA, ntRNA). In this study, we first characterized 296 differentially expressed ntRNAs that were independent of the corresponding gene, 261 differentially expressed miRNAs, and 4653 differentially expressed lncRNAs. Then, we constructed a hub ntRNA–miRNA–lncRNA triple regulatory network associated with the prognosis of KIRC. Moreover, the results of immune infiltration analysis indicated that this network may influence the changes of the tumor immune microenvironment. A prognostic model derived from the genes and immune cells associated with the network was developed to distinguish between high- and low-risk patients, which was a better prognostic than other models, constructed using different biomarkers. Additionally, correlation of methylation and ntRNAs in the network suggested that some ntRNAs were regulated by methylation, which is helpful to further study the causes of abnormal expression of ntRNAs. In conclusion, this study highlighted the possible clinical implications of ntRNA functions in KIRC, proposing potential significant biomarkers that could be utilized to define the prognosis and design personalized treatment plans in kidney cancer management in the next future.

## 1. Introduction

Kidney cancer is a potentially lethal disease. According to the International Agency for Research on Cancer’s release of global cancer statistics for 2020, there were 179,368 fatalities and 431,288 new cases of kidney cancer worldwide [1]. The most prevalent histological type of kidney cancer is clear cell carcinoma, present in about 75% of cases, with an estimated 20–30% of patients presenting at diagnosis with distant metastasis and 20–40% of cases developing metastatic spread after primary surgical treatment [2,3]. Different cancers can have different clinical behavior in different patients. Utilization of biomarkers can be a very promising strategy in this field. Epigenetic-based biomarkers, such as aberrant DNA methylations, deregulated expression of chromatin structure proteins, and miRNAs or lncRNAs could have a high impact on clinical practice in urology and urological oncology [4]. Investigating potent biomarkers for KIRC is thus important. Nonsense-mediated decay (NMD) is a mRNA surveillance mechanism that prevents erroneous transcripts, especially those with premature termination codons (PTCs) [5], which has been discovered to take part in a variety of biological processes, particularly gene regulation [6]. We refer to this group of mRNA transcripts with PTCs as nonsense-mediated decay targeted RNA (ntRNA for short). In the traditional view, these abnormal transcripts are produced by mistake, and they are quickly cleared by NMD inside the cell, so the majority of them are expressed at low rates in various tissues and cell types [7]. However, in a previous study, we found that, in some cases, ntRNAs can be highly expressed, sometimes even more abundant than the normal transcripts of the corresponding genes [8].

A cellular post-transcriptional process called alternative splicing (AS) of precursor mRNA broadens the variety of proteins [9], which may result in nonsense-mediated decay targeted RNA [10]. Specifically, when a PTC-containing exon is included, or a PTC-containing intron is retained, or an upstream open reading frame (ORF) is presented in a transcript, NMD can be activated [11]. AS coupled to NMD (AS-NMD) directly regulates at least 20% of alternatively spliced genes [12], which is closely related to cancer as a novel post-transcriptional mechanism. For example, HGF downregulates SRSF1 by AS-NMD to decrease the level of tumor suppressor KLF6 in hepatocellular carcinoma [13].

Additional biomarkers have been recently gaining attention. For example, microRNAs (miRNAs), an 18–25 nucleotide (nt) long type of short single-stranded noncoding RNA, can regulate gene degradation or translation [14]. miRNAs regulate 30% of human genes by interacting with targets through miRNA response elements (MREs) [15]. Long noncoding RNAs (lncRNAs) with lengths longer than 200 nucleotides cannot or only partially code for proteins [16]. In the last 10 years, a large number of research studies have proved their essential effects in human cancers [17,18,19,20]. According to the competitive endogenous RNA (ceRNA) theory put forth by Salmena et al., while in the cytoplasm, lncRNAs are capable of competing with mRNA for miRNA pairing through MREs and, therefore, indirectly regulate mRNA expression [21]. The ceRNA regulatory networks play vital roles in cancer development. Dong et al. found that GAS5 inhibits tumor growth in the progression of KIRC by modulating the miR-223/hZIP1 pathway [22]. According to Zhou et al., the MALAT1/miR-1271-5p/KIAA1324 axis regulates the invasion of follicular helper T cells, which is directly related to the poor prognosis of KIRC [23]. In brief, it is useful to construct mRNA–miRNA–lncRNA triple regulatory networks through the ceRNA theory for exploring cancer biomarkers.

In this study, we constructed a hub ntRNA–miRNA–lncRNA triple regulatory network related to KIRC. Furthermore, immune infiltration analysis was utilized to identify hub immune cells related to the hub triple regulatory network and patient prognosis. Finally, we constructed a prognostic model that could be utilized for the clinical management of the KIRC patients. The results of survival analysis indicated that our proposed model and network strongly correlate with the survival rates of patients with kidney cancer. Additionally, the relationship between ntRNAs and methylation was studied to better characterize the regulatory mechanisms of ntRNAs. Our study provides data that can be used for prognostic stratification and designing a novel biomarker-based approach for kidney cancer management.

## 2. Materials and Methods

### 2.1. Data Preparation and Processing

**Figure 1:**
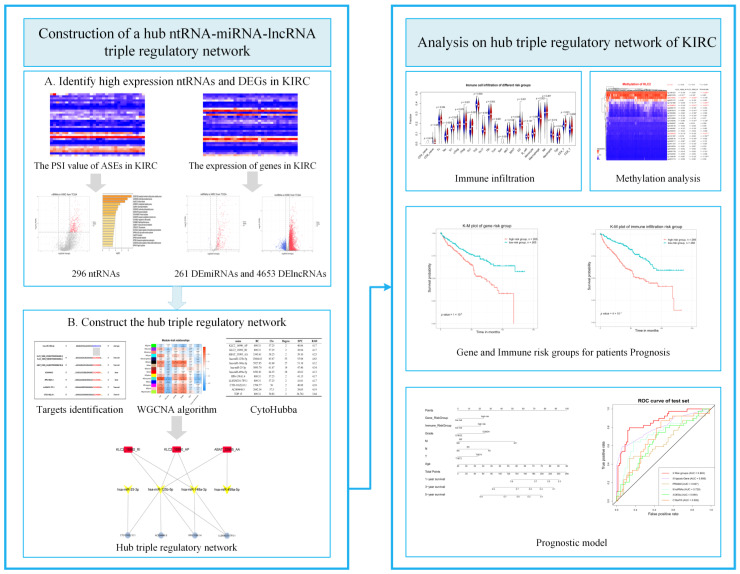
Research methodology of construction and analysis of a hub ntRNA–miRNA–lncRNA triple regulatory network in KIRC.

The gene expression of KIRC was downloaded from The Cancer Genome Atlas (TCGA, http://cancergenome.nih.gov/ (accessed on 8 March 2021)). According to the rules for the sample type code of TCGA, “01” means “primary solid tumor”, which was represented as the tumor sample, and “11” indicates “solid tissue normal”, which was represented as the normal sample in this study just like other studies [24,25]. For example, TCGA_A3_3358_01 means the tumor sample, and TCGA_A3_3358_11 indicates the corresponding normal sample. Genes that did not express in more than 60% of the samples were eliminated. TCGA SpliceSeq portal (https://bioinformatics.mdanderson.org/TCGASpliceSeq (accessed on 8 July 2021)) provided information on the AS events (ASEs) of KIRC, including each splicing event’s percent spliced-in (PSI) value in each sample. The PSI value measures the expression of splicing events using a ratio. Each ASE was given a name that included the gene symbol, ID number, and splicing type, such as KLC2_16990_AP. TCGA Data Commons (https://gdc.cancer.gov/ (accessed on 25 July 2021)) was used to gather clinical data. In addition, 24 immune cell abundance ratios were obtained from the Immune Cell Abundance Identifier [26] (ImmuCellAI, http://bioinfo.life.hust.edu.cn/ImmuCellAI#!/ (accessed on 18 October 2021)). Figure 1 shows a flowchart that explains the specifics of this study’s design.

### 2.2. Definition and Identification of the Differentially Expressed ntRNAs That Are Independent of Genes and Detection of Differentially Expressed Genes (DEGs)

Based on the two versions of human genome annotation files that were downloaded from the NCBI (GRCh37.p13) and Ensembl (GRCh37.73), we defined transcripts as the ntRNAs if they met one of the following criteria: for NCBI transcripts, the one with the prefix “NR_” was considered as ntRNA; the one with the prefix “NM_” was considered as a normal transcript, which will produce protein. For Ensembl transcripts, one of three criteria must be met before the transcript was defined as a ntRNA: (1) It is labeled with “not protein coding” by Ensembl; (2) it is labeled with “protein coding” by Ensembl, but it does not have a CCDS number; or (3) it is labeled with “protein coding” by Ensembl, but its length is less than 75% of the longest NM.

**Figure 2:**
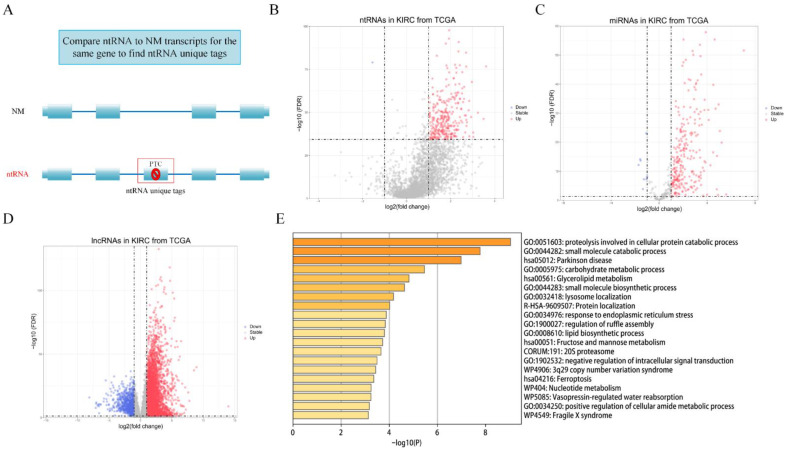
Selection of ntRNAs and DEGs. (**A**) Definition of ntRNA unique tags. (**B**–**D**) Volcano plots of 6720 ntRNAs, 377 miRNAs, and 8668 lncRNAs. (**E**) Top 20 significant enriched GO and pathways of 263 genes containing ntRNAs.

Due to AS, ntRNA may have ntRNA tags compared with NM (Figure 2A). After ntRNAs were defined, we compared them with all the NM transcripts of the gene to define the ntRNA tags that are unique in the ntRNA. Then, we searched for ntRNA unique tags in the ASE dataset. In subsequent experiments and analysis, ASEs corresponding to ntRNA unique tags were used as ntRNA.

Our study focused on the ntRNAs that were differentially expressed between tumor and normal samples, but the normal transcripts of the corresponding genes were not differentially expressed. We selected genes that were not differentially expressed using the 50th percentile of the false discovery rate (FDR) as the cutoff value. The differentially expressed ntRNAs were determined based on the 10th percentile of the FDR and |log2FC|>1 as criteria. Similarly, we determined the differentially expressed miRNAs (DEmiRNAs) and differentially expressed lncRNAs (DElncRNAs) with thresholds of FDR < 0.05 and and |log2FC|>1. Additionally, to investigate the potential biological pathways and mechanisms of ntRNAs, we performed a functional enrichment analysis of the genes containing ntRNAs in Metascape (https://metascape.org/gp/index.html#/main/step1 (accessed on 5 January 2022)) [27].

### 2.3. Establish a Triple Regulatory Network in KIRC

Following the theory that by competing with ntRNA for miRNA binding targets, lncRNA could indirectly regulate the expression of ntRNA [21], a triple regulatory network was created by performing the following procedures:

(1) TargetScan is a widely used database that searches for conserved 8mer, 7mer, and 6mer sites that match the seed region of each miRNA to anticipate biological targets for miRNAs [28]. In this paper, the targeted DEmiRNAs of the ntRNA were predicted using TargetScan8.0 (http://www.targetscan.org/vert_80/ (accessed on 20 September 2021)).

(2) The DIANA-microT technique is applied to predict miRNA targets on lncRNAs by bioinformatics software LncBase2 [29]. Millions of anticipated miRNA binding sites are provided by the pertinent module, together with comprehensive metadata and MRE conservation metrics. We used LncBase2 (http://carolina.imis.athenainnovation.gr/diana_tools/web/index.php?r=lncbasev2%2Findex-predicted (accessed on 25 September 2021)) to predict the DElncRNAs targeted by DEmiRNAs.

(3) ntRNA–miRNA and miRNA–lncRNA pairs are integrated to create a ntRNA–miRNA–lncRNA triple regulatory network.

As determined by the expression profiles of the selected ntRNAs, DEmiRNAs, and DElncRNAs, the R package WGCNA (short for weighted gene correlation network analysis), a scientific methodology in biology that compares the gene association patterns between different samples, was employed to create the weighted gene coexpression network and examine the modules that are associated to the prognosis [30]. The module that significantly correlated with sample traits (patients’ overall survival and stage, for example) was used for core gene selection. Using the Cytoscape (https://cytoscape.org (accessed on 10 October 2021)) plug-in cytoHubba [31], the hub triple regulatory network was then further identified. Only genes that ranked in the top 15 for all five methods, namely BC (Betweenness), Clo (Closeness), Degree, EPC, and RAD (Radiality) [32], were considered core genes.

### 2.4. Establishment of the Risk Prediction Model for the Hub Triple Regulatory Network

We combined the patient survival data for core genes with their expression, and then all samples were randomly split into training and test groups in a ratio 4:1. Following this, we used LASSO Cox regression to select the most appropriate variables from the hub triple regulatory network and finally obtained the risk score calculation formula:

(1) $$Riskscore_{gene}=\overset{n}{\underset{i=1}{\sum }}Coefficient\;of\;Gene_{i\;}\times Expression\;of\;Gene_{i\;}$$

where *Expression of Gene_i_* denotes the patient’s *i*th gene expression value, and *Coefficient of Gene_i_* denotes *Gene_i_*’s regression coefficient in the LASSO Cox regression model. Using the median risk score as the cutoff value, the samples were then split into high- and low-*Riskscore_gene_* groups. To compare the survival rates of high- and low-*Riskscore_gene_* groups, the Kaplan–Meier (KM) survival curve was plotted.

### 2.5. Establishment of Risk Prediction Model for the Immune Cells Associated with the Network

Kaplan–Meier survival curves and the log-rank test were performed to screen immune cells associated with the KIRC patients’ overall survival. Furthermore, the differences between high- and low- Riskscore*_gene_* groups in each immune cell were examined using the Wilcoxon rank-sum test. Finally, we screened out immune cells that were associated with both the hub triple regulatory network and overall survival and constructed a LASSO Cox regression model to quantitatively describe the association between immune cells and patients’ prognosis:

(2) $$Riskscore_{immune}=\overset{n}{\underset{i=1}{\sum }}Coefficient\;of\;immune\;cell_{i\;}\times Expression\;of\;immune\;cell_{i\;}$$

where *Expression of immune cell_i_* denotes the patient’s *i*th immune cell expression value, and *Coefficient of immune cell_i_* denotes *immune cell_i_*’s regression coefficient in the LASSO Cox regression model. Using the median risk score as the cutoff value, the samples were then split into high- and low-*Riskscore_immune_* groups. To compare the survival rates of high- and low-*Riskscore_immune_* groups, the Kaplan–Meier (KM) survival curve was plotted.

### 2.6. Using Core Gene and Immune Groups to Construct and Verify Cox Model

Multivariate Cox regression analysis was conducted including two risk groups (both gene and immune) and clinical information (grade, age, and TMN). Cox regression prognostic factors were then incorporated into a calibration chart and nomogram design using the R computer package “rms”, which would facilitate the application of our model to the clinical prognosis. To further assess the regression model’s capacity to forecast patient survival, the C-index of the regression analysis model was established. The consistency between the anticipated survival and observed survival was examined using calibration curves. Finally, five other KIRC studies in the last 3 years were replicated on the same dataset, and the results were compared using receiver operating characteristic (ROC) curves [33,34,35,36,37].

### 2.7. Methylation Analysis of ntRNAs in Hub Triple Regulatory Network

DNA methylation affects the behavior of cancer cells through three DNA methyltransferases (DNMT1, DNMT3A, and DNMT3B). The Wilcoxon rank-sum test was conducted to examine the significant difference of the expression of each DNA methyltransferases between the high- and low-ntRNA groups. After methylation probe data were downloaded from UCSC Xena (https://xenabrowser.net/datapages/ (accessed on 15 November 2021)), the Pearson correlation coefficient was used to analyze the relationship between the methylation probe and ntRNAs, as well as the connections between the methylation probe and patient overall survival to find the key methylation sites.

## 3. Results

### 3.1. Summary of Datasets

A total of 60,484 genes including 20,501 mRNAs, 14,353 lncRNAs, and 4077 miRNAs were found in The Cancer Genome Atlas (TCGA) database for 533 KIRC samples and 72 normal samples (Tables S1 and S2). We excluded genes that were not expressed in at least 60% of the samples, leaving 27,146 genes, namely 18,101 mRNAs, 8668 lncRNAs, and 377 miRNAs, for the study. The spectrum of ASEs was composed of 46,415 ASEs and involved 10,600 genes.

There were 530 cases with both clinical and immune information, and the samples were proportionately split into the training and test groups (4:1) at random. Table 1 displays the features of the samples in the training and test groups. In addition, 341 samples had methylation probe information.

### 3.2. Identification of Differentially Expressed ntRNAs and DEGs

A total of 102,309 ntRNA unique tags involving 15,045 genes were identified by our Python script, and 6720 ntRNAs involving 4598 genes were identified by the criteria (see method Section 2.2). log2FC and FDR techniques were utilized to detect differentially expressed ntRNAs independent of the corresponding genes, DEmiRNAs, and DElncRNAs; and, in total, 296 ntRNAs involving 263 genes, 261 DEmiRNAs, and 4653 DElncRNAs were identified in KIRC samples. The list of ntRNAs is shown in Table S3. In addition, volcano plots were drawn to display the distribution of ntRNAs, DEmiRNAs, and DElncRNAs (Figure 2B–D).

We performed a functional enrichment analysis utilizing Metascape for 263 genes containing ntRNAs. They were highly enriched in proteolysis, which is involved in the cellular protein catabolic process, small molecule catabolic process, carbohydrate metabolic process, and so on (Figure 2E). Therefore, ntRNAs play a vital role in a number of crucial biological pathways and mechanisms related to cancer.

### 3.3. Construction of the Hub ntRNA–miRNA–lncRNA Triple Regulatory Network in KIRC

The TargetScan database was firstly used to identify potential DEmiRNAs targeting transcripts associated with ntRNAs. Forty-six ntRNA–miRNA interaction pairs were identified, including 18 DEmiRNAs and 20 ntRNAs. These 20 ntRNAs had significantly higher expression in KIRC samples compared with normal samples. Secondly, the LncBase2 database was used to predict 18 DEmiRNAs and targets of these DEmiRNAs. A total of 2759 miRNA–lncRNA interaction pairs were discovered, containing 18 DEmiRNAs and 1624 DElncRNAs. Finally, we constructed a ntRNA–miRNA–lncRNA triple regulatory network containing 20 ntRNAs, 18 DEmiRNAs, and 1624 DElncRNAs with 2805 interactions (Tables S4 and S5 and Figure S1).

**Figure 3:**
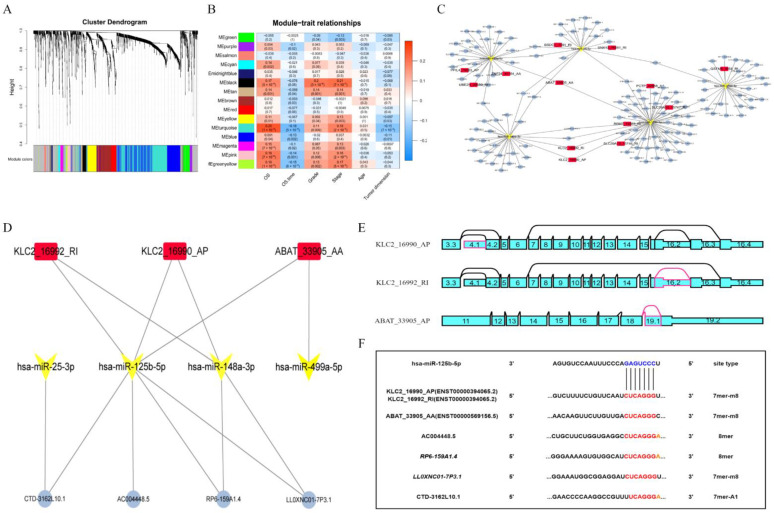
Establishment and analysis of the hub ntRNA–miRNA–lncRNA triple regulatory network. (**A**) Dynamic clustering analysis of genes. (**B**) Module–trait relationship correlation between genes in modules and clinical information. The table’s columns represent clinical data, and each row represents a gene module. (**C**) Triple regulatory network of turquoise modules. Red squares indicate ntRNAs, yellow V-shapes indicate DEmiRNAs, and blue circles indicate DElncRNAs. (**D**) Hub triple regulatory network. (**E**) Splice graphs of three ntRNAs in the hub triple regulatory network. Exons were drawn to scale, and connecting arcs represented splice paths. (**F**) Base pairing between hsa-miR-125b-5p and KLC2_16990_AP, KLC2_16992_RI, ABAT_33905_AP, AC004448.5, RP6-159A1.4, LL0XNC01-7P3.1, and CTD-3162L10.1 predicted by TargetScan and LncBase2.

A WGCNA algorithm was used to select the most critical genes. First, we chose the soft threshold power 5 using the approximation scale-free topology criterion (Figure 3A and Figure S2). Then, a total of 15 non-gray modules were found by setting the minimum number of module genes to 10 and merging the modules with a similarity lower than 0.85. Next, we determined the Pearson correlation coefficient (Cor) between each module and clinical data, and we discovered that the turquoise module was the most closely associated with a patient’s overall survival (OS) (Cor = 0.25, *p* = 1 × 10^−8^) and OS time (Cor = −0.25, *p* = 5 × 10^−5^). In addition, there was a significant positive connection between the stage and turquoise module (Cor = 0.18, *p* = 2 × 10^−5^) (Figure 3B). To sum up, we regarded the turquoise module as the most important module. Based on the turquoise module, a ntRNA–miRNA–lncRNA triple regulatory network including 13 ntRNAs, 5 DEmiRNAs, and 392 DElncRNAs was constructed (Figure 3C).

In addition, the hub triple regulatory network was further identified using the Cytoscape plug-in cytoHubba. By comparing the intersection of the top 15 scores for each of the five methods (see method Section 2.3) (Table S6), three ntRNAs (KLC2_16990_AP, KLC2_16992_RI, and ABAT_33905_AP), four DEmiRNAs (hsa-miR-25-3p, hsa-miR-125b-5p, hsa-miR-148a-3p, and hsa-miR-499a-5p), and four DElncRNAs (RP6-159A1.4, LL0XNC01-7P3.1, CTD-3162L10.1, and AC004448.5) were identified to establish a hub triple regulatory network (Figure 3D). The three ntRNA structures are shown in Figure 3E. The target sites of hsa-miR-125b-5p were predicted to pair with KLC2_16990_AP, KLC2_16992_RI, ABAT_33905_AP, AC004448.5, RP6-159A1.4, LL0XNC01-7P3.1, and CTD-3162L10.1 by TargetScan and LncBase2, and Figure 3F shows the binding sites between these genes.

### 3.4. Construction of a Prognostic Risk Score Model Based on the Hub Triple Regulatory Network

To prove that the genes in the hub triple regulatory network can be employed as possible biomarkers for the KIRC patients’ prognosis, we performed LASSO Cox regression on 11 genes in the hub triple regulatory network in the training set and selected six genes that were strongly related to the patient prognosis (Figure S3A). Finally, a risk score model was developed using the selected six genes to forecast the prognosis of the KIRC patients by the following formula:

(3) $$\begin{array}{cc} Riskscore_{gene} & =0.662\times \mathrm{KLC}2\text{\_}16990\text{\_}\mathrm{AP}+0.003\times \mathrm{hsa}-\mathrm{miR}-25-3p\\ & +0.196\times \mathrm{hsa}-\mathrm{miR}-148a-3p+0.166\times \mathrm{hsa}-\mathrm{miR}-499a-5p\\ & +0.023\times \mathrm{RP}6-159A1.4+0.027\times \mathrm{LL}0\mathrm{XNC}01-7P3.1 \end{array}$$

where the expression of each gene is indicated by the gene symbol, and the number before each gene symbol indicates the gene’s regression coefficient in the optimal LASSO Cox regression model. After determining each patient’s risk score using Formula (3), we divided the patients into high- and low-Riskscore*_gene_* groups with a cutoff value of the risk score’s median. A KM plot revealed significant differences in survival between the high- and low-Riskscore*_gene_* groups (Figure S4), confirming that the genes in the hub triple regulatory network we found are potential biomarkers for KIRC, which can be used to classify the prognostic risk of KIRC.

### 3.5. Correlation between Immune Infiltration and the Hub Triple Regulatory Network in KIRC

**Figure 4:**
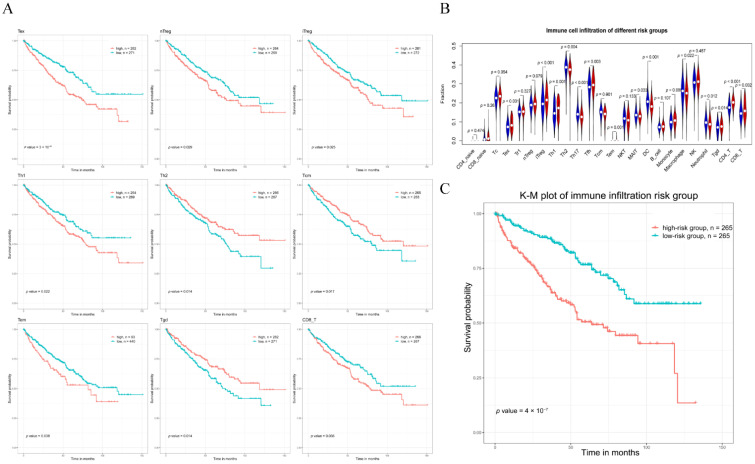
Analysis related to immune infiltration. (**A**) KM curves of immune cells (high-immune cell abundance vs. low-immune cell abundance) that significantly associated with OS. (**B**) Violin plot of 24 immune cells abundance between high-Riskscore*_gene_* group (red) and low-Riskscore*_gene_* one (blue), and *p* < 0.05 showed a significant difference between the two groups in immune cell expression. (**C**) KM plot (high-Riskscore*_immune_* vs. low-Riskscore*_immune_* groups) for KIRC patients.

Nine immune cells were significantly connected with the OS of the KIRC patients, according to the results of the survival analysis (Figure 4A). Then, we analyzed the immune infiltration levels between the high- and low-Riskscore*_gene_* groups by Wilcoxon rank-sum test, and 15 immune cells were differentially expressed under the standard *p* < 0.05 (Figure 4B). Finally, eight immune cells (i.e., Tex, iTreg, Th1, Tem, CD8_T, Th2, Tcm, and Tgd) were both associated with OS and differently expressed in between the high- and low-Riskscore*_gene_* groups.

Furthermore, LASSO Cox regression was performed on the eight immune cells to investigate the most significant immune cells for the prognosis. Ultimately, a risk model was created using four immune cells that were highly related with the prognosis (Figure S3B). Based on the immune infiltration of the four immune cells in the KIRC patients, we constructed the following formula to determine the risk scores:

(4) $$Riskscore_{immune}=5.992\times \mathrm{Tex}+6.977\times \mathrm{iTreg}+0.054\times \mathrm{Th}1\;-\;11.95\times \mathrm{Tcm}$$

where the expression of each immune cell is indicated by the immune cell symbol, and the number before each immune cell symbol indicates the immune cell’s regression coefficient in the optimal LASSO Cox regression model. After determining each patient’s risk score using Formula (4), we divided the patients into high- and low-Riskscore*_immune_* groups with a cutoff value of the risk score’s median. A KM plot revealed significant differences in survival between the high- and low-Riskscore*_immune_* groups (Figure 4C), confirming that the four immune cells can be used as a prognostic biomarker for KIRC.

### 3.6. Establishment and Validation of a Nomogram for OS Prediction in KIRC

**Figure 5:**
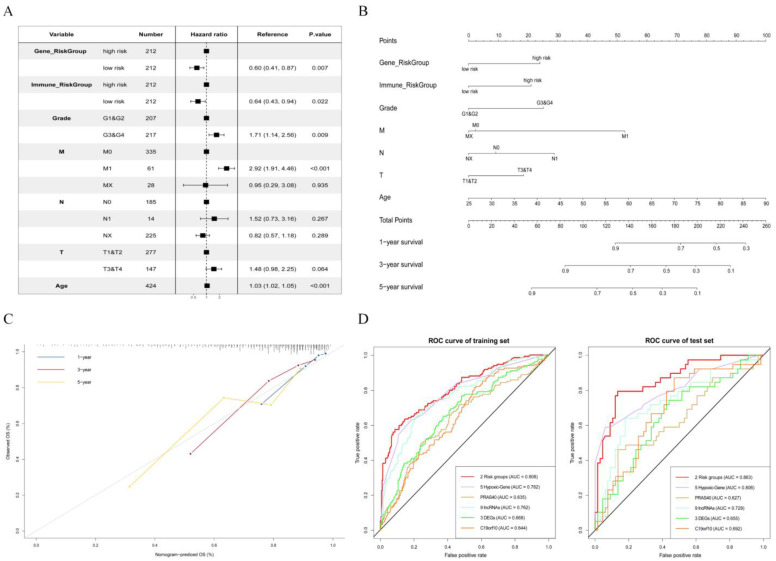
Nomogram for predicting overall survival (OS) in KIRC. (**A**) Multivariate Cox regression forest plot. (**B**) Nomogram for KIRC OS prediction. (**C**) Nomogram’s calibration plot for estimating OS rate at 1, 3, and 5 years. (**D**) ROC curve comparison with other studies in training set (*n* = 424) and test set (*n* = 106).

We performed a multivariate Cox regression that included our recorded risk groups (both Riskscore*_gene_* and Riskscore*_immune_*) and clinical information (grade, age, and TMN) from the samples in the training set. In the multivariate Cox model, Riskscore*_gene_* and Riskscore*_immune_* were associated with survival, proving that these two factors can be used as prognostic biomarkers of KIRC (Figure 5A). Then, a prognostic nomogram was developed to predict the survival rates for 1, 3, and 5 years of the KIRC patients according to the Cox model (Figure 5B), which may provide a new clinical prognostic model reference for doctors. For 1-, 3-, and 5-year OS rates, the calibration curve of this nomogram showed a fair agreement between the forecast and actual observations (Figure 5C), indicating that the generated nomogram had high accuracy for predicting OS in KIRC.

To further demonstrate the effectiveness of our multivariate Cox prognostic model, we compared our result with those of five other researchers’ works on the same training and test sets (Table 2 and Figure 5D). Notably, our prognostic model had the highest area under the curve (AUC) for both the training set (AUC = 80.8%) and the test set (AUC = 86.3%) as compared with other prognostic models, including the models with a single mRNA [34,37], several DEGs [36], and a class of genes [33,35], which demonstrated the excellent predictive OS ability of our prognostic model; moreover, the novel findings provide biomarkers with a potential value for prognostic stratification of patients and may improve personalized approaches to treatment.

### 3.7. DNA Methylation Involved in Regulating the Expression of KLC2_16990_AP and KLC2_16992_RI

**Figure 6:**
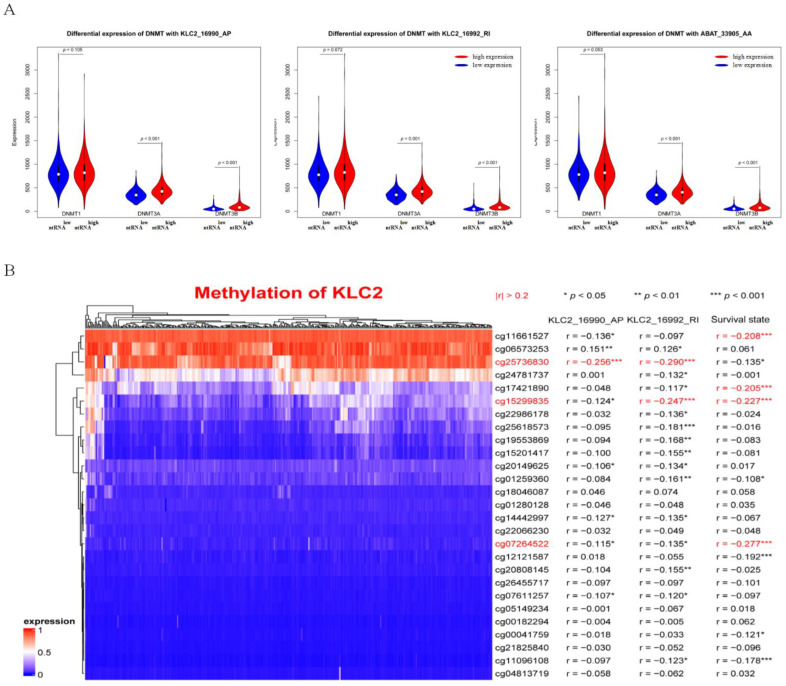
Analysis of ntRNA methylation. (**A**) Differential expression of three DNA methyltransferases (DNMT1, DNMT3A, and DNMT3B) with three ntRNAs (KLC2_16990_AP, KLC2_16992_RI, and ABAT_33905_AP). (**B**) Heatmap of methylation probes of KLC2.

We explored the correlation between the expression level of three ntRNAs (i.e., KLC2_16990_AP, KLC2_16992_RI, and ABAT_33905_AP) contained in our hub triple regulatory network and the methylation status. First, a comparison of the expression of three DNA methyltransferases (DNMT1, DNMT3A, and DNMT3B) in groups of ntRNAs with high and low expression revealed that the expressions of DNMT1, DNMT3A, and DNMT3B were significantly higher in KLC2_16990_AP, KLC2_16992_RI, and ABAT_33905_AP high-expression groups than the low-expression groups (Figure 6A). Furthermore, three methylation sites (cg25736830, cg1529835, and cg07264522) in KLC2 DNA sequences were negatively correlated with two ntRNA expression levels and patients’ survival state (Figure 6B). The results suggested that some ntRNA expressions in KIRC may be dysregulated as a result of DNA methylation. The expression level of ABAT 33905 AP, however, did not significantly correlate with the methylation sites in ABAT (Table S7). The reason for the abnormal expression of ntRNAs in KIRC needs to be further studied.

## 4. Discussion

In this study, we constructed a hub ntRNA–miRNA–lncRNA triple regulatory network to investigate the role of ntRNAs in KIRC, which could provide new perspective for the application of ntRNA data in clinical practice in urology. First, an in silico analysis was used to create a hub ntRNA–miRNA–lncRNA triple regulatory network with three ntRNAs, four DEmiRNAs, and four DElncRNAs. Then we analyzed the relationship between the network with a patient’s prognosis and immune infiltration level through LASSO Cox regression, multivariate Cox regression, and correlation analysis. The results suggested that the genes in our hub triple regulatory network were independent prognostic factors of KIRC and significantly affected the immune infiltration expression. A prognostic nomogram was developed for a multivariate model that includes Riskscore*_gene_* and Riskscore*_immune_* groups and clinical information (grade, age, and TMN). The results demonstrated its ability to predict KIRC patients’ prognosis with better performance as compared with other models [33,34,35,36,37]. These proved that our study identified novel biomarkers that could improve the prognostic stratification for KIRC patients. Finally, the results of methylation analysis showed that methylation influenced the expression of two ntRNAs in the hub triple regulatory network, suggesting that some ntRNAs’ expression in KIRC may be dysregulated due to DNA methylation.

This study focused on the independently high expression of ntRNA to find some interesting results that cannot be found by analyzing gene expression. Many of the enriched terms of the 263 genes containing ntRNAs have important roles in cancer initiation and development (Figure S5). Proteolysis involved in cellular protein catabolic process (GO: 0051603) has been reported in conjunctival melanoma and acute promyelocytic leukemia [38,39]. In another paper exploring hepatocellular carcinoma, small molecule catabolic process (GO: 0044282) was identified as the first significantly enriched gene ontology terms of DEGs [40]. It has been proven that PBRM1, the second-most often mutated gene in KIRC, affects the functioning of the carbohydrate metabolic pathway (GO: 0005975), a crucial biological process [41]. The process has also been reported in prostate cancer, lung cancer, and pancreatic ductal adenocarcinoma [42,43,44]. The level of glycerol in control renal tissue samples was 2.2 times higher than that in RCC, suggesting that glycerolipid metabolism (hsa00561) may be a key pathway in RCC [45], which was also confirmed by Lucarelli, G. et al. [46]. A recently identified form of cell death called ferroptosis (hsa04216) is caused by a buildup of iron-dependent lipid peroxide [47], and it has been observed that inhibiting ferroptosis in KIRC was found to inhibit tumor growth [48].

We constructed a hub ntRNA–miRNA–lncRNA triple regulatory network that statistically correlates with KIRC prognosis. Many genes in the network have been previously reported to be linked to kidney cancer growth and clinical behavior. For example, miR-125b-5p is in charge of maintaining the smooth muscle phenotype of juxtaglomerular cells [49]. In addition, miR-125b-5p was found elevated in RCC tissues and regulated RCC cell motility, invasion, and apoptosis, demonstrating that miR-125b-5p played a crucial role in RCC [50]. The expression of miR-148b-3p is upregulated in RCC, and miR-148b-3p is related with the DNA damage response, cell cycle progression, and apoptosis [51]. Meanwhile, miR-148b-3p directly binds to the 3′UTR of FOXK2 to inhibit FOXK2 from being expressed, and a poor prognosis is indicated by the low expression level of FOXK2 [52]. Similarly, the circPSD3/miR-25-3p/FBXW7 and miR-25-3p/IMPA2 axes increase KIRC cell invasion, motility, and the epithelial–mesenchymal transition (EMT) [53,54]. Heineman et al. noted that the expression value of miR-499a-5p was upregulated in RCC compared with benign renal tumors (BRTs) [55]. Interestingly, the gene ABAT acts as a tumor suppressor of KIRC [56], and ABAT_33905_AA expression in KIRC patients is significantly higher than that in normal samples, which deserves further study and exploration. Furthermore, based on the CTD (Comparative Toxicogenomics Database, https://ctdbase.org/ (accessed on 3 September 2022)) [57], we predicted 11 potential drugs, such as acetaminophen, doxorubicin, folic acid, celecoxib, and resveratrol, that can target five genes, namely ABAT, KLC2, hsa-miR-148a-3p, hsa-miR-25-3p, and hsa-miR-499a-5p, in KIRC, forming a gene–drug network (Figure S6). Furthermore, we extended the ntRNA–miRNA–lncRNA network to show the genes that can be targeted by drugs, which can more clearly demonstrate the relationship between drugs and the network (Figure S7). These drugs have been previously reported to be associated with KIRC. For instance, Cho et al. reported that taking acetaminophen raised the risk of significantly developing RCC [58]. In a xenograft model, doxorubicin combined with the TGase 2 inhibitor reduced the RCC growth [59]. Kabel et al. experimentally confirmed that administration of resveratrol can ameliorate KIRC [60]. Our study may offer helpful insight into the targeted treatment in KIRC. Our results provide advice for clinicians on drug choices in the treatment of KIRC patients.

Prior research has recognized the importance of immune infiltration for prognosis [61,62]. Moreover, it has recently been hypothesized that therapy, or combination of therapies, in cancer has to be adjusted to the tumor immunobiology; the potential use of immuno-based biomarkers, such as the ones we discovered, has been recently proposed in other urological tumors, such as bladder cancer [63]. In our study, eight of the 24 different types of immune cells that were associated with our hub triple regulatory network and overall survival of KIRC patients were explored. Vesicles with a diameter of 30 to 150 nm are known as tumor-derived exosomes (Texs). These vesicles are important mediums for communication between tumor immune cells [64], and Tex suppresses KIRC by inducing NK cell dysfunction [65]. The Th1/Th2 cytokines control the immune response to malignancy [66]. Patients with RCC showed a shift from Th1 to Th2 cells, indicating unbalanced Th1 and Th2 profiles [67]. CD8_T cells from normal donors recognized more antigens than those from patient sources and showed superior antitumor activity [68]. Therefore, these immune cells, which are linked to our hub triple regulatory network, have a significant impact on the development of KIRC.

To further explore the impact of our genes and immune cells on the prognosis of KIRC patients, we performed a multivariate Cox regression analysis. Our model included gene expression, patient characteristics, and immune infiltration at the same time, and the ROC curve of the model in the training set (AUC = 0.808) and test set (AUC = 0.863) achieves the best performance in comparison with other studies [33,34,35,36,37]. Moreover, few other models take into account the impact of gene expression, patient characteristics, and immune infection on patient prognosis at the same time, which may help to increase the model’s robustness.

The multiclass feature data improve the ability of our prognostic model to predict OS, but they also limit our selection of an external validation set. We failed to find any additional KIRC datasets that contain ntRNAs, gene expression, immune infection, and clinical data. To address this problem, we used the KIRP (kidney renal papillary cell carcinoma) data from TCGA as an external validation dataset. We substituted patient stage data for grade data (e.g., stage I is regarded as grade I, and stage II is regarded as grade II) since the KIRP dataset lacks patient-grade information. The AUC of our prediction models for KIRP was 0.703, the highest AUC among other prediction models [33,34,35,36,37] (Figure S8), which further demonstrated its ability to predict the prognosis of kidney cancer. There were probably two reasons why AUC in KIRP was lower than that in KIRC (AUC = 0.808): (1) we used stage instead of grade for verification, and (2) KIRP is still not a perfect external validation set for KIRC.

Our study has a number of limitations. First, additional experiments on the role of core genes are required. Second, the function and method by which lncRNA affects miRNA regulation on ntRNA need to be further elucidated.

## 5. Conclusions

This study focused on ntRNA, a novel potential useful biomarker for kidney cancer management, which was previously defined as the nonsense-mediated decay targeted RNA. The results of pathway enrichment analysis suggested that ntRNAs were strongly associated with the clinical evolution of KIRC. Therefore, we constructed a ntRNA–miRNA–lncRNA triple regulatory network that showed to be linked to the degree of immune infiltration and prognosis of KIRC. The prognostic model based on the Riskscore*_gene_* and Riskscore*_immune_* groups and clinical information achieved significant advantages, compared with other previous models, proving that our data can be utilized to design reliable prognostic biomarkers and promising therapeutical targets. Furthermore, we performed methylation analysis of ntRNAs in the network, which proved that some ntRNAs were regulated by methylation, providing a direction for future studies on the causes of the abnormal expression of ntRNAs.

## Figures and Tables

**Table 1:** Clinical characteristics of KIRC patients.

Characteristic	TCGA Training Set (*n* = 424)		TCGA Test Set (*n* = 106)	
	NO.	%	NO.	%
Sex				
Men	278	65.6	66	62.3
Women	146	34.4	40	37.7
Age, years				
Median	61	―	60	―
Range	26–90	―	36–90	―
T				
T1&T2	277	65.4	63	59.4
T3&T4	147	34.6	43	40.6
M				
M0	335	79.0	85	80.2
M1	61	14.4	17	16.0
MX	28	6.6	4	3.8
N				
N0	185	43.6	54	50.9
N1	14	3.3	2	1.9
NX	225	53.0	50	47.2
Grade				
G1&G2	197	46.5	42	39.6
G3&G4	217	53.5	64	60.4
Overall survival, years				
Median	3.51	―	3.69	―

**Table 2:** Classification results obtained by different prognostic model in KIRC dataset.

No.	Features	Prognostic Model Construction Methods	Accuracy (Training Set/Test Set)	Source
**1**	**2 risk groups**	**LASSO Cox and multivariate Cox regression**	**0.808/0.863**	**Our method**
2	5 Hypoxic-Genes	LASSO Cox and multivariate Cox regression	0.782/0.806	[33]
3	*PRAS40* (mRNA)	Univariate Cox regression	0.635/0.627	[34]
4	9 DERRlncRNAs	LASSO Cox regression	0.762/0.729	[35]
5	3 DEGs	LASSO Cox regression	0.668/0.665	[36]
6	*C19orf10* (mRNA)	Univariate Cox regression	0.644/0.692	[37]

## Data Availability

The results shown here are in whole or part based upon data generated by the TCGA Research Network of National Cancer Institute and National Human Genome Research Institute, Bethesda, MD, USA: https://www.cancer.gov/tcga (accessed on 5 September 2022). The TCGA data was downloaded from downloaded from the database of Genotypes and Phenotypes (dbGaP) at http://www.ncbi.nlm.nih.gov/gap (accessed on 5 September 2022) through dbGaP accession numbers phs000178: The Cancer Genome Atlas (TCGA).

## Supplementary Materials

The following supporting information can be downloaded at: https://www.mdpi.com/article/10.3390/genes13091656/s1, Figure S1: Triple regulatory network in KIRC. Red squares indicate HEntRNAs, yellow V-shapes indicate DEmiRNAs, and blue circles indicate DElncRNAs; Figure S2: Analysis of the scale-free index for various soft-threshold powers β in WGCNA; Figure S3: (A) Cross-validation plot for gene LASSO Cox model tuning parameter selection and profiles of LASSO coefficients for 11 genes in the network hub triple regulatory network. (B) Cross-validation plot for immune LASSO Cox model tuning parameter selection and profiles of LASSO coefficients for eight immune cells; Figure S4: KM plot (high-Riskscore*_gene_* vs. low-Riskscore*_gene_* groups) for KIRC patients; Figure S5: 55 enrichment Gene Ontology (GO) and pathways of 263 genes containing HEntRNA, including 33 GO biological processes, nine reactome pathways, seven KEGC pathways, five WikiPathways, and one CORUM pathway; Figure S6: Gene–drug network for genes in the hub triple regulatory network; Figure S7: Extended hub triple regulatory network with drug information; Figure S8: ROC curve comparison with other studies in KIRP dataset; Table S1: List of 533 KIRC samples; Table S2: List of 72 normal samples; Table S3: List of 296 HEntRNAs; Table S4: ntRNA–miRNA interaction pairs from TargetScan database; Table S5: miRNA–lncRNA interaction pairs from LncBase2 database; Table S6: Core genes obtained by cytoHubba’s five methods; Table S7: Correlation between ABAT methylation probes and ABAT_33905_AP expression in KIRC.
