# Supplementary material for: Nonsense-Mediated Decay Targeted RNA (ntRNA): Proposal of a ntRNA–miRNA–lncRNA Triple Regulatory Network Usable as Biomarker of Prognostic Risk in Patients with Kidney Cancer
Source: Genes (Basel). 2022 Sep 15;13(9):1656. doi: 10.3390/genes13091656 (PMC9498815; doi:10.3390/genes13091656)
Supplement: Supplementary file 1 [file genes-13-01656-s001.zip › Supplementary Figures.pdf]

Supplementary Figures

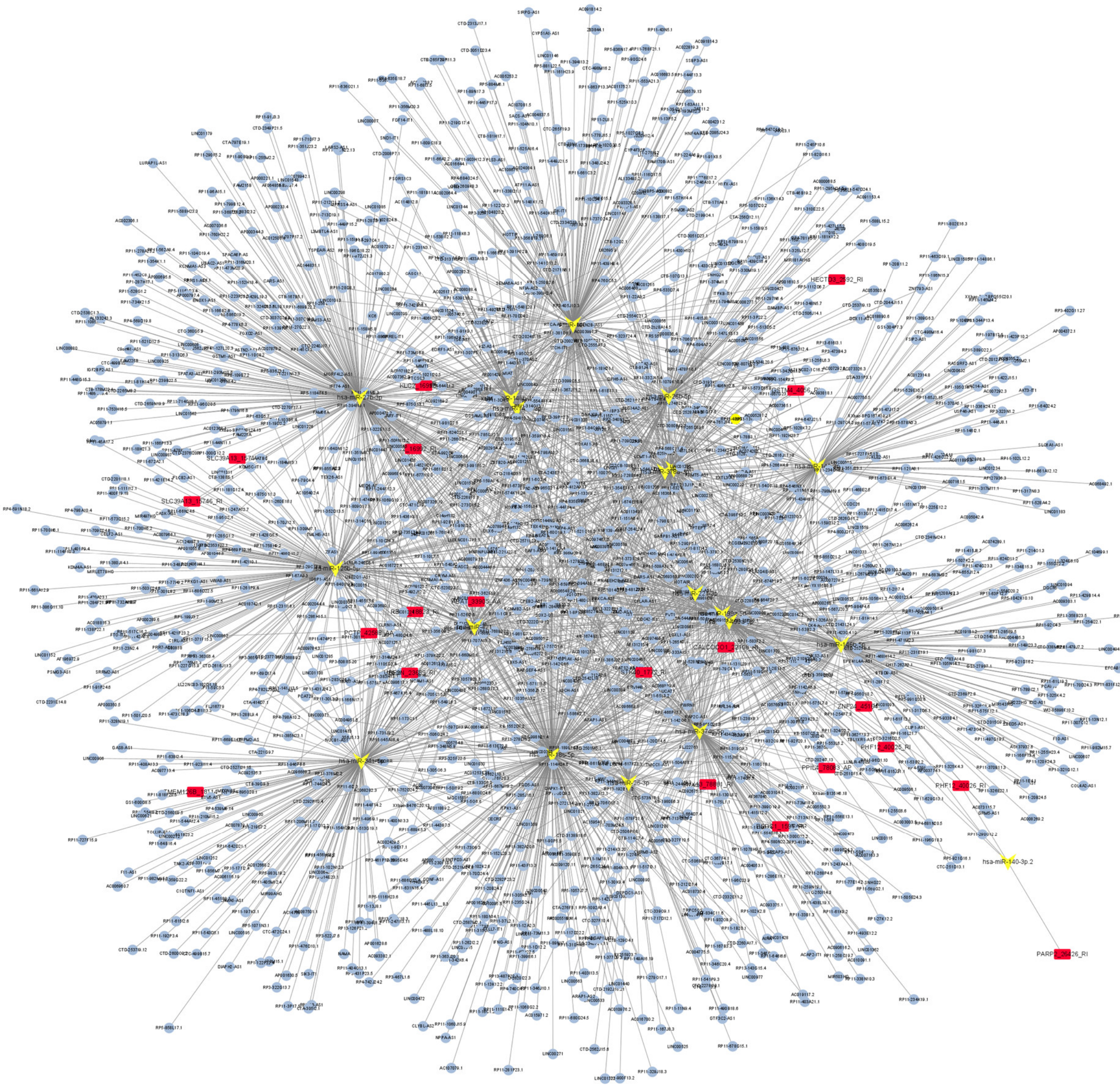

Figure S1. The triple regulatory network in KIRC. Red squares indicate HEntRNAs, yellow V-shapes indicate DEmiRNAs, and blue circles indicate DElncRNAs.

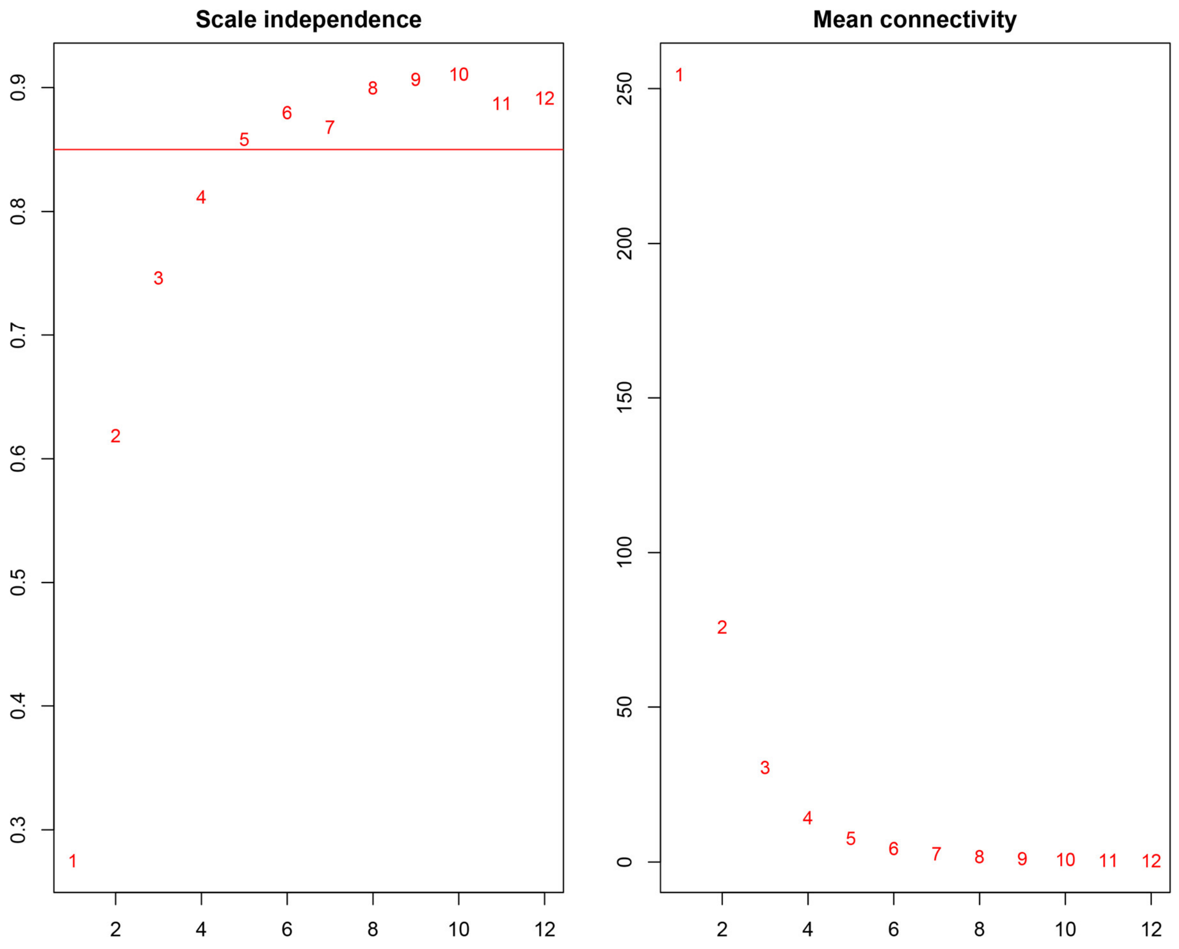

Figure S2. Analysis of the scale-free index R^2 for various soft-threshold powers β in WGCNA.

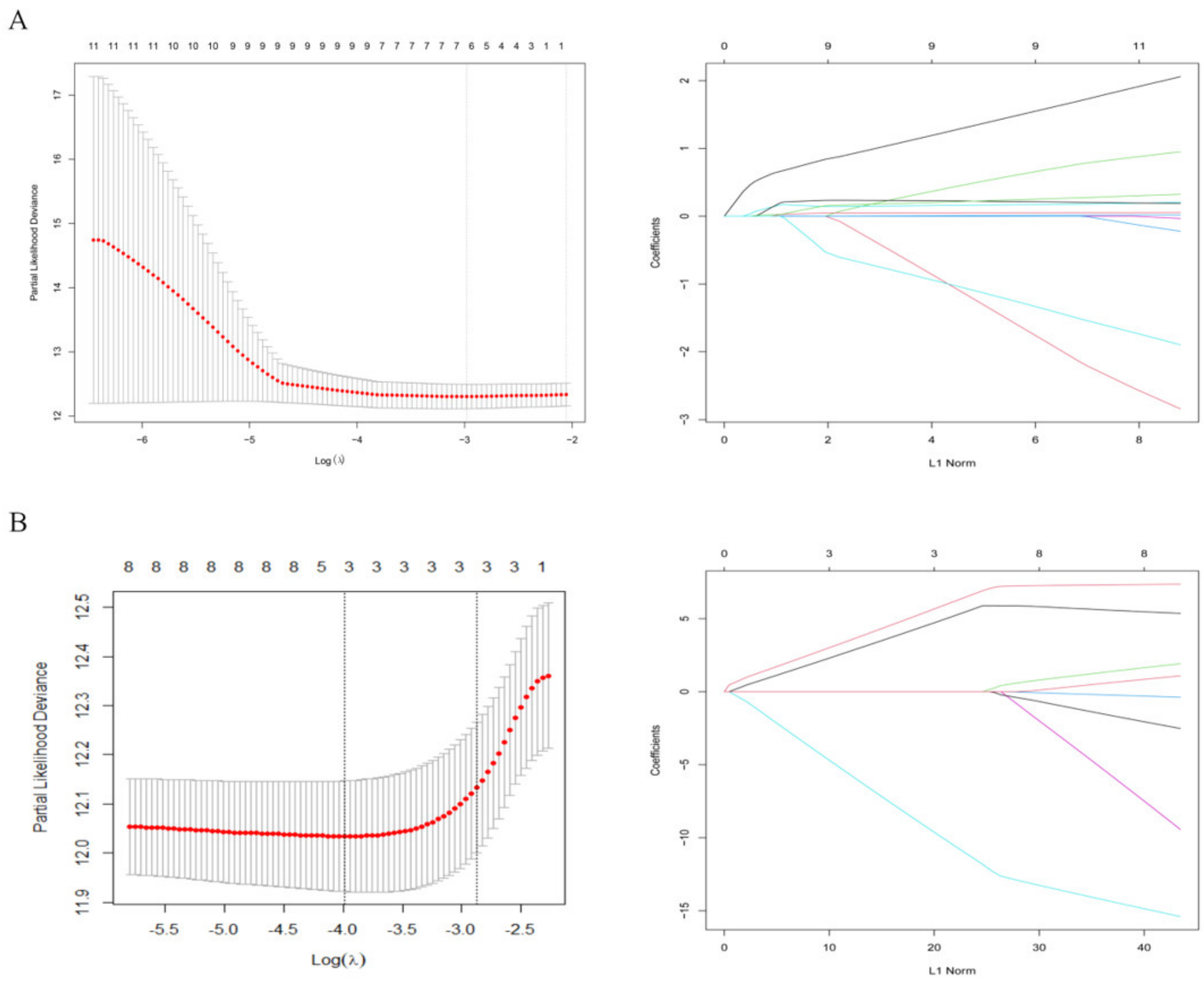

Figure S3. (A) Cross validation plot for the gene LASSO COX model tuning parameter selection and profiles of the LASSO coefficients for 11 genes in the network hub triple regulatory network. (B) Cross validation plot for the immune LASSO COX model tuning parameter selection and profiles of the LASSO coefficients for eight immune cells.

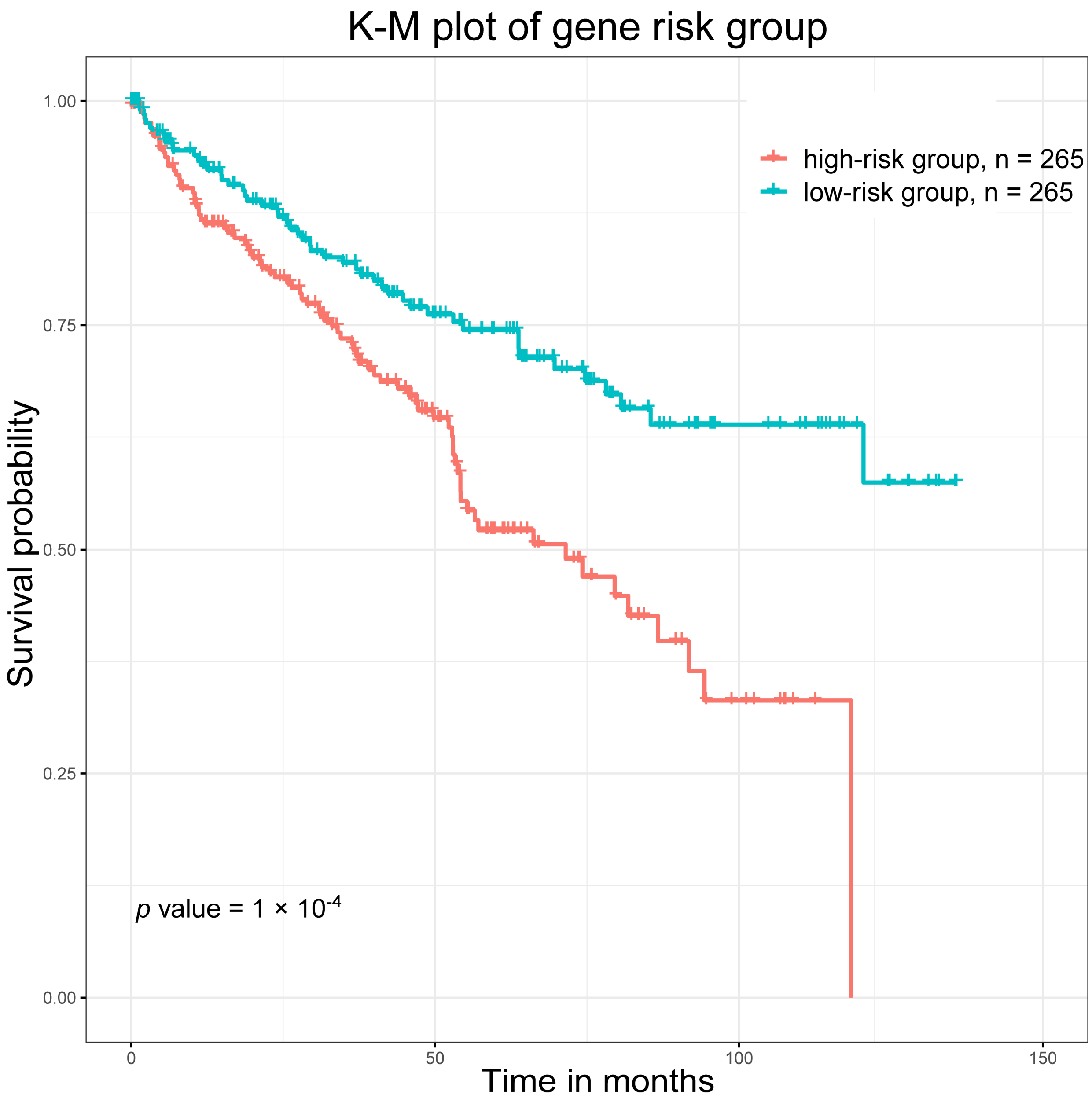

Figure S4. The KM plot (high- $Riskscore_{gene}$ vs. low- $Riskscore_{gene}$ group) for KIRC patients.

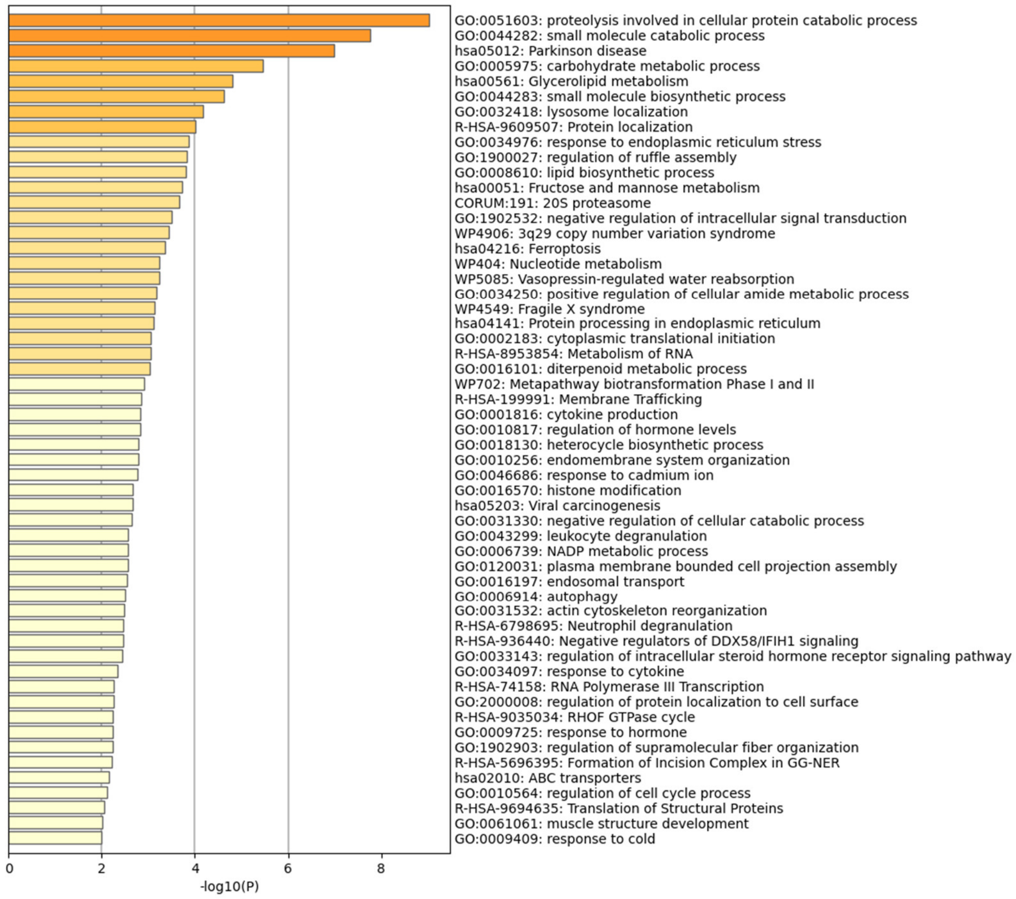

Figure S5. 55 enrichment Gene Ontology (GO) and pathways of 263 genes containing HEntRNA, including 33 GO biological processes, nine Reactome pathways, seven KEGC pathways, five WikiPathways and one CORUM pathways.

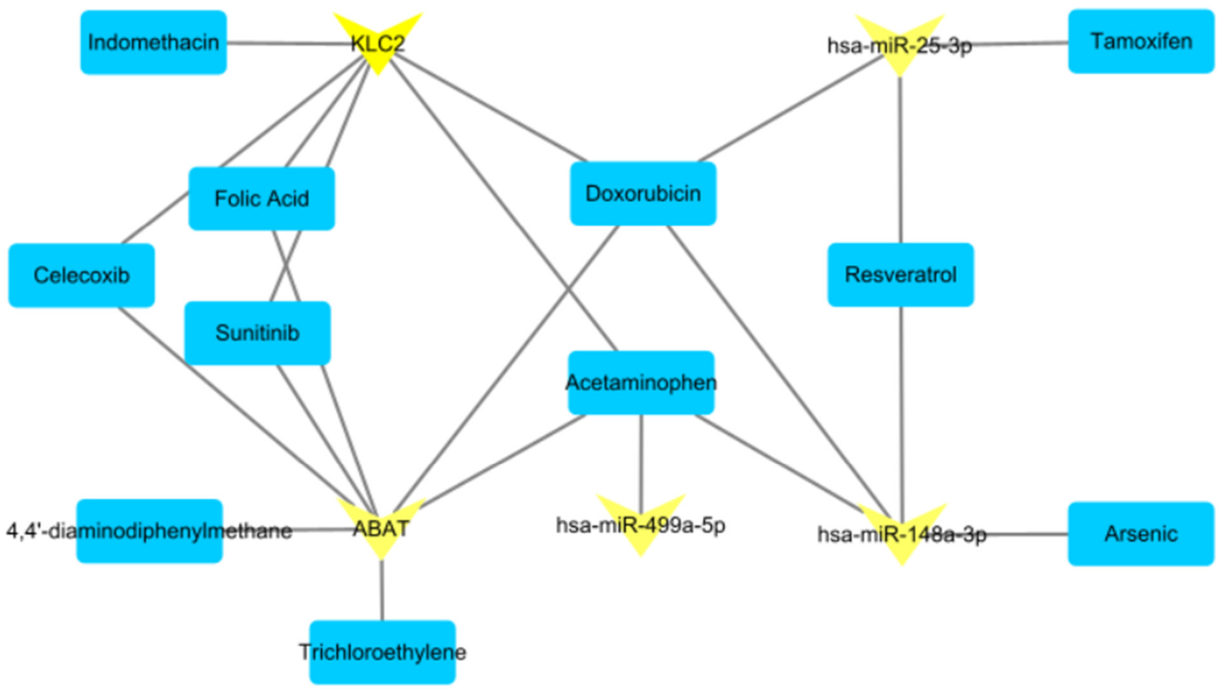

Figure S6. Gene-drug network for the genes in the hub triple regulatory network.

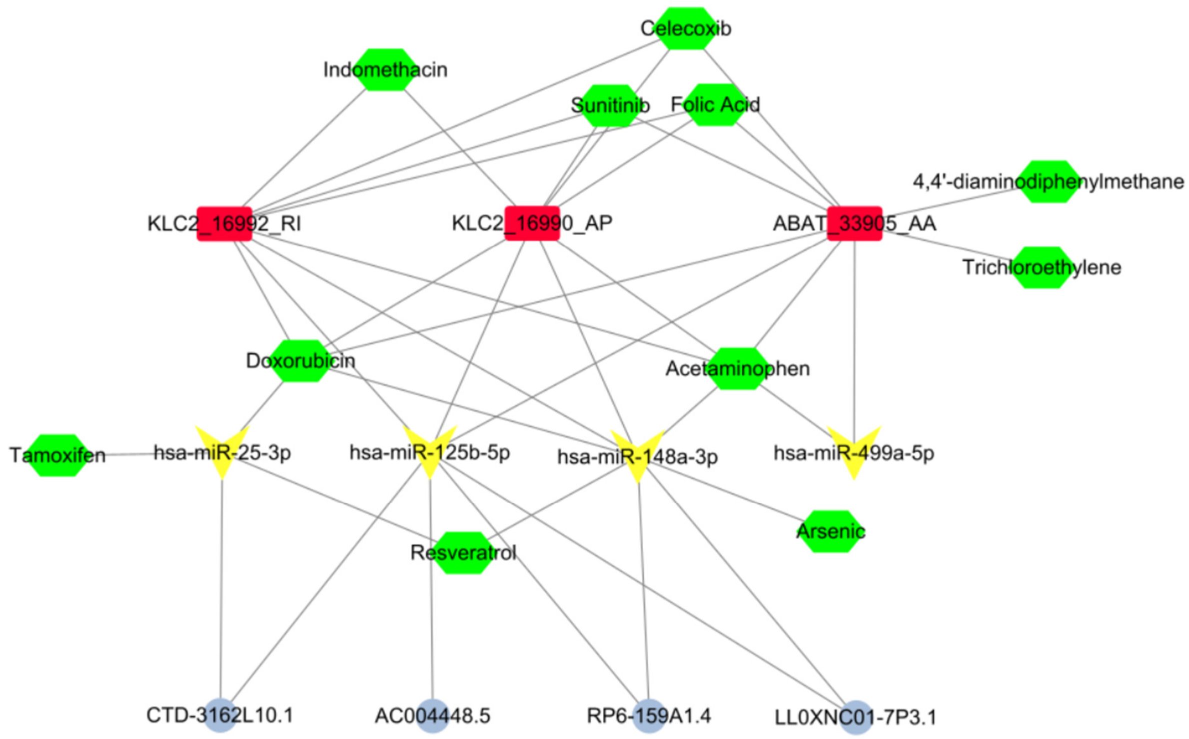

Figure S7. The extended hub triple regulatory network with drug information.

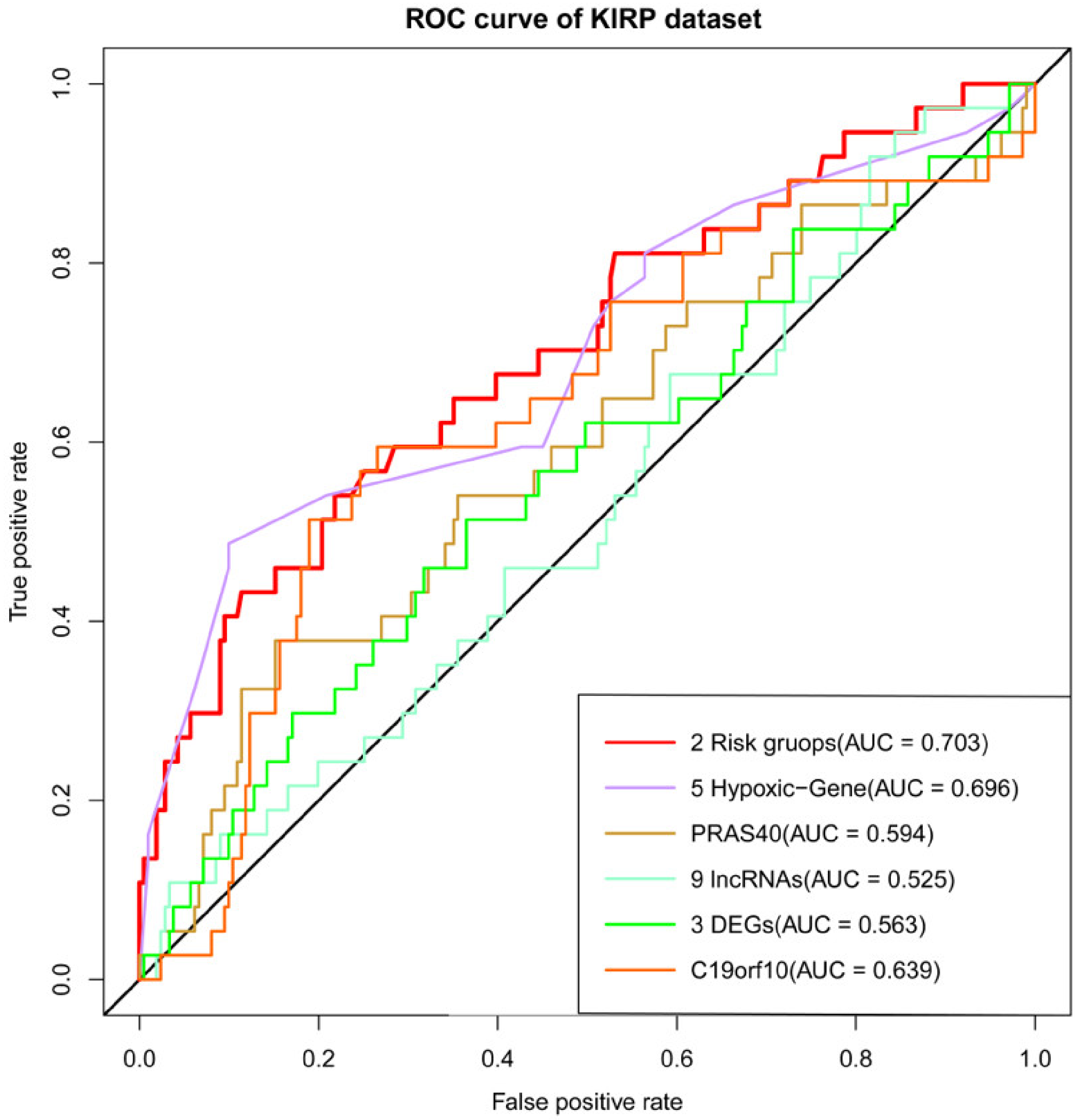

Figure S8. ROC curve comparison with other studies in the KIRP dataset.
