# Supplementary material for: Nonsense-Mediated Decay Targeted RNA (ntRNA): Proposal of a ntRNA–miRNA–lncRNA Triple Regulatory Network Usable as Biomarker of Prognostic Risk in Patients with Kidney Cancer
Source: Genes (Basel). 2022 Sep 15;13(9):1656. doi: 10.3390/genes13091656 (PMC9498815; doi:10.3390/genes13091656)
Supplement: Supplementary file 1 [file genes-13-01656-s001.zip › Supplementary Tables.pdf]

# Supplementary Tables

**Table S1.** List of 533 KIRC samples.

| TCGA_3Z_A93Z_01 | TCGA_B0_4821_01 | TCGA_B8_4620_01 | TCGA_BP_4969_01 | TCGA_CJ_4916_01 |
|---|---|---|---|---|
| TCGA_6D_AA2E_01 | TCGA_B0_4822_01 | TCGA_B8_4621_01 | TCGA_BP_4970_01 | TCGA_CJ_4918_01 |
| TCGA_A3_3306_01 | TCGA_B0_4823_01 | TCGA_B8_4622_01 | TCGA_BP_4971_01 | TCGA_CJ_4920_01 |
| TCGA_A3_3307_01 | TCGA_B0_4824_01 | TCGA_B8_5158_01 | TCGA_BP_4972_01 | TCGA_CJ_4923_01 |
| TCGA_A3_3308_01 | TCGA_B0_4827_01 | TCGA_B8_5159_01 | TCGA_BP_4973_01 | TCGA_CJ_5671_01 |
| TCGA_A3_3311_01 | TCGA_B0_4828_01 | TCGA_B8_5162_01 | TCGA_BP_4974_01 | TCGA_CJ_5672_01 |
| TCGA_A3_3313_01 | TCGA_B0_4833_01 | TCGA_B8_5163_01 | TCGA_BP_4975_01 | TCGA_CJ_5675_01 |
| TCGA_A3_3316_01 | TCGA_B0_4834_01 | TCGA_B8_5164_01 | TCGA_BP_4976_01 | TCGA_CJ_5676_01 |
| TCGA_A3_3317_01 | TCGA_B0_4836_01 | TCGA_B8_5165_01 | TCGA_BP_4977_01 | TCGA_CJ_5677_01 |
| TCGA_A3_3319_01 | TCGA_B0_4837_01 | TCGA_B8_5545_01 | TCGA_BP_4981_01 | TCGA_CJ_5678_01 |
| TCGA_A3_3320_01 | TCGA_B0_4838_01 | TCGA_B8_5546_01 | TCGA_BP_4982_01 | TCGA_CJ_5679_01 |
| TCGA_A3_3322_01 | TCGA_B0_4839_01 | TCGA_B8_5549_01 | TCGA_BP_4983_01 | TCGA_CJ_5680_01 |
| TCGA_A3_3323_01 | TCGA_B0_4841_01 | TCGA_B8_5550_01 | TCGA_BP_4985_01 | TCGA_CJ_5681_01 |
| TCGA_A3_3324_01 | TCGA_B0_4842_01 | TCGA_B8_5551_01 | TCGA_BP_4986_01 | TCGA_CJ_5682_01 |
| TCGA_A3_3325_01 | TCGA_B0_4843_01 | TCGA_B8_5552_01 | TCGA_BP_4987_01 | TCGA_CJ_5683_01 |
| TCGA_A3_3326_01 | TCGA_B0_4844_01 | TCGA_B8_5553_01 | TCGA_BP_4988_01 | TCGA_CJ_5684_01 |
| TCGA_A3_3328_01 | TCGA_B0_4845_01 | TCGA_B8_A54D_01 | TCGA_BP_4989_01 | TCGA_CJ_5686_01 |
| TCGA_A3_3329_01 | TCGA_B0_4846_01 | TCGA_B8_A54E_01 | TCGA_BP_4991_01 | TCGA_CJ_5689_01 |
| TCGA_A3_3331_01 | TCGA_B0_4847_01 | TCGA_B8_A54F_01 | TCGA_BP_4992_01 | TCGA_CJ_6027_01 |
| TCGA_A3_3335_01 | TCGA_B0_4848_01 | TCGA_B8_A54G_01 | TCGA_BP_4993_01 | TCGA_CJ_6028_01 |
| TCGA_A3_3343_01 | TCGA_B0_4849_01 | TCGA_B8_A54H_01 | TCGA_BP_4994_01 | TCGA_CJ_6030_01 |
| TCGA_A3_3346_01 | TCGA_B0_4852_01 | TCGA_B8_A54I_01 | TCGA_BP_4995_01 | TCGA_CJ_6031_01 |
| TCGA_A3_3347_01 | TCGA_B0_4945_01 | TCGA_B8_A54J_01 | TCGA_BP_4998_01 | TCGA_CJ_6032_01 |
| TCGA_A3_3349_01 | TCGA_B0_5075_01 | TCGA_B8_A54K_01 | TCGA_BP_4999_01 | TCGA_CJ_6033_01 |
| TCGA_A3_3351_01 | TCGA_B0_5077_01 | TCGA_B8_A7U6_01 | TCGA_BP_5000_01 | TCGA_CW_5580_01 |
| TCGA_A3_3352_01 | TCGA_B0_5080_01 | TCGA_B8_A8YJ_01 | TCGA_BP_5001_01 | TCGA_CW_5581_01 |
| TCGA_A3_3357_01 | TCGA_B0_5081_01 | TCGA_BP_4158_01 | TCGA_BP_5004_01 | TCGA_CW_5583_01 |
| TCGA_A3_3358_01 | TCGA_B0_5083_01 | TCGA_BP_4159_01 | TCGA_BP_5006_01 | TCGA_CW_5584_01 |
| TCGA_A3_3359_01 | TCGA_B0_5084_01 | TCGA_BP_4160_01 | TCGA_BP_5007_01 | TCGA_CW_5585_01 |
| TCGA_A3_3362_01 | TCGA_B0_5085_01 | TCGA_BP_4161_01 | TCGA_BP_5008_01 | TCGA_CW_5587_01 |
| TCGA_A3_3363_01 | TCGA_B0_5088_01 | TCGA_BP_4162_01 | TCGA_BP_5009_01 | TCGA_CW_5588_01 |
| TCGA_A3_3365_01 | TCGA_B0_5092_01 | TCGA_BP_4163_01 | TCGA_BP_5010_01 | TCGA_CW_5589_01 |
| TCGA_A3_3367_01 | TCGA_B0_5094_01 | TCGA_BP_4164_01 | TCGA_BP_5168_01 | TCGA_CW_5590_01 |
| TCGA_A3_3370_01 | TCGA_B0_5095_01 | TCGA_BP_4165_01 | TCGA_BP_5169_01 | TCGA_CW_5591_01 |
| TCGA_A3_3372_01 | TCGA_B0_5096_01 | TCGA_BP_4166_01 | TCGA_BP_5170_01 | TCGA_CW_6087_01 |
| TCGA_A3_3373_01 | TCGA_B0_5097_01 | TCGA_BP_4167_01 | TCGA_BP_5173_01 | TCGA_CW_6088_01 |
| TCGA_A3_3374_01 | TCGA_B0_5098_01 | TCGA_BP_4169_01 | TCGA_BP_5174_01 | TCGA_CW_6090_01 |
| TCGA_A3_3376_01 | TCGA_B0_5099_01 | TCGA_BP_4170_01 | TCGA_BP_5175_01 | TCGA_CW_6093_01 |
| TCGA_A3_3378_01 | TCGA_B0_5100_01 | TCGA_BP_4173_01 | TCGA_BP_5176_01 | TCGA_CW_6097_01 |

| | | | | |
|---|---|---|---|---|
| TCGA_A3_3380_01 | TCGA_B0_5102_01 | TCGA_BP_4174_01 | TCGA_BP_5177_01 | TCGA_CZ_4853_01 |
| TCGA_A3_3382_01 | TCGA_B0_5104_01 | TCGA_BP_4176_01 | TCGA_BP_5178_01 | TCGA_CZ_4854_01 |
| TCGA_A3_3383_01 | TCGA_B0_5106_01 | TCGA_BP_4177_01 | TCGA_BP_5180_01 | TCGA_CZ_4856_01 |
| TCGA_A3_3385_01 | TCGA_B0_5107_01 | TCGA_BP_4325_01 | TCGA_BP_5181_01 | TCGA_CZ_4857_01 |
| TCGA_A3_3387_01 | TCGA_B0_5108_01 | TCGA_BP_4326_01 | TCGA_BP_5182_01 | TCGA_CZ_4858_01 |
| TCGA_A3_A6NI_01 | TCGA_B0_5109_01 | TCGA_BP_4327_01 | TCGA_BP_5183_01 | TCGA_CZ_4859_01 |
| TCGA_A3_A6NJ_01 | TCGA_B0_5110_01 | TCGA_BP_4329_01 | TCGA_BP_5184_01 | TCGA_CZ_4860_01 |
| TCGA_A3_A6NL_01 | TCGA_B0_5113_01 | TCGA_BP_4330_01 | TCGA_BP_5185_01 | TCGA_CZ_4861_01 |
| TCGA_A3_A6NN_01 | TCGA_B0_5115_01 | TCGA_BP_4331_01 | TCGA_BP_5186_01 | TCGA_CZ_4862_01 |
| TCGA_A3_A8CQ_01 | TCGA_B0_5116_01 | TCGA_BP_4332_01 | TCGA_BP_5187_01 | TCGA_CZ_4863_01 |
| TCGA_A3_A8OU_01 | TCGA_B0_5117_01 | TCGA_BP_4334_01 | TCGA_BP_5189_01 | TCGA_CZ_4864_01 |
| TCGA_A3_A8OV_01 | TCGA_B0_5119_01 | TCGA_BP_4335_01 | TCGA_BP_5190_01 | TCGA_CZ_4865_01 |
| TCGA_A3_A8OW_01 | TCGA_B0_5120_01 | TCGA_BP_4337_01 | TCGA_BP_5191_01 | TCGA_CZ_4866_01 |
| TCGA_A3_A8OX_01 | TCGA_B0_5121_01 | TCGA_BP_4338_01 | TCGA_BP_5192_01 | TCGA_CZ_5451_01 |
| TCGA_AK_3425_01 | TCGA_B0_5399_01 | TCGA_BP_4340_01 | TCGA_BP_5194_01 | TCGA_CZ_5452_01 |
| TCGA_AK_3426_01 | TCGA_B0_5400_01 | TCGA_BP_4341_01 | TCGA_BP_5195_01 | TCGA_CZ_5453_01 |
| TCGA_AK_3427_01 | TCGA_B0_5402_01 | TCGA_BP_4342_01 | TCGA_BP_5196_01 | TCGA_CZ_5454_01 |
| TCGA_AK_3428_01 | TCGA_B0_5690_01 | TCGA_BP_4343_01 | TCGA_BP_5198_01 | TCGA_CZ_5455_01 |
| TCGA_AK_3429_01 | TCGA_B0_5691_01 | TCGA_BP_4344_01 | TCGA_BP_5199_01 | TCGA_CZ_5456_01 |
| TCGA_AK_3431_01 | TCGA_B0_5692_01 | TCGA_BP_4345_01 | TCGA_BP_5200_01 | TCGA_CZ_5457_01 |
| TCGA_AK_3433_01 | TCGA_B0_5693_01 | TCGA_BP_4346_01 | TCGA_BP_5201_01 | TCGA_CZ_5458_01 |
| TCGA_AK_3434_01 | TCGA_B0_5694_01 | TCGA_BP_4347_01 | TCGA_BP_5202_01 | TCGA_CZ_5459_01 |
| TCGA_AK_3436_01 | TCGA_B0_5695_01 | TCGA_BP_4349_01 | TCGA_CJ_4634_01 | TCGA_CZ_5460_01 |
| TCGA_AK_3440_01 | TCGA_B0_5696_01 | TCGA_BP_4351_01 | TCGA_CJ_4635_01 | TCGA_CZ_5461_01 |
| TCGA_AK_3443_01 | TCGA_B0_5697_01 | TCGA_BP_4352_01 | TCGA_CJ_4636_01 | TCGA_CZ_5462_01 |
| TCGA_AK_3444_01 | TCGA_B0_5698_01 | TCGA_BP_4353_01 | TCGA_CJ_4637_01 | TCGA_CZ_5463_01 |
| TCGA_AK_3445_01 | TCGA_B0_5699_01 | TCGA_BP_4354_01 | TCGA_CJ_4638_01 | TCGA_CZ_5464_01 |
| TCGA_AK_3447_01 | TCGA_B0_5700_01 | TCGA_BP_4355_01 | TCGA_CJ_4639_01 | TCGA_CZ_5465_01 |
| TCGA_AK_3450_01 | TCGA_B0_5701_01 | TCGA_BP_4756_01 | TCGA_CJ_4640_01 | TCGA_CZ_5466_01 |
| TCGA_AK_3451_01 | TCGA_B0_5702_01 | TCGA_BP_4758_01 | TCGA_CJ_4641_01 | TCGA_CZ_5467_01 |
| TCGA_AK_3453_01 | TCGA_B0_5703_01 | TCGA_BP_4759_01 | TCGA_CJ_4642_01 | TCGA_CZ_5468_01 |
| TCGA_AK_3454_01 | TCGA_B0_5705_01 | TCGA_BP_4760_01 | TCGA_CJ_4643_01 | TCGA_CZ_5469_01 |
| TCGA_AK_3455_01 | TCGA_B0_5706_01 | TCGA_BP_4761_01 | TCGA_CJ_4644_01 | TCGA_CZ_5470_01 |
| TCGA_AK_3456_01 | TCGA_B0_5707_01 | TCGA_BP_4762_01 | TCGA_CJ_4868_01 | TCGA_CZ_5982_01 |
| TCGA_AK_3458_01 | TCGA_B0_5709_01 | TCGA_BP_4763_01 | TCGA_CJ_4869_01 | TCGA_CZ_5984_01 |
| TCGA_AK_3460_01 | TCGA_B0_5710_01 | TCGA_BP_4765_01 | TCGA_CJ_4870_01 | TCGA_CZ_5985_01 |
| TCGA_AK_3461_01 | TCGA_B0_5711_01 | TCGA_BP_4766_01 | TCGA_CJ_4871_01 | TCGA_CZ_5986_01 |
| TCGA_AK_3465_01 | TCGA_B0_5712_01 | TCGA_BP_4768_01 | TCGA_CJ_4872_01 | TCGA_CZ_5987_01 |
| TCGA_AS_3777_01 | TCGA_B0_5713_01 | TCGA_BP_4769_01 | TCGA_CJ_4873_01 | TCGA_CZ_5988_01 |
| TCGA_AS_3778_01 | TCGA_B0_5812_01 | TCGA_BP_4770_01 | TCGA_CJ_4874_01 | TCGA_CZ_5989_01 |
| TCGA_B0_4688_01 | TCGA_B2_3923_01 | TCGA_BP_4771_01 | TCGA_CJ_4875_01 | TCGA_DV_5565_01 |
| TCGA_B0_4690_01 | TCGA_B2_3924_01 | TCGA_BP_4774_01 | TCGA_CJ_4876_01 | TCGA_DV_5566_01 |
| TCGA_B0_4691_01 | TCGA_B2_4098_01 | TCGA_BP_4775_01 | TCGA_CJ_4878_01 | TCGA_DV_5567_01 |

| | | | | |
|---|---|---|---|---|
| TCGA_B0_4693_01 | TCGA_B2_4099_01 | TCGA_BP_4776_01 | TCGA_CJ_4881_01 | TCGA_DV_5568_01 |
| TCGA_B0_4694_01 | TCGA_B2_4101_01 | TCGA_BP_4777_01 | TCGA_CJ_4882_01 | TCGA_DV_5569_01 |
| TCGA_B0_4696_01 | TCGA_B2_4102_01 | TCGA_BP_4781_01 | TCGA_CJ_4884_01 | TCGA_DV_5573_01 |
| TCGA_B0_4697_01 | TCGA_B2_5633_01 | TCGA_BP_4782_01 | TCGA_CJ_4885_01 | TCGA_DV_5574_01 |
| TCGA_B0_4698_01 | TCGA_B2_5635_01 | TCGA_BP_4784_01 | TCGA_CJ_4886_01 | TCGA_DV_5575_01 |
| TCGA_B0_4699_01 | TCGA_B2_5636_01 | TCGA_BP_4787_01 | TCGA_CJ_4887_01 | TCGA_DV_5576_01 |
| TCGA_B0_4700_01 | TCGA_B2_5639_01 | TCGA_BP_4789_01 | TCGA_CJ_4888_01 | TCGA_DV_A4VX_01 |
| TCGA_B0_4701_01 | TCGA_B2_5641_01 | TCGA_BP_4790_01 | TCGA_CJ_4889_01 | TCGA_DV_A4VZ_01 |
| TCGA_B0_4703_01 | TCGA_B2_A4SR_01 | TCGA_BP_4795_01 | TCGA_CJ_4890_01 | TCGA_DV_A4W0_01 |
| TCGA_B0_4706_01 | TCGA_B4_5377_01 | TCGA_BP_4797_01 | TCGA_CJ_4891_01 | TCGA_EU_5904_01 |
| TCGA_B0_4707_01 | TCGA_B4_5378_01 | TCGA_BP_4798_01 | TCGA_CJ_4892_01 | TCGA_EU_5905_01 |
| TCGA_B0_4710_01 | TCGA_B4_5832_01 | TCGA_BP_4799_01 | TCGA_CJ_4893_01 | TCGA_EU_5906_01 |
| TCGA_B0_4712_01 | TCGA_B4_5834_01 | TCGA_BP_4801_01 | TCGA_CJ_4894_01 | TCGA_EU_5907_01 |
| TCGA_B0_4713_01 | TCGA_B4_5835_01 | TCGA_BP_4803_01 | TCGA_CJ_4895_01 | TCGA_G6_A5PC_01 |
| TCGA_B0_4714_01 | TCGA_B4_5836_01 | TCGA_BP_4804_01 | TCGA_CJ_4897_01 | TCGA_G6_A8L6_01 |
| TCGA_B0_4718_01 | TCGA_B4_5838_01 | TCGA_BP_4807_01 | TCGA_CJ_4899_01 | TCGA_G6_A8L7_01 |
| TCGA_B0_4810_01 | TCGA_B4_5843_01 | TCGA_BP_4959_01 | TCGA_CJ_4900_01 | TCGA_G6_A8L8_01 |
| TCGA_B0_4811_01 | TCGA_B4_5844_01 | TCGA_BP_4960_01 | TCGA_CJ_4901_01 | TCGA_GK_A6C7_01 |
| TCGA_B0_4813_01 | TCGA_B8_4143_01 | TCGA_BP_4961_01 | TCGA_CJ_4902_01 | TCGA_MM_A563_01 |
| TCGA_B0_4814_01 | TCGA_B8_4146_01 | TCGA_BP_4962_01 | TCGA_CJ_4903_01 | TCGA_MM_A564_01 |
| TCGA_B0_4815_01 | TCGA_B8_4148_01 | TCGA_BP_4963_01 | TCGA_CJ_4904_01 | TCGA_MM_A84U_01 |
| TCGA_B0_4816_01 | TCGA_B8_4151_01 | TCGA_BP_4964_01 | TCGA_CJ_4905_01 | TCGA_MW_A4EC_01 |
| TCGA_B0_4817_01 | TCGA_B8_4153_01 | TCGA_BP_4965_01 | TCGA_CJ_4907_01 | TCGA_T7_A92I_01 |
| TCGA_B0_4818_01 | TCGA_B8_4154_01 | TCGA_BP_4967_01 | TCGA_CJ_4908_01 | |
| TCGA_B0_4819_01 | TCGA_B8_4619_01 | TCGA_BP_4968_01 | TCGA_CJ_4912_01 | |

**Table S2.** List of 72 normal samples.

| TCGA_A3_3358_11 | TCGA_B0_5709_11 | TCGA_CJ_5680_11 | TCGA_CZ_4863_11 | TCGA_CZ_5466_11 |
|---|---|---|---|---|
| TCGA_A3_3387_11 | TCGA_B0_5711_11 | TCGA_CJ_5681_11 | TCGA_CZ_4864_11 | TCGA_CZ_5467_11 |
| TCGA_B0_4700_11 | TCGA_B0_5712_11 | TCGA_CJ_5689_11 | TCGA_CZ_4865_11 | TCGA_CZ_5468_11 |
| TCGA_B0_4712_11 | TCGA_B2_5636_11 | TCGA_CJ_6030_11 | TCGA_CZ_5451_11 | TCGA_CZ_5469_11 |
| TCGA_B0_5402_11 | TCGA_B2_5641_11 | TCGA_CJ_6033_11 | TCGA_CZ_5452_11 | TCGA_CZ_5470_11 |
| TCGA_B0_5690_11 | TCGA_B8_4619_11 | TCGA_CW_5580_11 | TCGA_CZ_5453_11 | TCGA_CZ_5982_11 |
| TCGA_B0_5691_11 | TCGA_B8_4620_11 | TCGA_CW_5581_11 | TCGA_CZ_5454_11 | TCGA_CZ_5984_11 |
| TCGA_B0_5694_11 | TCGA_B8_4622_11 | TCGA_CW_5584_11 | TCGA_CZ_5455_11 | TCGA_CZ_5985_11 |
| TCGA_B0_5696_11 | TCGA_B8_5549_11 | TCGA_CW_5585_11 | TCGA_CZ_5456_11 | TCGA_CZ_5986_11 |
| TCGA_B0_5697_11 | TCGA_B8_5552_11 | TCGA_CW_5587_11 | TCGA_CZ_5457_11 | TCGA_CZ_5987_11 |
| TCGA_B0_5699_11 | TCGA_CJ_5672_11 | TCGA_CW_5589_11 | TCGA_CZ_5458_11 | TCGA_CZ_5988_11 |
| TCGA_B0_5701_11 | TCGA_CJ_5676_11 | TCGA_CW_5591_11 | TCGA_CZ_5461_11 | TCGA_CZ_5989_11 |
| TCGA_B0_5703_11 | TCGA_CJ_5677_11 | TCGA_CW_6087_11 | TCGA_CZ_5462_11 | |
| TCGA_B0_5705_11 | TCGA_CJ_5678_11 | TCGA_CW_6088_11 | TCGA_CZ_5463_11 | |
| TCGA_B0_5706_11 | TCGA_CJ_5679_11 | TCGA_CW_6090_11 | TCGA_CZ_5465_11 | |

**Table S3.** List of 296 HEntRNAs.

| **HEntRNA** | log 2FC$_{ntRNA}$ | log10FDR$_{ntRNA}$ | log10FDR$_{gene}$ |
|---|---|---|---|
| ABAT_33905_AA | 3.024196 | 38.29964 | 10.14857 |
| ABCA2_88247_RI | 2.848441 | 36.09568 | 0.062121 |
| ABCB8_82297_RI | 1.551236 | 39.02579 | 5.91575 |
| ABCB8_82298_RI | 1.803856 | 44.98714 | 5.91575 |
| ABCD4_28379_AA | 1.896292 | 45.41489 | 2.135012 |
| ABCF3_67845_AA | 2.141905 | 41.84423 | 8.99355 |
| ABHD3_44778_AD | 2.353552 | 85.42678 | 5.822957 |
| ACAA1_64015_AT | 2.615823 | 64.84883 | 9.7305 |
| ACAA1_64019_RI | 2.696236 | 84.7541 | 9.7305 |
| ACSM2A_99285_ES | 2.057707 | 53.06617 | 2.195604 |
| AGPAT4_78370_RI | 1.496707 | 36.72077 | 10.00137 |
| AKR1A1_2695_AT | 2.128289 | 69.75935 | 1.577134 |
| AKR1B1_81848_AD | 2.471052 | 45.18032 | 1.644613 |
| ALDH1L1_66589_ES | 2.08004 | 59.0253 | 7.209504 |
| ALDH2_24538_ES | 2.271407 | 39.61019 | 8.652131 |
| ALDH3A2_39759_ES | 3.238691 | 49.78073 | 8.070962 |
| ANGEL1_28558_AP | 1.308627 | 37.29019 | 5.29024 |
| ANO8_48279_AA | 1.829069 | 39.11718 | 8.903958 |
| AP3B2_32238_AT | 1.041681 | 47.1637 | 1.771349 |
| APPL2_24127_AP | 1.884248 | 48.43513 | 1.764063 |
| ARFIP2_14135_ES | 1.338411 | 41.47889 | 2.126422 |
| ARHGAP21_11012_ES | 1.875576 | 41.99585 | 1.361264 |
| ARHGAP32_19451_RI | 1.803196 | 69.24195 | 3.518725 |
| ARPC4_63182_ES | 1.469225 | 40.05207 | 0.12177 |
| ATMIN_37749_AP | 1.654411 | 37.28245 | 4.24262 |
| ATP6V0B_2509_RI | 1.803543 | 50.07319 | 4.83205 |
| BCAT2_50813_AT | 1.931115 | 46.62958 | 0.344357 |
| BLOC1S1_22230_AP | 1.710141 | 55.69965 | 4.58928 |
| BLOC1S1_22233_RI | 1.902104 | 47.43404 | 4.58928 |
| BRF1_29605_AP | 2.148036 | 69.92452 | 1.626097 |
| BSDC1_1591_RI | 1.661393 | 49.64427 | 0.928108 |
| BTBD8_3754_AT | 1.475675 | 57.39308 | 8.398894 |
| C11orf54_18326_ES | 1.643038 | 44.39177 | 6.008631 |
| C14orf2_29526_RI | 1.84195 | 49.83436 | 2.810086 |
| C14orf79_29585_AP | 1.074562 | 44.10106 | 0.399022 |
| C16orf58_36278_RI | 1.733395 | 43.44969 | 8.388519 |
| C21orf59_60356_RI | 1.601794 | 47.64174 | 0.009094 |
| C21orf59_60361_RI | 1.73421 | 48.03416 | 0.009094 |
| C9orf156_87024_AP | 1.541433 | 41.47017 | 4.780518 |

| | | | |
|---|---|---|---|
| CABIN1_61386_AP | 1.850782 | 45.40594 | 3.406388 |
| CALCOCO1_22108_RI | 2.255566 | 75.87747 | 2.974783 |
| CARM1_47600_ES | 1.686049 | 34.35193 | 0.573743 |
| CBWD2_55055_ES | 1.769887 | 50.09523 | 2.478041 |
| CCDC24_2525_RI | 1.228814 | 54.74916 | 2.287838 |
| CCDC30_2081_AP | 1.508678 | 58.90482 | 8.544673 |
| CEP68_53779_AT | 1.215185 | 52.37682 | 0.46046 |
| CEP68_53781_AD | 1.629673 | 38.71191 | 0.46046 |
| CIRBP_46432_RI | 1.613457 | 50.34683 | 2.473937 |
| CLNK_68788_AT | 1.054429 | 49.80375 | 0.492374 |
| COG4_37401_ES | 2.286571 | 39.83714 | 2.92724 |
| COMMD4_31850_AA | 2.052228 | 49.20222 | 3.240618 |
| COMT_61101_AA | 1.192999 | 34.58205 | 4.099023 |
| CREB3L2_81912_AT | 1.159181 | 34.48975 | 7.873967 |
| CTAGE5_27374_AP | 2.963367 | 67.65013 | 8.604698 |
| D2HGDH_58422_ES | 1.578844 | 39.22464 | 5.012904 |
| DAB2_71866_AD | 1.113784 | 37.36332 | 6.297864 |
| DCAF13_84821_AT | 1.761637 | 35.78612 | 6.621532 |
| DCAF8_8441_ES | 1.553507 | 57.22512 | 3.152392 |
| DCTN1_54046_RI | 1.84828 | 34.51915 | 0.888759 |
| DDRGK1_58577_AT | 2.035185 | 60.79492 | 3.891905 |
| DDX46_73426_ES | 2.417418 | 47.24384 | 5.500391 |
| DERA_20591_AP | 3.609052 | 76.78661 | 5.851893 |
| DERA_20595_AT | 1.797692 | 37.66826 | 5.851893 |
| DGAT1_85562_RI | 1.710848 | 50.06497 | 1.323892 |
| DHRS4_26779_AT | 1.658282 | 45.96846 | 5.694476 |
| DHRS4L2_26797_AT | 1.754671 | 34.4003 | 5.588037 |
| DNAJC10_56461_AT | 1.06219 | 55.74457 | 3.390777 |
| DNAJC4_16600_ES | 1.633521 | 37.82488 | 7.267472 |
| DRG2_39548_RI | 2.186314 | 58.47513 | 1.810853 |
| DYNC2LI1_53400_AA | 1.171245 | 69.66439 | 7.22675 |
| DYNC2LI1_53403_ES | 1.377139 | 82.95781 | 7.22675 |
| DYNLT1_78277_RI | 2.481773 | 51.88689 | 2.346046 |
| DYNLT1_78278_RI | 2.564187 | 56.01615 | 2.346046 |
| DZIP3_66037_RI | 2.338264 | 55.24462 | 1.836995 |
| ECHDC2_3019_AT | 1.323303 | 49.71189 | 2.31735 |
| ECHDC2_3020_AT | 1.173724 | 34.70424 | 2.31735 |
| ECHDC2_3021_AT | 1.389423 | 50.14053 | 2.31735 |
| EFR3B_52846_AT | 1.586104 | 50.38759 | 2.705658 |
| EIF3M_14849_RI | 1.644554 | 40.23202 | 9.503677 |
| EIF4A2_68054_ES | 1.923066 | 92.85635 | 2.223024 |
| EIF4G1_67894_RI | 2.189987 | 38.00471 | 1.329676 |
| ELF5_14951_AT | 4.665085 | 216.1325 | 6.422464 |

| | | | |
|---|---|---|---|
| ELMOD3_54246_RI | 1.548391 | 35.83687 | 9.672123 |
| ENOSF1_44459_AP | 1.415844 | 45.98305 | 0.044817 |
| ENOSF1_44474_ES | 2.012967 | 57.68517 | 0.044817 |
| EPS8_20581_AP | 1.723795 | 37.13235 | 10.15937 |
| EPS8L1_52012_ES | 1.069086 | 35.62704 | 8.294275 |
| EPS8L2_13731_RI | 2.124477 | 58.86505 | 2.19906 |
| ERAL1_40001_AD | 2.254933 | 40.16826 | 7.144701 |
| ERP29_24568_AP | 1.935706 | 39.11438 | 9.993459 |
| ESR1_78166_AT | 1.606983 | 39.67379 | 6.605249 |
| ETFA_31930_ES | 1.252154 | 37.71081 | 9.294656 |
| ETHE1_50198_AT | 1.912134 | 47.14787 | 0.224923 |
| FAM149A_71398_AP | 2.394443 | 66.56145 | 0.588201 |
| FAM162A_66433_AT | 1.618287 | 41.46937 | 9.937433 |
| FAM173A_32963_RI | 1.302731 | 38.9244 | 0.46047 |
| FAM192A_36525_AP | 1.683818 | 42.32046 | 3.756398 |
| FAM73B_87818_AD | 1.428577 | 39.20092 | 7.937716 |
| FAM73B_87819_AA | 2.102819 | 50.33188 | 7.937716 |
| FASTK_82333_RI | 1.619799 | 48.16946 | 9.28281 |
| FASTK_82334_RI | 1.316193 | 54.03384 | 9.28281 |
| FASTK_82335_RI | 1.783954 | 50.87433 | 9.28281 |
| FASTK_82336_RI | 1.603566 | 50.4551 | 9.28281 |
| FGD3_86889_AP | 2.041983 | 42.36843 | 5.264461 |
| FGD3_86895_RI | 1.715842 | 47.47726 | 5.264461 |
| FLAD1_7867_RI | 2.233935 | 55.23726 | 8.308244 |
| FOXRED1_19379_AD | 2.019014 | 41.96437 | 9.504087 |
| FUK_37385_AT | 1.381686 | 34.78616 | 2.612206 |
| GALK1_43496_RI | 1.859258 | 35.88082 | 0.185806 |
| GANAB_16377_AT | 1.795294 | 41.05331 | 2.389735 |
| GATM_30419_AT | 2.839312 | 56.69938 | 7.667683 |
| GET4_78552_AP | 1.456012 | 51.216 | 0.82408 |
| GLS2_22442_RI | 2.277279 | 60.17693 | 2.383725 |
| GPR116_76429_ES | 1.540522 | 50.61586 | 3.042824 |
| GPR56_36576_AP | -1.54515 | 79.06646 | 5.934634 |
| GSK3A_50137_AP | 1.064534 | 48.37607 | 2.177974 |
| GSS_59053_RI | 2.248318 | 59.51261 | 0.244555 |
| GSTM4_4055_RI | 1.883734 | 40.5669 | 9.549291 |
| GSTM4_4056_RI | 2.177108 | 42.2852 | 9.549291 |
| GTF2H1_14598_AP | 1.797063 | 52.23936 | 1.074371 |
| GTF2H2C_72386_AT | 1.039818 | 49.37504 | 4.572421 |
| GTF2IRD2_80093_AT | 1.23765 | 47.0078 | 2.590633 |
| GTF2IRD2B_80104_AT | 1.172909 | 47.02801 | 0.729904 |
| GTF3C5_88005_AT | 1.7615 | 55.52846 | 4.528212 |
| HARS2_73740_AT | 1.812809 | 47.49969 | 1.913949 |

| | | | |
|---|---|---|---|
| HARS2_73745_RI | 2.198562 | 77.67928 | 1.913949 |
| HDAC6_89011_RI | 2.516172 | 61.00044 | 2.327501 |
| HEATR6_42848_ES | 1.257536 | 35.58506 | 4.547757 |
| HECTD3_2592_RI | 2.640538 | 48.20682 | 3.58684 |
| HMBS_19098_RI | 1.323033 | 35.20716 | 0.269263 |
| HNRNPL_49699_ES | 1.165252 | 35.00127 | 2.607038 |
| HOOK2_47862_AP | 2.366573 | 90.88839 | 4.263409 |
| HOOK2_47868_AD | 1.783067 | 89.97882 | 4.263409 |
| IFT20_39879_AT | 1.275909 | 35.13027 | 1.987004 |
| IL11RA_86211_AT | 1.105121 | 42.31895 | 0.861696 |
| IL13RA1_89940_AP | 1.484682 | 37.29096 | 3.597331 |
| IL1R1_54770_AP | 1.203157 | 38.87957 | 4.405379 |
| IMPA1_84296_ES | 1.799225 | 44.44287 | 0.564943 |
| INTS3_7758_RI | 2.705354 | 76.65733 | 5.798183 |
| JTB_7776_RI | 1.389664 | 55.34938 | 1.355027 |
| KCNAB2_367_RI | 2.324407 | 42.11941 | 0.588844 |
| KIAA1407_66238_AD | 1.161752 | 37.99501 | 3.423705 |
| KIFC3_36609_AD | 1.588464 | 42.67927 | 9.242216 |
| KLC2_16990_AP | 1.754799 | 51.5138 | 6.002796 |
| KLC2_16992_RI | 2.304963 | 61.4167 | 6.002796 |
| KLHDC2_27443_RI | 1.951563 | 50.31634 | 3.801067 |
| KLHL3_73482_AT | 1.851218 | 75.72655 | 9.836923 |
| LTBP3_16861_AP | 1.306495 | 35.84903 | 0.120708 |
| MAEA_68465_AP | 2.217394 | 66.14887 | 4.412247 |
| MAGT1_89536_AT | 1.178687 | 64.49394 | 3.752831 |
| MBTD1_42521_RI | 1.756278 | 42.12122 | 5.272804 |
| METTL3_26596_RI | 1.012642 | 37.92424 | 7.026274 |
| MFSD1_67452_ES | 1.666424 | 38.87397 | 1.820182 |
| MFSD6_56566_AP | 1.419307 | 34.5128 | 7.476356 |
| MINK1_38600_ES | 2.160409 | 35.92569 | 0.755534 |
| MIOX_62834_AA | 1.410267 | 44.22425 | 6.128424 |
| MLXIP_24922_AP | 1.588766 | 46.51724 | 1.327338 |
| MON2_22832_AP | 2.258642 | 35.98527 | 1.130957 |
| MPDU1_38997_AT | 1.128616 | 36.68091 | 2.233113 |
| MRC2_42908_AP | 2.666847 | 38.35407 | 0.229561 |
| MRPL20_166_AT | 1.368384 | 41.73893 | 2.045862 |
| MRPL32_79325_AD | 1.763716 | 47.09443 | 0.924661 |
| MT1E_36479_RI | 2.081652 | 37.61578 | 3.401553 |
| MT1X_36493_AT | 2.351733 | 39.95009 | 4.446597 |
| MTCH1_75999_AT | 2.259877 | 73.09596 | 6.50039 |
| MYO19_40482_AA | 1.004459 | 58.48487 | 2.095364 |
| NAA40_16552_AA | 1.461801 | 34.8085 | 7.560718 |
| NDUFB2_82018_AP | 1.174302 | 35.84913 | 7.328392 |

| | | | |
|---|---|---|---|
| NDUFB8_12825_AT | 1.870719 | 40.88349 | 1.798626 |
| NDUFS3_15785_RI | 2.208794 | 44.97522 | 8.529923 |
| NDUFS7_46466_RI | 1.332018 | 42.48938 | 0.138348 |
| NDUFS8_17279_AD | 1.263671 | 40.11085 | 3.539822 |
| NFAT5_37288_AT | 1.030911 | 52.52677 | 8.167814 |
| NIPAL3_1109_AT | 1.411645 | 54.79725 | 8.413578 |
| NOP58_56946_ES | 1.527595 | 45.94242 | 10.17813 |
| NPEPPS_42080_AD | 1.915414 | 65.93617 | 1.186741 |
| NPEPPS_42084_ES | 1.611017 | 55.87752 | 1.186741 |
| NPRL2_65034_RI | 2.528435 | 63.97018 | 3.93306 |
| NPRL2_65037_RI | 2.52884 | 73.40496 | 3.93306 |
| NR4A1_21887_AP | 1.826139 | 36.07856 | 2.713627 |
| OSGEP_26442_AD | 1.457656 | 39.02982 | 8.074937 |
| OSGEP_26443_RI | 1.674608 | 45.09196 | 8.074937 |
| PARP2_26426_RI | 1.475583 | 39.84413 | 2.543534 |
| PARVA_14430_AT | 2.20077 | 36.14335 | 6.203322 |
| PBXIP1_7843_AT | 2.138785 | 36.58571 | 5.045193 |
| PCBP2_22046_AT | 1.626906 | 46.85918 | 0.960894 |
| PCTP_42588_AA | 1.204755 | 37.89182 | 0.939734 |
| PCYT1A_68233_AA | 1.719919 | 38.6039 | 0.112209 |
| PDPR_37331_ES | 1.737009 | 39.64727 | 2.017122 |
| PDPR_37332_AA | 1.090677 | 35.00501 | 2.017122 |
| PDZD3_100577_RI | 1.244998 | 46.4893 | 0.237482 |
| PHF12_40025_RI | 1.385763 | 44.73 | 5.468385 |
| PHF12_40026_RI | 1.292906 | 53.12697 | 5.468385 |
| PLEKHH2_53393_ES | 1.192724 | 41.60306 | 1.470348 |
| PLEKHM3_57206_AT | 2.024024 | 34.8781 | 6.071314 |
| PLSCR1_67157_AT | 1.245285 | 35.73641 | 0.109843 |
| PMPCA_88166_AT | 1.96691 | 42.27771 | 3.723676 |
| POLB_83706_AP | 2.269689 | 43.66684 | 2.384992 |
| PPIL4_78083_AP | 2.005113 | 37.41032 | 8.73485 |
| PPP2R4_87835_AP | 1.576669 | 46.88269 | 5.410617 |
| PSMA2_79317_AT | 1.203918 | 60.5869 | 9.648639 |
| PSMA7_60044_AT | 1.828876 | 56.78339 | 3.876145 |
| PSMB7_87531_AT | 1.272464 | 36.83898 | 7.664151 |
| PTK7_76245_AT | 1.040428 | 47.68613 | 5.784017 |
| RAB11B_47227_AT | 1.196865 | 60.45356 | 6.039839 |
| RABEP2_35894_ES | 1.200911 | 43.15293 | 5.469229 |
| RASA1_72717_ES | 1.913051 | 37.21102 | 1.280337 |
| RBM6_64936_RI | 1.456512 | 43.65651 | 1.662491 |
| RBM7_18823_RI | 1.005994 | 34.30278 | 0.71867 |
| RCOR3_9711_ES | 1.098587 | 38.99151 | 0.380824 |
| RFX5_7609_ES | 1.517257 | 57.26862 | 1.071632 |

| | | | |
|---|---|---|---|
| RIC8B_24156_AP | 1.724389 | 37.4465 | 1.907232 |
| RMND1_78158_AD | 1.209889 | 53.18362 | 0.998501 |
| RNF216_78674_AA | 3.495888 | 46.24212 | 4.039863 |
| RNF4_68571_AT | 1.909733 | 45.8812 | 1.510495 |
| ROGDI_33857_AD | 2.607522 | 57.87275 | 5.099435 |
| RPAP1_30095_RI | 1.843081 | 40.89188 | 2.316742 |
| RRM2B_84774_ES | 1.397889 | 59.67963 | 2.260299 |
| RSAD1_42400_AD | 1.750304 | 35.08452 | 1.080659 |
| RWDD2A_76867_RI | 1.098034 | 35.08818 | 2.510196 |
| SAT1_88697_AT | 1.334909 | 40.32939 | 0.026127 |
| SEC24C_12174_RI | 1.930012 | 97.69473 | 3.259443 |
| SERP1_67272_AT | 1.609945 | 38.38999 | 2.223193 |
| SGSM2_38395_RI | 1.919237 | 57.75219 | 4.004276 |
| SGSM3_62346_RI | 1.80325 | 65.064 | 0.065758 |
| SH3YL1_52491_AP | 1.892284 | 35.8778 | 8.100098 |
| SH3YL1_52495_AP | 1.809885 | 77.25702 | 8.100098 |
| SLC23A3_57600_AD | 1.897054 | 40.86007 | 0.453574 |
| SLC25A46_72943_AP | 1.923962 | 42.02293 | 5.572627 |
| SLC35A4_73686_AP | 2.194849 | 36.33893 | 3.931704 |
| SLC39A13_15746_RI | 2.178064 | 39.7108 | 4.111019 |
| SLC39A13_15747_RI | 1.828466 | 54.23622 | 4.111019 |
| SLC39A13_15748_RI | 2.483085 | 52.14755 | 4.111019 |
| SLC3A2_16463_AP | 2.417293 | 77.44249 | 4.247983 |
| SLC6A6_63527_AT | 1.447641 | 37.83154 | 2.791388 |
| SLC9A1_1313_AT | 1.231407 | 41.01963 | 9.667517 |
| SMARCD3_82363_RI | 1.55755 | 62.25521 | 1.749625 |
| SNAP23_30179_AA | 2.711035 | 45.67288 | 1.656646 |
| SNX13_78881_RI | 1.719325 | 47.34655 | 5.043275 |
| SNX29_34058_ES | 1.236321 | 37.7738 | 4.35009 |
| SORD_30379_AT | 2.425074 | 57.8192 | 9.712734 |
| SOX15_39009_AT | 1.565721 | 42.65722 | 0.914532 |
| SPINK1_73964_AT | 2.931095 | 47.29951 | 1.853148 |
| SRGAP1_22854_AP | 1.456364 | 35.4124 | 0.963846 |
| SRI_80359_ES | 2.382843 | 43.87881 | 0.768734 |
| SSB_55885_ES | 1.153132 | 40.54384 | 2.186569 |
| SSH3_17164_RI | 1.871071 | 49.56862 | 0.029845 |
| ST7_81554_AP | 1.656989 | 39.72804 | 1.240675 |
| STC1_83086_AP | 2.816583 | 51.20452 | 2.059306 |
| STK40_1772_RI | 2.122027 | 44.04214 | 0.327306 |
| STXBP2_47123_AP | 1.354934 | 38.31124 | 10.18139 |
| SVEP1_87186_AT | 1.554393 | 48.09523 | 8.863849 |
| TAOK3_24699_AP | 2.284799 | 52.5523 | 1.566512 |
| TARDBP_635_ES | 1.423834 | 46.36402 | 1.186516 |

| | | | |
|---|---|---|---|
| TBC1D17_51116_ES | 1.931543 | 73.30188 | 0.495683 |
| TCEA2_60189_RI | 1.81782 | 36.89414 | 1.014816 |
| TECR_47993_AP | 2.720282 | 42.05967 | 1.827707 |
| TIMM17B_89018_AA | 1.215419 | 50.98386 | 0.49096 |
| TIMP2_43923_AD | 2.030922 | 35.67453 | 0.951188 |
| TM2D1_3230_AT | 1.398182 | 62.14637 | 3.518649 |
| TMEM126B_18114_RI | 1.665887 | 41.51325 | 2.724765 |
| TMEM138_16188_RI | 1.423426 | 42.67927 | 8.417306 |
| TMEM14B_75311_AT | 1.369089 | 39.79094 | 6.018907 |
| TRAP1_33617_AP | 1.573402 | 39.05323 | 8.734967 |
| TRAPPC3_1752_ES | 2.026466 | 37.28866 | 0.209641 |
| TRIM4_80863_AT | 1.236214 | 45.47357 | 1.937357 |
| TTC38_62703_ES | 1.375332 | 45.21327 | 4.103259 |
| TUBGCP2_13530_AP | 1.298718 | 39.45535 | 5.528709 |
| TUBGCP2_13535_AA | 2.737763 | 44.21283 | 5.528709 |
| UBE2G2_60831_ES | 2.20977 | 61.24254 | 0.161965 |
| UBE2N_23689_RI | 1.805154 | 37.68085 | 4.145737 |
| UBE3B_24310_ES | 2.563909 | 35.54806 | 8.08291 |
| UBR4_883_RI | 3.029953 | 35.72437 | 4.718675 |
| UBXN4_55449_RI | 2.015936 | 34.78827 | 1.028204 |
| UQCR11_46529_AT | 2.055202 | 37.94205 | 0.292275 |
| USP40_58045_AP | 2.53322 | 44.10991 | 1.075956 |
| USP48_994_AP | 1.188987 | 45.80544 | 3.646375 |
| VAC14_37411_AP | 1.865808 | 34.73692 | 0.601084 |
| VGLL3_65659_AT | 2.691891 | 48.1648 | 4.284287 |
| VPS28_85601_RI | 1.548317 | 43.83286 | 7.491276 |
| VPS28_85602_RI | 2.33547 | 55.64056 | 7.491276 |
| VPS28_85604_RI | 2.418835 | 57.95977 | 7.491276 |
| VPS28_85611_RI | 1.843844 | 46.02359 | 7.491276 |
| VPS8_67976_RI | 2.180572 | 57.50545 | 0.226443 |
| VRK3_51142_AT | 1.69176 | 56.67209 | 0.016403 |
| WAC_11103_ES | 1.880047 | 43.31168 | 0.045828 |
| WBSCR22_80000_AT | 1.700616 | 46.24492 | 0.341137 |
| XBP1_61561_AP | 1.428561 | 37.70344 | 3.023477 |
| ZNF20_47777_AT | 1.164256 | 44.60443 | 8.606455 |
| ZNF24_45164_AA | 1.394423 | 35.80339 | 5.923833 |
| ZNF317_47268_AA | 2.019902 | 35.93944 | 3.053495 |
| ZNF638_53926_AP | 2.032338 | 37.31461 | 0.135991 |
| ZNF675_48823_AT | 1.635358 | 62.73052 | 1.486068 |
| ZNF76_75899_RI | 1.910763 | 56.45873 | 3.470216 |
| ZNF76_75900_RI | 2.351879 | 61.58282 | 3.470216 |
| ZNRF1_37577_AT | 1.600073 | 43.58074 | 10.18139 |

**Table S4.** ntRNA-miRNA interaction pairs from Targetscan database.

| ntRNA | miRNA |
|---|---|
| PPIL4_78083_AP(ENST00000340881) | hsa-miR-499a-5p |
| PPIL4_78083_AP(ENST00000340881) | hsa-miR-499a-5p |
| BSDC1_1591_RI(ENST00000341071) | hsa-miR-25-3p |
| GSTM4_4056_RI(ENST00000369833) | hsa-miR-378a-3p |
| GSTM4_4056_RI(ENST00000369833) | hsa-miR-378h |
| HECTD3_2592_RI(ENST00000372168) | hsa-miR-26b-5p |
| KLC2_16990_AP(ENST00000394065) | hsa-miR-34a-5p |
| KLC2_16992_RI(ENST00000394065) | hsa-miR-34a-5p |
| KLC2_16990_AP(ENST00000394065) | hsa-miR-143-3p |
| KLC2_16992_RI(ENST00000394065) | hsa-miR-143-3p |
| KLC2_16990_AP(ENST00000394065) | hsa-miR-148a-3p |
| KLC2_16990_AP(ENST00000394065) | hsa-miR-148b-3p |
| KLC2_16992_RI(ENST00000394065) | hsa-miR-148a-3p |
| KLC2_16992_RI(ENST00000394065) | hsa-miR-148b-3p |
| KLC2_16990_AP(ENST00000394065) | hsa-miR-125b-5p |
| KLC2_16992_RI(ENST00000394065) | hsa-miR-125b-5p |
| SNX13_78881_RI(ENST00000409389) | hsa-miR-25-3p |
| SLC39A13_15746_RI(ENST00000524928) | hsa-miR-125b-5p |
| SLC39A13_15747_RI(ENST00000524928) | hsa-miR-125b-5p |
| SLC39A13_15746_RI(ENST00000524928) | hsa-miR-27b-3p |
| SLC39A13_15747_RI(ENST00000524928) | hsa-miR-27b-3p |
| PARP2_26426_RI(ENST00000527915) | hsa-miR-140-3p.2 |
| TMEM126B_18114_RI(ENST00000534341) | hsa-miR-34a-5p |
| RBM7_18823_RI(ENST00000541475) | hsa-miR-186-5p |
| RBM7_18823_RI(ENST00000541475) | hsa-miR-125b-5p |
| RBM7_18823_RI(ENST00000541475) | hsa-miR-143-3p |

| | |
|---|---|
| CALCOCO1_22108_RI(ENST00000548263) | hsa-miR-374b-5p |
| UBE2N_23689_RI(ENST00000550657) | hsa-miR-27b-3p |
| UBE2N_23689_RI(ENST00000550657) | hsa-miR-221-3p |
| UBE2N_23689_RI(ENST00000550657) | hsa-miR-499a-5p |
| ABAT_33905_AA(ENST00000569156) | hsa-miR-125b-5p |
| ABAT_33905_AA(ENST00000569156) | hsa-miR-27b-3p |
| ABAT_33905_AA(ENST00000569156) | hsa-miR-421 |
| ABAT_33905_AA(ENST00000569156) | hsa-miR-499a-5p |
| ABAT_33905_AA(ENST00000569156) | hsa-miR-374b-5p |
| PCTP_42588_AA(ENST00000569156) | hsa-miR-125b-5p |
| PCTP_42588_AA(ENST00000569156) | hsa-miR-421 |
| PHF12_40026_RI(ENST00000577226) | hsa-miR-140-3p.2 |
| PHF12_40025_RI(ENST00000577226) | hsa-miR-140-3p.2 |
| PHF12_40026_RI(ENST00000577226) | hsa-miR-221-3p |
| PHF12_40025_RI(ENST00000577226) | hsa-miR-221-3p |
| ZNF24_45164_AA(ENST00000592278) | hsa-miR-499a-5p |
| ZNF24_45164_AA(ENST00000592278) | hsa-miR-140-3p.2 |
| ZNF24_45164_AA(ENST00000592278) | hsa-miR-199a-5p |

**Table S5.** miRNA-lncRNA interaction pairs from LncBase2 database.

| miRNA | lncRNA | miRNA | lncRNA | miRNA | lncRNA |
|---|---|---|---|---|---|
| hsa-miR-27b-3p | RP11-368I23.2 | hsa-miR-140-3p | LINC00645 | hsa-miR-378a-3p | ANKRD10-IT1 |
| hsa-miR-143-3p | LINC00705 | hsa-miR-199a-5p | LINC00645 | hsa-miR-221-3p | RP4-568C11.4 |
| hsa-miR-27b-3p | LINC00705 | hsa-miR-186-5p | RP11-8P13.5 | hsa-miR-26b-5p | RP4-568C11.4 |
| hsa-miR-186-5p | COLCA1 | hsa-miR-26b-5p | RP11-8P13.5 | hsa-miR-499a-5p | RP4-568C11.4 |
| hsa-miR-378a-3p | COLCA1 | hsa-miR-26b-5p | CTC-492K19.4 | hsa-miR-148a-3p | RP4-568C11.4 |
| hsa-miR-378h | CTD-3099C6.5 | hsa-miR-34a-5p | RP3-522J7.6 | hsa-miR-148b-3p | RP4-568C11.4 |
| hsa-miR-378a-3p | CTD-3099C6.5 | hsa-miR-186-5p | CTD-2349P21.11 | hsa-miR-378a-3p | RP4-568C11.4 |
| hsa-miR-146b-5p | RP11-63P12.7 | hsa-miR-140-3p | CTD-2012J19.3 | hsa-miR-199a-5p | CHRM3-AS2 |
| hsa-miR-374b-5p | RP5-940J5.8 | hsa-miR-26b-5p | CTD-3051D23.1 | hsa-miR-186-5p | CHRM3-AS2 |
| hsa-miR-143-3p | AP001610.5 | hsa-miR-143-3p | CTD-3051D23.1 | hsa-miR-374b-5p | CHRM3-AS2 |
| hsa-miR-186-5p | CTD-2192J16.21 | hsa-miR-34a-5p | AC097713.4 | hsa-miR-34a-5p | RP11-328N19.1 |
| hsa-miR-499a-5p | AC003090.1 | hsa-miR-146b-5p | AC104699.1 | hsa-miR-148b-3p | RP11-118K6.3 |
| hsa-miR-125b-5p | AC003090.1 | hsa-miR-27b-3p | RP11-286B14.1 | hsa-miR-378h | RP11-37B2.1 |
| hsa-miR-125b-5p | AC008079.10 | hsa-miR-221-3p | RP11-286B14.1 | hsa-miR-378a-3p | RP11-37B2.1 |
| hsa-miR-199a-5p | AC008079.10 | hsa-miR-27b-3p | RP11-383I23.2 | hsa-miR-374b-5p | RP11-37B2.1 |
| hsa-miR-34a-5p | AC008079.10 | hsa-miR-140-3p | RP11-706O15.7 | hsa-miR-140-3p | RP11-37B2.1 |
| hsa-miR-143-3p | AC008079.10 | hsa-miR-26b-5p | RP11-706O15.7 | hsa-miR-186-5p | RP11-37B2.1 |
| hsa-miR-143-3p | AC093326.1 | hsa-miR-143-3p | RP11-706O15.7 | hsa-miR-374b-5p | LINC01320 |
| hsa-miR-148a-3p | RP11-539L10.2 | hsa-miR-34a-5p | MIR99AHG | hsa-miR-221-3p | LINC01320 |
| hsa-miR-148b-3p | RP11-539L10.2 | hsa-miR-148a-3p | RP11-146E13.4 | hsa-miR-378h | RP11-342D11.2 |
| hsa-miR-143-3p | RP4-794I6.4 | hsa-miR-146b-5p | RP11-554D20.2 | hsa-miR-378a-3p | RP11-342D11.2 |
| hsa-miR-199a-5p | RP4-794I6.4 | hsa-miR-421 | LINC01030 | hsa-miR-34a-5p | RP11-61I13.3 |
| hsa-miR-34a-5p | RBMS3-AS1 | hsa-miR-26b-5p | AC007750.5 | hsa-miR-125b-5p | RP11-61I13.3 |
| hsa-miR-499a-5p | P4HA2-AS1 | hsa-miR-140-3p | AC007750.5 | hsa-miR-143-3p | RP11-156L14.1 |
| hsa-miR-27b-3p | KC6 | hsa-miR-27b-3p | CTA-228A9.4 | hsa-miR-27b-3p | RP11-156L14.1 |
| hsa-miR-34a-5p | RP11-264B14.2 | hsa-miR-148a-3p | CTA-228A9.4 | hsa-miR-148a-3p | RP11-156L14.1 |

| | | | | | |
|---|---|---|---|---|---|
| hsa-miR-25-3p | LINC01322 | hsa-miR-143-3p | CTA-228A9.4 | hsa-miR-186-5p | RP11-156L14.1 |
| hsa-miR-186-5p | RP4-740C4.7 | hsa-miR-148b-3p | CTA-228A9.4 | hsa-miR-125b-5p | RP11-156L14.1 |
| hsa-miR-34a-5p | MYCNOS | hsa-miR-26b-5p | AC000068.5 | hsa-miR-143-3p | LINC00882 |
| hsa-miR-186-5p | RP13-514E23.1 | hsa-miR-26b-5p | RP11-310E22.5 | hsa-miR-146b-5p | RP11-1151B14.2 |
| hsa-miR-34a-5p | AC006960.7 | hsa-miR-25-3p | RP11-900F13.2 | hsa-miR-378a-3p | RP11-1151B14.2 |
| hsa-miR-125b-5p | RP11-700H6.1 | hsa-miR-146b-5p | RP4-539M6.20 | hsa-miR-378h | RP11-1151B14.2 |
| hsa-miR-34a-5p | AC016722.4 | hsa-miR-199a-5p | RP11-324I22.2 | hsa-miR-25-3p | RP11-1B20.1 |
| hsa-miR-148a-3p | AC016722.4 | hsa-miR-125b-5p | RP11-686D22.5 | hsa-miR-148b-3p | RP11-624C23.1 |
| hsa-miR-421 | AC016722.4 | hsa-miR-499a-5p | EXTL3-AS1 | hsa-miR-148a-3p | RP11-624C23.1 |
| hsa-miR-148b-3p | AC016722.4 | hsa-miR-143-3p | EXTL3-AS1 | hsa-miR-186-5p | RP11-382J12.1 |
| hsa-miR-148b-3p | RP11-540K16.1 | hsa-miR-186-5p | RP11-707A18.1 | hsa-miR-374b-5p | RP11-382J12.1 |
| hsa-miR-27b-3p | RRS1-AS1 | hsa-miR-374b-5p | RP11-707A18.1 | hsa-miR-186-5p | RP11-133F8.2 |
| hsa-miR-499a-5p | RP4-681N20.5 | hsa-miR-27b-3p | RP11-707A18.1 | hsa-miR-143-3p | RP11-133F8.2 |
| hsa-miR-143-3p | SACS-AS1 | hsa-miR-143-3p | RP1-102G20.5 | hsa-miR-374b-5p | RP11-196G18.3 |
| hsa-miR-34a-5p | RP13-126P21.2 | hsa-miR-140-3p | CTD-2529P6.3 | hsa-miR-143-3p | ATP11A-AS1 |
| hsa-miR-186-5p | RP11-117D22.2 | hsa-miR-186-5p | AC002519.8 | hsa-miR-143-3p | RP11-356B19.11 |
| hsa-miR-34a-5p | IQCH-AS1 | hsa-miR-26b-5p | AC002519.8 | hsa-miR-143-3p | AC091814.3 |
| hsa-miR-140-3p | IQCH-AS1 | hsa-miR-421 | AC002519.8 | hsa-miR-186-5p | AC107079.1 |
| hsa-miR-146b-5p | RP11-319G9.3 | hsa-miR-27b-3p | RP11-196G18.22 | hsa-miR-146b-5p | RP11-473O4.5 |
| hsa-miR-186-5p | RP11-319G9.3 | hsa-miR-27b-3p | RP11-324D17.3 | hsa-miR-25-3p | RP5-965G21.4 |
| hsa-miR-143-3p | RP6-159A1.4 | hsa-miR-186-5p | RP11-399E6.1 | hsa-miR-148b-3p | RP5-965G21.4 |
| hsa-miR-148b-3p | RP6-159A1.4 | hsa-miR-146b-5p | RP11-507K2.2 | hsa-miR-148a-3p | RP5-965G21.4 |
| hsa-miR-148a-3p | RP6-159A1.4 | hsa-miR-125b-5p | NCAM1-AS1 | hsa-miR-143-3p | RP5-965G21.4 |
| hsa-miR-125b-5p | RP6-159A1.4 | hsa-miR-25-3p | NCAM1-AS1 | hsa-miR-221-3p | AC007879.7 |
| hsa-miR-186-5p | RP6-159A1.4 | hsa-miR-421 | LRP4-AS1 | hsa-miR-199a-5p | AP000265.1 |
| hsa-miR-186-5p | LINC01208 | hsa-miR-378h | LRP4-AS1 | hsa-miR-499a-5p | RP11-526A4.1 |
| hsa-miR-27b-3p | RP11-521D12.5 | hsa-miR-378a-3p | LRP4-AS1 | hsa-miR-146b-5p | ZBED5-AS1 |
| hsa-miR-26b-5p | FAM95B1 | hsa-miR-34a-5p | LRP4-AS1 | hsa-miR-125b-5p | RP11-421E14.2 |

| | | | | | |
|---|---|---|---|---|---|
| hsa-miR-143-3p | FAM95B1 | hsa-miR-499a-5p | RP11-105N14.1 | hsa-miR-221-3p | RP11-6O2.4 |
| hsa-miR-26b-5p | AC000036.4 | hsa-miR-34a-5p | RP11-192P3.4 | hsa-miR-26b-5p | RP11-330M19.1 |
| hsa-miR-143-3p | AC000036.4 | hsa-miR-140-3p | AP000355.2 | hsa-miR-221-3p | RP11-544A12.8 |
| hsa-miR-27b-3p | AC058791.1 | hsa-miR-146b-5p | CTD-2015G9.2 | hsa-miR-148b-3p | AC004901.1 |
| hsa-miR-34a-5p | TOLLIP-AS1 | hsa-miR-186-5p | RP1-149A16.19 | hsa-miR-148a-3p | AC004901.1 |
| hsa-miR-143-3p | CTB-193M12.3 | hsa-miR-421 | CTD-2058B24.2 | hsa-miR-199a-5p | RP4-683M8.2 |
| hsa-miR-499a-5p | CTD-2026J24.1 | hsa-miR-374b-5p | RP11-79M19.2 | hsa-miR-186-5p | RP5-1057J7.7 |
| hsa-miR-34a-5p | RP11-231I16.1 | hsa-miR-186-5p | RP11-79M19.2 | hsa-miR-146b-5p | RP5-1056H1.2 |
| hsa-miR-27b-3p | RP11-231I16.1 | hsa-miR-140-3p | LINC01561 | hsa-miR-199a-5p | RP5-1056H1.2 |
| hsa-miR-499a-5p | RP11-254F7.3 | hsa-miR-27b-3p | LINC01561 | hsa-miR-186-5p | CTC-327F10.4 |
| hsa-miR-125b-5p | TRPM2-AS | hsa-miR-125b-5p | LINC00987 | hsa-miR-34a-5p | AF131215.3 |
| hsa-miR-34a-5p | TRPM2-AS | hsa-miR-143-3p | CYP4F35P | hsa-miR-26b-5p | AF131215.3 |
| hsa-miR-27b-3p | L3MBTL4-AS1 | hsa-miR-186-5p | RP11-575L7.4 | hsa-miR-34a-5p | AC068282.3 |
| hsa-miR-374b-5p | CTD-2260A17.1 | hsa-miR-34a-5p | RP11-575L7.4 | hsa-miR-140-3p | RP11-115D19.1 |
| hsa-miR-27b-3p | RP11-677I18.3 | hsa-miR-146b-5p | RP5-1042K10.10 | hsa-miR-125b-5p | RP11-115D19.1 |
| hsa-miR-125b-5p | RP11-420A23.1 | hsa-miR-186-5p | RP3-467L1.6 | hsa-miR-34a-5p | EAF1-AS1 |
| hsa-miR-378a-3p | LINC00626 | hsa-miR-186-5p | RP11-342K6.4 | hsa-miR-499a-5p | EAF1-AS1 |
| hsa-miR-378h | LINC00626 | hsa-miR-34a-5p | RP11-114M1.1 | hsa-miR-26b-5p | EAF1-AS1 |
| hsa-miR-27b-3p | RP11-166P13.3 | hsa-miR-140-3p | RP11-114M1.1 | hsa-miR-143-3p | CTD-2199O4.1 |
| hsa-miR-26b-5p | LINC01020 | hsa-miR-34a-5p | RP11-206M11.7 | hsa-miR-27b-3p | RP11-283I3.1 |
| hsa-miR-186-5p | CTC-484P3.3 | hsa-miR-27b-3p | CTD-2349P21.5 | hsa-miR-146b-5p | RP11-255H23.4 |
| hsa-miR-143-3p | CTC-484P3.3 | hsa-miR-148b-3p | RP11-394I13.1 | hsa-miR-186-5p | CTD-2095E4.5 |
| hsa-miR-421 | CTC-484P3.3 | hsa-miR-148a-3p | RP11-394I13.1 | hsa-miR-374b-5p | MIR503HG |
| hsa-miR-221-3p | RP4-564F22.7 | hsa-miR-125b-5p | RP11-59C5.3 | hsa-miR-27b-3p | CTD-2270F17.1 |
| hsa-miR-125b-5p | MLIP-AS1 | hsa-miR-27b-3p | IGF2BP2-AS1 | hsa-miR-26b-5p | RP11-147L13.13 |
| hsa-miR-499a-5p | MLIP-AS1 | hsa-miR-186-5p | CTD-2521M24.6 | hsa-miR-143-3p | RP11-276E17.2 |
| hsa-miR-221-3p | MLIP-AS1 | hsa-miR-378h | PLA2G4E-AS1 | hsa-miR-148b-3p | AC003991.3 |
| hsa-miR-148b-3p | RP11-3D4.3 | hsa-miR-378a-3p | PLA2G4E-AS1 | hsa-miR-148a-3p | AC003991.3 |

| | | | | | |
|---|---|---|---|---|---|
| hsa-miR-148a-3p | RP11-3D4.3 | hsa-miR-499a-5p | PLA2G4E-AS1 | hsa-miR-27b-3p | AC007036.6 |
| hsa-miR-186-5p | IFNG-AS1 | hsa-miR-27b-3p | CTD-3037G24.3 | hsa-miR-421 | PCAT29 |
| hsa-miR-143-3p | AC091814.2 | hsa-miR-374b-5p | LINC01393 | hsa-miR-378a-3p | LINC01138 |
| hsa-miR-146b-5p | LINC00607 | hsa-miR-186-5p | LINC01393 | hsa-miR-378h | LINC01138 |
| hsa-miR-421 | LINC00487 | hsa-miR-27b-3p | RP11-760H22.2 | hsa-miR-34a-5p | LINC01138 |
| hsa-miR-186-5p | LINC00487 | hsa-miR-421 | NUCB1-AS1 | hsa-miR-199a-5p | LINC01138 |
| hsa-miR-374b-5p | RP11-276H19.1 | hsa-miR-146b-5p | RP11-120K24.3 | hsa-miR-421 | CPEB2-AS1 |
| hsa-miR-27b-3p | RP11-276H19.1 | hsa-miR-26b-5p | LINC00837 | hsa-miR-221-3p | CPEB2-AS1 |
| hsa-miR-186-5p | RP11-276H19.1 | hsa-miR-125b-5p | LINC00837 | hsa-miR-27b-3p | CPEB2-AS1 |
| hsa-miR-221-3p | RP11-234G16.5 | hsa-miR-125b-5p | AC004988.1 | hsa-miR-374b-5p | CPEB2-AS1 |
| hsa-miR-148a-3p | RP4-644L1.2 | hsa-miR-25-3p | RP11-861L17.3 | hsa-miR-125b-5p | RP11-501J20.5 |
| hsa-miR-148b-3p | RP4-644L1.2 | hsa-miR-499a-5p | RP11-861L17.3 | hsa-miR-148a-3p | RP4-684O24.5 |
| hsa-miR-125b-5p | RP4-644L1.2 | hsa-miR-374b-5p | RP11-471M2.3 | hsa-miR-26b-5p | RP11-136K14.3 |
| hsa-miR-26b-5p | RP4-644L1.2 | hsa-miR-499a-5p | RP11-471M2.3 | hsa-miR-374b-5p | LINC00473 |
| hsa-miR-374b-5p | DLEU2L | hsa-miR-143-3p | RP5-881L22.5 | hsa-miR-146b-5p | AC007278.2 |
| hsa-miR-27b-3p | DLEU2L | hsa-miR-186-5p | RP11-713C5.1 | hsa-miR-146b-5p | RP11-479J7.2 |
| hsa-miR-125b-5p | RP11-184M15.1 | hsa-miR-374b-5p | RP11-713C5.1 | hsa-miR-125b-5p | RP11-181E10.3 |
| hsa-miR-27b-3p | RP11-184M15.1 | hsa-miR-140-3p | RP11-47J17.2 | hsa-miR-26b-5p | RP11-181E10.3 |
| hsa-miR-125b-5p | RP11-348N5.9 | hsa-miR-499a-5p | RNF219-AS1 | hsa-miR-499a-5p | TNKS2-AS1 |
| hsa-miR-25-3p | RP11-348N5.9 | hsa-miR-186-5p | RNF219-AS1 | hsa-miR-34a-5p | RP11-50D9.3 |
| hsa-miR-186-5p | RP11-348N5.9 | hsa-miR-27b-3p | RNF219-AS1 | hsa-miR-499a-5p | RP11-50D9.3 |
| hsa-miR-378a-3p | LINC01556 | hsa-miR-25-3p | RNF219-AS1 | hsa-miR-374b-5p | RP11-50D9.3 |
| hsa-miR-378h | LINC01556 | hsa-miR-374b-5p | RNF219-AS1 | hsa-miR-378h | RP11-346C20.3 |
| hsa-miR-186-5p | AC009120.11 | hsa-miR-199a-5p | RNF219-AS1 | hsa-miR-378a-3p | RP11-346C20.3 |
| hsa-miR-125b-5p | RP11-401P9.4 | hsa-miR-26b-5p | RNF219-AS1 | hsa-miR-146b-5p | AP000688.29 |
| hsa-miR-186-5p | RP11-506O24.2 | hsa-miR-374b-5p | RP13-143G15.4 | hsa-miR-378h | AP000688.29 |
| hsa-miR-27b-3p | CTD-2376I20.1 | hsa-miR-148a-3p | CTD-2024I7.18 | hsa-miR-378a-3p | AP000688.29 |
| hsa-miR-27b-3p | CTC-429L19.3 | hsa-miR-148b-3p | CTD-2024I7.18 | hsa-miR-221-3p | AP000688.29 |

| | | | | | |
|---|---|---|---|---|---|
| hsa-miR-125b-5p | PRMT5-AS1 | hsa-miR-221-3p | MIAT | hsa-miR-140-3p | AP000688.29 |
| hsa-miR-140-3p | PRMT5-AS1 | hsa-miR-27b-3p | MIAT | hsa-miR-186-5p | CLRN1-AS1 |
| hsa-miR-221-3p | PRMT5-AS1 | hsa-miR-125b-5p | MIAT | hsa-miR-27b-3p | CLRN1-AS1 |
| hsa-miR-199a-5p | RP11-131L23.1 | hsa-miR-378h | MIAT | hsa-miR-378h | RP11-168O16.1 |
| hsa-miR-186-5p | RP11-131L23.1 | hsa-miR-378a-3p | MIAT | hsa-miR-378a-3p | RP11-168O16.1 |
| hsa-miR-140-3p | RP11-2E11.5 | hsa-miR-143-3p | RP11-219G17.4 | hsa-miR-186-5p | RP11-6O2.2 |
| hsa-miR-186-5p | RP11-2E11.5 | hsa-miR-146b-5p | RP11-831F12.2 | hsa-miR-140-3p | RP11-6O2.2 |
| hsa-miR-146b-5p | RP11-2E11.5 | hsa-miR-186-5p | RP11-382A20.6 | hsa-miR-143-3p | MED4-AS1 |
| hsa-miR-378a-3p | RP11-659P15.1 | hsa-miR-186-5p | LINC01018 | hsa-miR-34a-5p | MED4-AS1 |
| hsa-miR-186-5p | RP11-659P15.1 | hsa-miR-34a-5p | FENDRR | hsa-miR-34a-5p | LINC01272 |
| hsa-miR-378h | RP11-659P15.1 | hsa-miR-186-5p | FENDRR | hsa-miR-143-3p | LINC01272 |
| hsa-miR-499a-5p | RP11-317G6.1 | hsa-miR-25-3p | RP11-346C20.4 | hsa-miR-25-3p | RP11-767I20.1 |
| hsa-miR-143-3p | PIK3IP1-AS1 | hsa-miR-186-5p | RP13-631K18.3 | hsa-miR-140-3p | RP11-767I20.1 |
| hsa-miR-25-3p | RP11-834C11.6 | hsa-miR-148a-3p | AC005740.6 | hsa-miR-143-3p | RP11-192H23.7 |
| hsa-miR-26b-5p | RP11-521O16.2 | hsa-miR-148b-3p | AC005740.6 | hsa-miR-146b-5p | RP11-192H23.7 |
| hsa-miR-27b-3p | RP11-521O16.2 | hsa-miR-27b-3p | AC005740.6 | hsa-miR-27b-3p | LINC01266 |
| hsa-miR-25-3p | RP11-521O16.2 | hsa-miR-199a-5p | RP11-367G6.3 | hsa-miR-186-5p | LINC01266 |
| hsa-miR-125b-5p | RP11-597D13.9 | hsa-miR-34a-5p | RP3-431P23.5 | hsa-miR-148b-3p | LINC01266 |
| hsa-miR-374b-5p | RP11-597D13.9 | hsa-miR-125b-5p | LINC01311 | hsa-miR-27b-3p | RP11-332J15.4 |
| hsa-miR-146b-5p | LINC01559 | hsa-miR-27b-3p | LINC01311 | hsa-miR-140-3p | RP11-332J15.4 |
| hsa-miR-26b-5p | LINC01559 | hsa-miR-499a-5p | RP11-159D12.2 | hsa-miR-221-3p | CTD-2510F5.4 |
| hsa-miR-27b-3p | AC010729.2 | hsa-miR-199a-5p | RP11-159D12.2 | hsa-miR-186-5p | RP1-142L7.9 |
| hsa-miR-125b-5p | CELF2-AS1 | hsa-miR-143-3p | LINC00152 | hsa-miR-421 | RP11-10C24.3 |
| hsa-miR-34a-5p | AC093382.1 | hsa-miR-27b-3p | RP11-492E3.2 | hsa-miR-186-5p | RP11-116O18.1 |
| hsa-miR-186-5p | RP11-268G12.3 | hsa-miR-26b-5p | RP11-492E3.2 | hsa-miR-146b-5p | RP11-116O18.1 |
| hsa-miR-125b-5p | RP11-268G12.3 | hsa-miR-140-3p | RP4-646N3.1 | hsa-miR-146b-5p | CTD-2583A14.8 |
| hsa-miR-27b-3p | TSPEAR-AS2 | hsa-miR-221-3p | RP4-646N3.1 | hsa-miR-186-5p | RP11-583F2.2 |
| hsa-miR-148a-3p | TSPEAR-AS2 | hsa-miR-143-3p | RP11-338I21.1 | hsa-miR-140-3p | RP11-583F2.2 |

| | | | | | |
|---|---|---|---|---|---|
| hsa-miR-199a-5p | RP1-154K9.2 | hsa-miR-143-3p | RP11-2L8.1 | hsa-miR-374b-5p | RP11-583F2.2 |
| hsa-miR-26b-5p | RP1-154K9.2 | hsa-miR-143-3p | RP1-224A6.9 | hsa-miR-148a-3p | LINC01265 |
| hsa-miR-34a-5p | RP11-408A13.3 | hsa-miR-186-5p | RP11-305K5.1 | hsa-miR-499a-5p | LINC00908 |
| hsa-miR-378h | RP11-443B7.1 | hsa-miR-374b-5p | RP11-902B17.1 | hsa-miR-221-3p | LINC00908 |
| hsa-miR-378a-3p | RP11-443B7.1 | hsa-miR-34a-5p | GAS8-AS1 | hsa-miR-146b-5p | RP11-325K4.3 |
| hsa-miR-27b-3p | RP11-443B7.1 | hsa-miR-125b-5p | RP11-314M24.1 | hsa-miR-499a-5p | SNHG3 |
| hsa-miR-221-3p | RP11-624D11.2 | hsa-miR-186-5p | RP11-314M24.1 | hsa-miR-146b-5p | SNHG3 |
| hsa-miR-199a-5p | CTC-367F4.1 | hsa-miR-421 | RP3-508I15.20 | hsa-miR-421 | RP11-286H15.1 |
| hsa-miR-199a-5p | RP11-54O7.18 | hsa-miR-125b-5p | RP11-672A2.1 | hsa-miR-125b-5p | AC018742.1 |
| hsa-miR-25-3p | AC098617.1 | hsa-miR-148a-3p | CTD-2555A7.3 | hsa-miR-143-3p | RP11-1084E5.1 |
| hsa-miR-125b-5p | AC098617.1 | hsa-miR-148b-3p | CTD-2555A7.3 | hsa-miR-421 | RP11-400N13.3 |
| hsa-miR-26b-5p | RP13-494C23.1 | hsa-miR-26b-5p | RP11-391H12.8 | hsa-miR-140-3p | RP11-389G6.3 |
| hsa-miR-378a-3p | RP4-625H18.2 | hsa-miR-125b-5p | RP11-95O2.1 | hsa-miR-148a-3p | RP1-102E24.8 |
| hsa-miR-378h | RP4-625H18.2 | hsa-miR-499a-5p | RP11-861E21.2 | hsa-miR-34a-5p | ETV5-AS1 |
| hsa-miR-27b-3p | CASC11 | hsa-miR-27b-3p | RP11-861E21.2 | hsa-miR-34a-5p | RP11-443B7.3 |
| hsa-miR-143-3p | CASC11 | hsa-miR-186-5p | MGC15885 | hsa-miR-186-5p | RP11-443B7.3 |
| hsa-miR-125b-5p | KCNQ1OT1 | hsa-miR-34a-5p | MGC15885 | hsa-miR-421 | KB-68A7.1 |
| hsa-miR-27b-3p | KCNQ1OT1 | hsa-miR-143-3p | RP11-820I16.1 | hsa-miR-143-3p | RP11-348J24.2 |
| hsa-miR-143-3p | KCNQ1OT1 | hsa-miR-125b-5p | FTO-IT1 | hsa-miR-378a-3p | HS1BP3-IT1 |
| hsa-miR-34a-5p | KCNQ1OT1 | hsa-miR-125b-5p | HOXB-AS3 | hsa-miR-378h | HS1BP3-IT1 |
| hsa-miR-26b-5p | KCNQ1OT1 | hsa-miR-378a-3p | HOXB-AS3 | hsa-miR-148b-3p | CTD-2171N6.1 |
| hsa-miR-374b-5p | KCNQ1OT1 | hsa-miR-378h | HOXB-AS3 | hsa-miR-148a-3p | CTD-2171N6.1 |
| hsa-miR-140-3p | KCNQ1OT1 | hsa-miR-186-5p | RP11-19E18.2 | hsa-miR-186-5p | RP11-290F5.1 |
| hsa-miR-199a-5p | KCNQ1OT1 | hsa-miR-27b-3p | FAM215B | hsa-miR-143-3p | RP11-290F5.1 |
| hsa-miR-146b-5p | KCNQ1OT1 | hsa-miR-27b-3p | RP11-673E1.1 | hsa-miR-199a-5p | RP11-423G4.10 |
| hsa-miR-378h | KCNQ1OT1 | hsa-miR-421 | RP11-673E1.1 | hsa-miR-221-3p | RP11-423G4.10 |
| hsa-miR-148a-3p | KCNQ1OT1 | hsa-miR-26b-5p | RP11-673E1.1 | hsa-miR-378h | RP11-314O13.1 |
| hsa-miR-378a-3p | KCNQ1OT1 | hsa-miR-140-3p | RP11-673E1.1 | hsa-miR-378a-3p | RP11-314O13.1 |

| | | | | | |
|---|---|---|---|---|---|
| hsa-miR-421 | KCNQ1OT1 | hsa-miR-378a-3p | RP11-673E1.1 | hsa-miR-125b-5p | RP11-314O13.1 |
| hsa-miR-148b-3p | KCNQ1OT1 | hsa-miR-125b-5p | RP11-673E1.1 | hsa-miR-125b-5p | RP11-455F5.6 |
| hsa-miR-25-3p | KCNQ1OT1 | hsa-miR-374b-5p | RP11-673E1.1 | hsa-miR-421 | RP11-455F5.6 |
| hsa-miR-499a-5p | KCNQ1OT1 | hsa-miR-186-5p | chr22-38_28785274-29006793.1 | hsa-miR-140-3p | RP3-340N1.2 |
| hsa-miR-186-5p | KCNQ1OT1 | hsa-miR-143-3p | chr22-38_28785274-29006793.1 | hsa-miR-34a-5p | RP3-340N1.2 |
| hsa-miR-221-3p | KCNQ1OT1 | hsa-miR-146b-5p | chr22-38_28785274-29006793.1 | hsa-miR-143-3p | RP3-340N1.2 |
| hsa-miR-499a-5p | LINC00426 | hsa-miR-27b-3p | chr22-38_28785274-29006793.1 | hsa-miR-186-5p | AC007326.10 |
| hsa-miR-199a-5p | LINC00426 | hsa-miR-140-3p | chr22-38_28785274-29006793.1 | hsa-miR-148a-3p | AC007326.10 |
| hsa-miR-27b-3p | LINC00426 | hsa-miR-125b-5p | chr22-38_28785274-29006793.1 | hsa-miR-148b-3p | AC007326.10 |
| hsa-miR-143-3p | CASC2 | hsa-miR-26b-5p | chr22-38_28785274-29006793.1 | hsa-miR-27b-3p | AC007326.10 |
| hsa-miR-499a-5p | CASC2 | hsa-miR-374b-5p | chr22-38_28785274-29006793.1 | hsa-miR-27b-3p | RP11-473O4.4 |

| | | | | | |
|---|---|---|---|---|---|
| hsa-miR-186-5p | CASC2 | hsa-miR-34a-5p | chr22-38_28785274-29006793.1 | hsa-miR-125b-5p | RP11-473O4.4 |
| hsa-miR-125b-5p | CASC2 | hsa-miR-499a-5p | chr22-38_28785274-29006793.1 | hsa-miR-125b-5p | RP11-284F21.9 |
| hsa-miR-199a-5p | AC007161.5 | hsa-miR-148a-3p | chr22-38_28785274-29006793.1 | hsa-miR-378h | RP11-807H22.7 |
| hsa-miR-186-5p | RP1-5O6.6 | hsa-miR-378h | chr22-38_28785274-29006793.1 | hsa-miR-378a-3p | RP11-807H22.7 |
| hsa-miR-148a-3p | RP11-256L6.2 | hsa-miR-148b-3p | chr22-38_28785274-29006793.1 | hsa-miR-421 | LINC00664 |
| hsa-miR-374b-5p | RP11-438L19.1 | hsa-miR-378a-3p | chr22-38_28785274-29006793.1 | hsa-miR-199a-5p | LINC00664 |
| hsa-miR-146b-5p | RP11-254I22.1 | hsa-miR-199a-5p | chr22-38_28785274-29006793.1 | hsa-miR-125b-5p | LINC00664 |
| hsa-miR-186-5p | RP11-272L14.2 | hsa-miR-421 | chr22-38_28785274-29006793.1 | hsa-miR-27b-3p | LINC00664 |
| hsa-miR-27b-3p | AC012506.4 | hsa-miR-25-3p | chr22-38_28785274-29006793.1 | hsa-miR-26b-5p | RP11-427L15.2 |

| | | | | | |
|---|---|---|---|---|---|
| hsa-miR-186-5p | RP11-45M22.5 | hsa-miR-221-3p | chr22-38_28785274-29006793.1 | hsa-miR-125b-5p | RP11-111I12.1 |
| hsa-miR-27b-3p | RP11-462L8.1 | hsa-miR-148b-3p | RP11-231N3.1 | hsa-miR-499a-5p | AC097467.2 |
| hsa-miR-499a-5p | RP11-462L8.1 | hsa-miR-148a-3p | RP11-231N3.1 | hsa-miR-27b-3p | AC097467.2 |
| hsa-miR-143-3p | DGCR9 | hsa-miR-374b-5p | LINC01362 | hsa-miR-378h | AC097467.2 |
| hsa-miR-199a-5p | ROR1-AS1 | hsa-miR-143-3p | RP11-536K7.3 | hsa-miR-378a-3p | AC097467.2 |
| hsa-miR-186-5p | LINC00472 | hsa-miR-143-3p | LINC00216 | hsa-miR-27b-3p | RP11-27G22.1 |
| hsa-miR-374b-5p | RP11-403A21.1 | hsa-miR-499a-5p | LINC00216 | hsa-miR-26b-5p | RP11-439C15.4 |
| hsa-miR-199a-5p | RP11-267N12.1 | hsa-miR-421 | RP11-314C16.1 | hsa-miR-499a-5p | XXyac-YX155B6.5 |
| hsa-miR-186-5p | GDNF-AS1 | hsa-miR-221-3p | RP11-326A19.3 | hsa-miR-146b-5p | XXyac-YX155B6.5 |
| hsa-miR-421 | AC007126.1 | hsa-miR-146b-5p | AC073115.7 | hsa-miR-186-5p | RP11-64D24.4 |
| hsa-miR-27b-3p | RP11-875O11.3 | hsa-miR-186-5p | RP11-89N17.4 | hsa-miR-125b-5p | RP11-64D24.4 |
| hsa-miR-125b-5p | RP11-875O11.3 | hsa-miR-27b-3p | XXbac-BPG248L24.13 | hsa-miR-148b-3p | RP11-64D24.4 |
| hsa-miR-499a-5p | RP11-497G19.7 | hsa-miR-34a-5p | RP11-394J1.2 | hsa-miR-148a-3p | RP11-64D24.4 |
| hsa-miR-378h | LINC01436 | hsa-miR-143-3p | RP11-394I13.2 | hsa-miR-378h | ACTA2-AS1 |
| hsa-miR-146b-5p | RP11-238K6.1 | hsa-miR-374b-5p | RP11-505E24.3 | hsa-miR-378a-3p | ACTA2-AS1 |
| hsa-miR-186-5p | RP11-238K6.1 | hsa-miR-143-3p | RP5-839B4.8 | hsa-miR-143-3p | ACTA2-AS1 |
| hsa-miR-146b-5p | CTC-338M12.6 | hsa-miR-146b-5p | RP5-839B4.8 | hsa-miR-27b-3p | LINC01355 |
| hsa-miR-199a-5p | AC006262.4 | hsa-miR-34a-5p | CTD-2527I21.15 | hsa-miR-186-5p | LINC01355 |
| hsa-miR-499a-5p | RP11-468H14.2 | hsa-miR-148b-3p | PTCSC3 | hsa-miR-186-5p | AC012593.1 |
| hsa-miR-34a-5p | RP11-642D21.1 | hsa-miR-148a-3p | PTCSC3 | hsa-miR-146b-5p | AC012593.1 |
| hsa-miR-146b-5p | SC22CB-109E1.1 | hsa-miR-499a-5p | RP5-991G20.6 | hsa-miR-27b-3p | RP11-316M20.1 |
| hsa-miR-26b-5p | SC22CB-109E1.1 | hsa-miR-27b-3p | RP11-181B11.1 | hsa-miR-34a-5p | LINC00906 |
| hsa-miR-143-3p | SC22CB-109E1.1 | hsa-miR-143-3p | CTC-498M16.2 | hsa-miR-27b-3p | RP11-1008C21.1 |

| | | | | | |
|---|---|---|---|---|---|
| hsa-miR-421 | SC22CB-109E1.1 | hsa-miR-186-5p | LINC01315 | hsa-miR-26b-5p | RP11-1008C21.1 |
| hsa-miR-125b-5p | RP1-60O19.1 | hsa-miR-143-3p | INHBA-AS1 | hsa-miR-140-3p | RP11-1008C21.1 |
| hsa-miR-146b-5p | RP1-60O19.1 | hsa-miR-374b-5p | INHBA-AS1 | hsa-miR-146b-5p | RP11-285E9.5 |
| hsa-miR-34a-5p | RP11-451B8.1 | hsa-miR-374b-5p | RP5-998N21.4 | hsa-miR-25-3p | LINC00533 |
| hsa-miR-374b-5p | RP11-210K20.5 | hsa-miR-34a-5p | RP5-998N21.4 | hsa-miR-186-5p | LINC00304 |
| hsa-miR-146b-5p | RP11-210K20.5 | hsa-miR-148a-3p | RP11-381O6.1 | hsa-miR-148b-3p | AP001092.4 |
| hsa-miR-186-5p | XXbac-B476C20.13 | hsa-miR-27b-3p | RP11-44F14.8 | hsa-miR-148a-3p | AP001092.4 |
| hsa-miR-34a-5p | XXbac-B476C20.13 | hsa-miR-186-5p | RP11-44F14.8 | hsa-miR-143-3p | AC007038.7 |
| hsa-miR-146b-5p | RP11-121A8.1 | hsa-miR-143-3p | RP11-44F14.8 | hsa-miR-34a-5p | LINC01359 |
| hsa-miR-140-3p | RP11-121A8.1 | hsa-miR-146b-5p | RP11-44F14.8 | hsa-miR-221-3p | LINC01359 |
| hsa-miR-221-3p | RP11-867G23.3 | hsa-miR-499a-5p | RP11-44F14.8 | hsa-miR-27b-3p | RP1-287H17.1 |
| hsa-miR-27b-3p | RP11-76I14.1 | hsa-miR-374b-5p | RP11-44F14.8 | hsa-miR-143-3p | LINC00865 |
| hsa-miR-34a-5p | RP11-76I14.1 | hsa-miR-125b-5p | RP11-44F14.8 | hsa-miR-146b-5p | LINC00865 |
| hsa-miR-26b-5p | RP11-76I14.1 | hsa-miR-186-5p | LLNLR-246C6.1 | hsa-miR-25-3p | CTD-2562J15.6 |
| hsa-miR-34a-5p | RP11-102N12.3 | hsa-miR-27b-3p | LLNLR-246C6.1 | hsa-miR-143-3p | CTC-265F19.1 |
| hsa-miR-140-3p | RP11-336K24.12 | hsa-miR-421 | LLNLR-246C6.1 | hsa-miR-499a-5p | LINC01235 |
| hsa-miR-125b-5p | RP11-138P22.1 | hsa-miR-140-3p | AP004372.1 | hsa-miR-221-3p | LINC01235 |
| hsa-miR-221-3p | RP11-567J20.3 | hsa-miR-374b-5p | RP11-231E19.1 | hsa-miR-27b-3p | RP11-588H23.3 |
| hsa-miR-199a-5p | CTD-3216D2.5 | hsa-miR-140-3p | RP11-231E19.1 | hsa-miR-27b-3p | CTD-2538C1.3 |
| hsa-miR-146b-5p | CTD-3216D2.5 | hsa-miR-25-3p | LINC01021 | hsa-miR-186-5p | AC010095.5 |
| hsa-miR-186-5p | RP11-9E17.1 | hsa-miR-186-5p | LINC01021 | hsa-miR-143-3p | AC024084.1 |
| hsa-miR-374b-5p | RP11-346C4.3 | hsa-miR-25-3p | RP11-92B11.3 | hsa-miR-143-3p | AL133493.2 |
| hsa-miR-374b-5p | RP11-629G13.1 | hsa-miR-27b-3p | RP11-92B11.3 | hsa-miR-34a-5p | RP11-65L3.2 |
| hsa-miR-125b-5p | RP11-352D13.5 | hsa-miR-125b-5p | RP4-669P10.16 | hsa-miR-186-5p | RP11-65L3.2 |
| hsa-miR-27b-3p | RP11-91J3.3 | hsa-miR-378a-3p | RP11-680F20.10 | hsa-miR-199a-5p | RP11-65L3.2 |
| hsa-miR-143-3p | CTD-2571L23.8 | hsa-miR-378h | RP11-680F20.10 | hsa-miR-125b-5p | LINC01426 |

| | | | | | |
|---|---|---|---|---|---|
| hsa-miR-25-3p | CTD-2571L23.8 | hsa-miR-146b-5p | RP11-680F20.10 | hsa-miR-143-3p | LINC01426 |
| hsa-miR-146b-5p | RP4-655J12.4 | hsa-miR-140-3p | RP11-680F20.10 | hsa-miR-186-5p | MIR155HG |
| hsa-miR-27b-3p | RP11-81H14.2 | hsa-miR-374b-5p | RP11-680F20.10 | hsa-miR-140-3p | MIR155HG |
| hsa-miR-27b-3p | AP000221.1 | hsa-miR-421 | AC002044.4 | hsa-miR-499a-5p | RP11-359E10.1 |
| hsa-miR-378a-3p | AC007461.2 | hsa-miR-27b-3p | AC017002.2 | hsa-miR-143-3p | SIRPG-AS1 |
| hsa-miR-378h | AC007461.2 | hsa-miR-421 | RP11-385N23.1 | hsa-miR-143-3p | CTB-171A8.1 |
| hsa-miR-143-3p | AC007461.2 | hsa-miR-199a-5p | AC097495.3 | hsa-miR-143-3p | AC108676.1 |
| hsa-miR-34a-5p | AC007461.2 | hsa-miR-25-3p | AC097495.3 | hsa-miR-143-3p | Z83844.1 |
| hsa-miR-27b-3p | RP11-710F7.3 | hsa-miR-186-5p | RP3-333A15.1 | hsa-miR-140-3p | RP11-528A4.2 |
| hsa-miR-421 | RP11-283C24.1 | hsa-miR-499a-5p | RP3-333A15.1 | hsa-miR-143-3p | CDKN2B-AS1 |
| hsa-miR-148a-3p | RP11-540A21.2 | hsa-miR-499a-5p | AC069363.1 | hsa-miR-140-3p | CDKN2B-AS1 |
| hsa-miR-148b-3p | RP11-540A21.2 | hsa-miR-27b-3p | AC069363.1 | hsa-miR-148a-3p | CDKN2B-AS1 |
| hsa-miR-186-5p | RP11-540A21.2 | hsa-miR-146b-5p | AC069363.1 | hsa-miR-148b-3p | CDKN2B-AS1 |
| hsa-miR-34a-5p | C1QTNF1-AS1 | hsa-miR-146b-5p | PCAT18 | hsa-miR-125b-5p | AP000472.2 |
| hsa-miR-186-5p | CLYBL-AS2 | hsa-miR-34a-5p | RP11-17G12.2 | hsa-miR-143-3p | AP000472.2 |
| hsa-miR-26b-5p | RP11-679B19.1 | hsa-miR-146b-5p | RP11-417B4.2 | hsa-miR-27b-3p | AP000344.3 |
| hsa-miR-146b-5p | RP1-155D22.2 | hsa-miR-34a-5p | RP11-417B4.2 | hsa-miR-186-5p | PVT1 |
| hsa-miR-125b-5p | CTD-2353F22.1 | hsa-miR-143-3p | RP11-417B4.2 | hsa-miR-140-3p | PVT1 |
| hsa-miR-148b-3p | SNHG24 | hsa-miR-140-3p | RP1-197B17.5 | hsa-miR-374b-5p | PVT1 |
| hsa-miR-148b-3p | RBMS3-AS2 | hsa-miR-186-5p | CTB-114C7.4 | hsa-miR-148b-3p | PVT1 |
| hsa-miR-27b-3p | LINC00880 | hsa-miR-374b-5p | CTB-114C7.4 | hsa-miR-186-5p | AF011889.2 |
| hsa-miR-499a-5p | RP11-362F19.3 | hsa-miR-143-3p | RP5-1014D13.2 | hsa-miR-378a-3p | RP4-647J21.1 |
| hsa-miR-143-3p | RP11-212I21.2 | hsa-miR-25-3p | RP5-1014D13.2 | hsa-miR-378h | RP4-647J21.1 |
| hsa-miR-27b-3p | RP11-212I21.2 | hsa-miR-26b-5p | RP5-1014D13.2 | hsa-miR-26b-5p | LINC00174 |
| hsa-miR-186-5p | LINC00589 | hsa-miR-186-5p | RP5-1014D13.2 | hsa-miR-378h | LINC00174 |
| hsa-miR-143-3p | LINC00589 | hsa-miR-199a-5p | RP5-1014D13.2 | hsa-miR-378a-3p | LINC00174 |
| hsa-miR-27b-3p | RP11-383H13.1 | hsa-miR-27b-3p | RP11-96O20.5 | hsa-miR-421 | LINC00174 |
| hsa-miR-186-5p | RP11-383H13.1 | hsa-miR-27b-3p | OSTM1-AS1 | hsa-miR-143-3p | LINC00174 |

| | | | | | |
|---|---|---|---|---|---|
| hsa-miR-34a-5p | RP11-383H13.1 | hsa-miR-125b-5p | RP11-661A12.9 | hsa-miR-148b-3p | LINC00174 |
| hsa-miR-374b-5p | RP1-102K2.8 | hsa-miR-34a-5p | RP11-1069G10.1 | hsa-miR-25-3p | LINC00174 |
| hsa-miR-186-5p | RP11-13K12.2 | hsa-miR-143-3p | RP11-1069G10.1 | hsa-miR-148a-3p | RP11-489G11.3 |
| hsa-miR-374b-5p | RP11-81H14.1 | hsa-miR-378h | RP11-434H6.6 | hsa-miR-148b-3p | RP11-489G11.3 |
| hsa-miR-143-3p | RP5-1120P11.3 | hsa-miR-140-3p | RP11-434H6.6 | hsa-miR-125b-5p | RP11-431J24.2 |
| hsa-miR-125b-5p | RP4-671O14.7 | hsa-miR-378a-3p | RP11-434H6.6 | hsa-miR-421 | RP11-431J24.2 |
| hsa-miR-27b-3p | RP4-671O14.7 | hsa-miR-143-3p | RP3-412A9.17 | hsa-miR-186-5p | RP1-102K2.6 |
| hsa-miR-421 | RP4-671O14.7 | hsa-miR-199a-5p | RP3-412A9.17 | hsa-miR-27b-3p | RP11-508N22.13 |
| hsa-miR-143-3p | RP4-671O14.7 | hsa-miR-26b-5p | RP3-412A9.17 | hsa-miR-143-3p | CYP51A1-AS1 |
| hsa-miR-148b-3p | RP4-671O14.7 | hsa-miR-27b-3p | RP3-412A9.17 | hsa-miR-146b-5p | MIR222HG |
| hsa-miR-221-3p | RP4-671O14.7 | hsa-miR-148b-3p | RP3-412A9.17 | hsa-miR-125b-5p | RP1-79C4.4 |
| hsa-miR-148a-3p | RP4-671O14.7 | hsa-miR-148a-3p | RP3-412A9.17 | hsa-miR-27b-3p | RP1-79C4.4 |
| hsa-miR-186-5p | RP4-671O14.7 | hsa-miR-186-5p | RP3-412A9.17 | hsa-miR-421 | IBA57-AS1 |
| hsa-miR-26b-5p | RP4-671O14.7 | hsa-miR-25-3p | RP3-412A9.17 | hsa-miR-199a-5p | RP4-580N22.2 |
| hsa-miR-374b-5p | RP4-671O14.7 | hsa-miR-378h | RP3-412A9.17 | hsa-miR-186-5p | RP11-359E8.5 |
| hsa-miR-421 | RP1-29C18.8 | hsa-miR-378a-3p | RP3-412A9.17 | hsa-miR-25-3p | RP11-359E8.5 |
| hsa-miR-421 | RP11-452H21.4 | hsa-miR-499a-5p | RP3-412A9.17 | hsa-miR-186-5p | LINC01440 |
| hsa-miR-143-3p | RP11-452H21.4 | hsa-miR-374b-5p | AC002117.1 | hsa-miR-26b-5p | RP4-781K5.6 |
| hsa-miR-34a-5p | RP11-331F9.3 | hsa-miR-125b-5p | RP11-517C16.2 | hsa-miR-34a-5p | RP11-982M15.6 |
| hsa-miR-34a-5p | RP11-511B23.2 | hsa-miR-125b-5p | MIRLET7BHG | hsa-miR-140-3p | RP11-426C22.5 |
| hsa-miR-27b-3p | FGF14-IT1 | hsa-miR-146b-5p | RP11-982M15.7 | hsa-miR-374b-5p | RP11-30P6.6 |
| hsa-miR-125b-5p | RP11-261P9.4 | hsa-miR-143-3p | C20orf203 | hsa-miR-146b-5p | RP11-30P6.6 |
| hsa-miR-499a-5p | PRICKLE2-AS1 | hsa-miR-146b-5p | RAP2C-AS1 | hsa-miR-140-3p | RP11-30P6.6 |
| hsa-miR-140-3p | PRICKLE2-AS1 | hsa-miR-186-5p | RAP2C-AS1 | hsa-miR-146b-5p | RP11-20B24.5 |
| hsa-miR-27b-3p | RP3-368B9.2 | hsa-miR-125b-5p | RP11-91P24.6 | hsa-miR-146b-5p | RP11-149I23.3 |
| hsa-miR-34a-5p | RP3-368B9.2 | hsa-miR-27b-3p | RP11-158H5.8 | hsa-miR-148b-3p | HOXA11-AS |
| hsa-miR-378a-3p | AC116366.6 | hsa-miR-26b-5p | RP5-1112D6.7 | hsa-miR-146b-5p | HOXA11-AS |
| hsa-miR-378h | AC116366.6 | hsa-miR-34a-5p | GS1-600G8.5 | hsa-miR-148a-3p | HOXA11-AS |

| | | | | | |
|---|---|---|---|---|---|
| hsa-miR-25-3p | AC116366.6 | hsa-miR-125b-5p | RP11-380J14.1 | hsa-miR-27b-3p | AP000233.4 |
| hsa-miR-125b-5p | RP11-371F15.3 | hsa-miR-199a-5p | RP11-468E2.5 | hsa-miR-26b-5p | RP11-547D24.1 |
| hsa-miR-26b-5p | RP4-761J14.8 | hsa-miR-34a-5p | RP3-322G13.7 | hsa-miR-27b-3p | FAM225B |
| hsa-miR-378h | RP4-761J14.8 | hsa-miR-140-3p | RP3-323N1.2 | hsa-miR-34a-5p | RP4-782L23.2 |
| hsa-miR-378a-3p | RP4-761J14.8 | hsa-miR-143-3p | RP11-63A11.1 | hsa-miR-125b-5p | RP4-782L23.2 |
| hsa-miR-199a-5p | RP4-761J14.8 | hsa-miR-374b-5p | RP11-584P21.2 | hsa-miR-374b-5p | RP11-999E24.3 |
| hsa-miR-146b-5p | AC006272.2 | hsa-miR-25-3p | RP11-584P21.2 | hsa-miR-26b-5p | CTD-2537I9.13 |
| hsa-miR-125b-5p | AC006272.2 | hsa-miR-143-3p | CTD-3020H12.4 | hsa-miR-27b-3p | AF064858.8 |
| hsa-miR-186-5p | AC006272.2 | hsa-miR-186-5p | RP11-13J8.1 | hsa-miR-27b-3p | RP11-66N24.6 |
| hsa-miR-34a-5p | RP11-727F15.9 | hsa-miR-27b-3p | FAM225A | hsa-miR-374b-5p | RP11-571I18.5 |
| hsa-miR-140-3p | XXbac-BPG157A10.21 | hsa-miR-199a-5p | RP1-27K12.2 | hsa-miR-27b-3p | RP11-571I18.5 |
| hsa-miR-186-5p | WT1-AS | hsa-miR-378h | RP11-467L24.1 | hsa-miR-146b-5p | RP11-575F12.1 |
| hsa-miR-34a-5p | WT1-AS | hsa-miR-378a-3p | RP11-467L24.1 | hsa-miR-146b-5p | RP11-92C4.3 |
| hsa-miR-374b-5p | WT1-AS | hsa-miR-143-3p | RP11-467L24.1 | hsa-miR-421 | RP11-322E11.5 |
| hsa-miR-186-5p | CTD-2587M2.1 | hsa-miR-27b-3p | CTD-2562J15.4 | hsa-miR-27b-3p | RP11-322E11.5 |
| hsa-miR-148b-3p | AC007362.3 | hsa-miR-34a-5p | AC017101.10 | hsa-miR-140-3p | ERVH48-1 |
| hsa-miR-186-5p | RP11-680G24.6 | hsa-miR-125b-5p | CTA-221G9.7 | hsa-miR-499a-5p | ERVH48-1 |
| hsa-miR-143-3p | CTB-181H17.1 | hsa-miR-374b-5p | LINC00240 | hsa-miR-27b-3p | ERVH48-1 |
| hsa-miR-125b-5p | LINC00565 | hsa-miR-146b-5p | LINC00240 | hsa-miR-374b-5p | ERVH48-1 |
| hsa-miR-26b-5p | LINC00565 | hsa-miR-499a-5p | LINC00240 | hsa-miR-34a-5p | ERVH48-1 |
| hsa-miR-143-3p | GABPB1-AS1 | hsa-miR-125b-5p | RP11-481J2.2 | hsa-miR-421 | ERVH48-1 |
| hsa-miR-186-5p | GABPB1-AS1 | hsa-miR-378h | RP11-481J2.2 | hsa-miR-186-5p | ERVH48-1 |
| hsa-miR-499a-5p | GABPB1-AS1 | hsa-miR-378a-3p | RP11-481J2.2 | hsa-miR-125b-5p | AP001046.6 |
| hsa-miR-374b-5p | GABPB1-AS1 | hsa-miR-125b-5p | AATBC | hsa-miR-34a-5p | RP11-3P17.5 |
| hsa-miR-421 | GABPB1-AS1 | hsa-miR-27b-3p | AATBC | hsa-miR-26b-5p | CTB-107G13.1 |
| hsa-miR-26b-5p | GABPB1-AS1 | hsa-miR-186-5p | RP11-1M18.1 | hsa-miR-378a-3p | CTD-2530N21.5 |
| hsa-miR-199a-5p | RP11-61F12.1 | hsa-miR-186-5p | TTLL7-IT1 | hsa-miR-378h | CTD-2530N21.5 |

| | | | | | |
|---|---|---|---|---|---|
| hsa-miR-499a-5p | LINC00535 | hsa-miR-125b-5p | RP4-798A10.4 | hsa-miR-148b-3p | AP000282.3 |
| hsa-miR-199a-5p | LINC00535 | hsa-miR-27b-3p | CTB-178M22.1 | hsa-miR-148a-3p | AP000282.3 |
| hsa-miR-143-3p | RP11-525A16.4 | hsa-miR-186-5p | RP11-526I2.5 | hsa-miR-499a-5p | RP5-921G16.2 |
| hsa-miR-27b-3p | RP11-293M10.6 | hsa-miR-27b-3p | RP11-526I2.5 | hsa-miR-143-3p | CH17-264L24.1 |
| hsa-miR-140-3p | LINC01234 | hsa-miR-421 | RP11-526I2.5 | hsa-miR-199a-5p | CH17-264L24.1 |
| hsa-miR-27b-3p | LINC01543 | hsa-miR-199a-5p | RP11-526I2.5 | hsa-miR-125b-5p | CH17-264L24.1 |
| hsa-miR-146b-5p | BTBD9-AS1 | hsa-miR-146b-5p | AC009303.1 | hsa-miR-374b-5p | CH17-264L24.1 |
| hsa-miR-199a-5p | BTBD9-AS1 | hsa-miR-186-5p | ARAP1-AS2 | hsa-miR-27b-3p | RP11-753H16.5 |
| hsa-miR-27b-3p | LINC00608 | hsa-miR-146b-5p | RP11-265N7.1 | hsa-miR-125b-5p | FLJ45079 |
| hsa-miR-421 | RP3-492J12.2 | hsa-miR-186-5p | RP11-265N7.1 | hsa-miR-199a-5p | FLJ45079 |
| hsa-miR-25-3p | LINC00365 | hsa-miR-148a-3p | RP11-265N7.1 | hsa-miR-186-5p | RP11-154H23.3 |
| hsa-miR-34a-5p | LINC00365 | hsa-miR-143-3p | RP11-436K8.1 | hsa-miR-34a-5p | RP11-154H23.3 |
| hsa-miR-186-5p | RP1-140K8.5 | hsa-miR-34a-5p | RP11-436K8.1 | hsa-miR-34a-5p | RP11-669E14.6 |
| hsa-miR-186-5p | RP11-723O4.9 | hsa-miR-27b-3p | CARS-AS1 | hsa-miR-186-5p | RP11-504A18.1 |
| hsa-miR-378h | RP11-723O4.9 | hsa-miR-26b-5p | RP11-245P10.6 | hsa-miR-499a-5p | RP11-504A18.1 |
| hsa-miR-378a-3p | RP11-723O4.9 | hsa-miR-378a-3p | LINC01133 | hsa-miR-143-3p | RP11-525K10.3 |
| hsa-miR-148b-3p | AC104820.2 | hsa-miR-378h | LINC01133 | hsa-miR-143-3p | LCMT1-AS1 |
| hsa-miR-143-3p | RP11-794M8.1 | hsa-miR-186-5p | RP11-403I13.5 | hsa-miR-186-5p | LCMT1-AS1 |
| hsa-miR-199a-5p | RP11-298D21.1 | hsa-miR-421 | LINC01433 | hsa-miR-27b-3p | LCMT1-AS1 |
| hsa-miR-143-3p | AC004231.2 | hsa-miR-140-3p | LINC00632 | hsa-miR-199a-5p | AP003774.1 |
| hsa-miR-146b-5p | CTC-251D13.1 | hsa-miR-27b-3p | AC144831.1 | hsa-miR-34a-5p | AC007879.1 |
| hsa-miR-140-3p | RP11-423P10.2 | hsa-miR-125b-5p | PRKG1-AS1 | hsa-miR-186-5p | RP11-315O6.1 |
| hsa-miR-186-5p | CFLAR-AS1 | hsa-miR-27b-3p | MIMT1 | hsa-miR-499a-5p | RP11-92F20.1 |
| hsa-miR-146b-5p | CFLAR-AS1 | hsa-miR-143-3p | MIMT1 | hsa-miR-27b-3p | RP11-562A8.4 |
| hsa-miR-143-3p | CFLAR-AS1 | hsa-miR-421 | RP11-1105G2.4 | hsa-miR-27b-3p | CTD-2659N19.9 |
| hsa-miR-125b-5p | CFLAR-AS1 | hsa-miR-34a-5p | AC012668.2 | hsa-miR-146b-5p | WI2-85898F10.2 |
| hsa-miR-27b-3p | RP11-152H18.4 | hsa-miR-221-3p | LINC00158 | hsa-miR-378a-3p | RP11-322E11.2 |
| hsa-miR-25-3p | AC016700.2 | hsa-miR-143-3p | LINC00158 | hsa-miR-378h | RP11-322E11.2 |

| | | | | | |
|---|---|---|---|---|---|
| hsa-miR-143-3p | AC016644.1 | hsa-miR-186-5p | LINC00158 | hsa-miR-186-5p | RP11-322E11.2 |
| hsa-miR-34a-5p | RP4-673M15.1 | hsa-miR-25-3p | AC093375.1 | hsa-miR-374b-5p | RP11-322E11.2 |
| hsa-miR-143-3p | RP11-370A5.2 | hsa-miR-199a-5p | RP11-260M2.1 | hsa-miR-421 | RP11-322E11.2 |
| hsa-miR-27b-3p | RP11-223P11.3 | hsa-miR-221-3p | AF067845.1 | hsa-miR-140-3p | RP11-317N8.3 |
| hsa-miR-374b-5p | AC007163.3 | hsa-miR-186-5p | RP11-37L2.1 | hsa-miR-186-5p | RP11-244M2.1 |
| hsa-miR-27b-3p | RP11-707P17.2 | hsa-miR-27b-3p | LINC00996 | hsa-miR-146b-5p | RP11-244M2.1 |
| hsa-miR-34a-5p | RP11-544A12.4 | hsa-miR-186-5p | LINC00996 | hsa-miR-421 | RP11-244M2.1 |
| hsa-miR-27b-3p | RP11-278A23.2 | hsa-miR-27b-3p | RP11-44N11.1 | hsa-miR-186-5p | AC005220.3 |
| hsa-miR-26b-5p | LL21NC02-1C16.2 | hsa-miR-27b-3p | AC025171.1 | hsa-miR-146b-5p | AC005220.3 |
| hsa-miR-125b-5p | RP11-106D4.2 | hsa-miR-143-3p | AC025171.1 | hsa-miR-421 | AC003984.1 |
| hsa-miR-146b-5p | RP11-429B14.4 | hsa-miR-26b-5p | AC025171.1 | hsa-miR-143-3p | AC003984.1 |
| hsa-miR-221-3p | RP11-563P16.1 | hsa-miR-27b-3p | ENOX1-AS1 | hsa-miR-26b-5p | AC003984.1 |
| hsa-miR-143-3p | RP11-636O21.1 | hsa-miR-34a-5p | CTD-2560E9.5 | hsa-miR-374b-5p | AC003984.1 |
| hsa-miR-27b-3p | AC105402.4 | hsa-miR-499a-5p | RP11-519G16.3 | hsa-miR-143-3p | RP11-944C7.1 |
| hsa-miR-421 | AC105402.4 | hsa-miR-374b-5p | RP11-519G16.3 | hsa-miR-186-5p | RP11-944C7.1 |
| hsa-miR-186-5p | HNRNPU-AS1 | hsa-miR-140-3p | LINC01183 | hsa-miR-34a-5p | CTC-499B15.7 |
| hsa-miR-26b-5p | HNRNPU-AS1 | hsa-miR-499a-5p | CTD-2288O8.1 | hsa-miR-143-3p | RP11-307P5.1 |
| hsa-miR-27b-3p | HNRNPU-AS1 | hsa-miR-27b-3p | RP11-354K1.1 | hsa-miR-27b-3p | RP11-307P5.1 |
| hsa-miR-421 | RP11-480D4.6 | hsa-miR-143-3p | RP11-373E16.3 | hsa-miR-140-3p | RP11-255M2.1 |
| hsa-miR-148a-3p | HOTAIR | hsa-miR-186-5p | RP11-373E16.3 | hsa-miR-25-3p | RP11-346J10.1 |
| hsa-miR-148b-3p | HOTAIR | hsa-miR-499a-5p | RP11-373E16.3 | hsa-miR-27b-3p | RP5-1116H23.4 |
| hsa-miR-221-3p | HOTAIR | hsa-miR-146b-5p | RP11-373E16.3 | hsa-miR-140-3p | VAC14-AS1 |
| hsa-miR-148b-3p | AC002064.4 | hsa-miR-26b-5p | AC008277.1 | hsa-miR-421 | EIF1AX-AS1 |
| hsa-miR-186-5p | C3orf67-AS1 | hsa-miR-186-5p | AC006548.28 | hsa-miR-143-3p | RP11-68I3.5 |
| hsa-miR-140-3p | C3orf67-AS1 | hsa-miR-27b-3p | AC006548.28 | hsa-miR-27b-3p | LINC01179 |
| hsa-miR-27b-3p | RP11-528G1.2 | hsa-miR-125b-5p | AC006548.28 | hsa-miR-34a-5p | RP11-752D24.2 |
| hsa-miR-125b-5p | RP11-406H4.1 | hsa-miR-421 | AC006548.28 | hsa-miR-186-5p | RP11-752D24.2 |

| | | | | | |
|---|---|---|---|---|---|
| hsa-miR-186-5p | AC096669.1 | hsa-miR-146b-5p | AC006548.28 | hsa-miR-186-5p | RP11-545A16.4 |
| hsa-miR-199a-5p | AC096669.1 | hsa-miR-140-3p | AC006548.28 | hsa-miR-186-5p | RP11-73O6.3 |
| hsa-miR-25-3p | LINC01428 | hsa-miR-374b-5p | AC006548.28 | hsa-miR-34a-5p | RP11-168K11.3 |
| hsa-miR-34a-5p | RP4-695O20.1 | hsa-miR-378h | AC006548.28 | hsa-miR-25-3p | RP11-664D7.4 |
| hsa-miR-27b-3p | AP000695.6 | hsa-miR-378a-3p | AC006548.28 | hsa-miR-374b-5p | RP11-79H23.3 |
| hsa-miR-26b-5p | RP4-694A7.2 | hsa-miR-26b-5p | AC006548.28 | hsa-miR-27b-3p | RP11-79H23.3 |
| hsa-miR-143-3p | HNF4A-AS1 | hsa-miR-143-3p | AC006548.28 | hsa-miR-499a-5p | RP11-79H23.3 |
| hsa-miR-499a-5p | RP11-366L20.2 | hsa-miR-34a-5p | AC006548.28 | hsa-miR-27b-3p | RP11-981G7.1 |
| hsa-miR-378h | RP11-366L20.2 | hsa-miR-148a-3p | AC006548.28 | hsa-miR-146b-5p | RP11-981G7.1 |
| hsa-miR-27b-3p | RP11-449P15.2 | hsa-miR-199a-5p | AC006548.28 | hsa-miR-421 | RP11-981G7.1 |
| hsa-miR-143-3p | RP11-91K8.5 | hsa-miR-148b-3p | AC006548.28 | hsa-miR-186-5p | RP11-981G7.1 |
| hsa-miR-26b-5p | AC005498.3 | hsa-miR-25-3p | AC006548.28 | hsa-miR-421 | IGBP1-AS1 |
| hsa-miR-148b-3p | AC005498.3 | hsa-miR-499a-5p | AC006548.28 | hsa-miR-148a-3p | CTC-523E23.1 |
| hsa-miR-25-3p | AC159540.1 | hsa-miR-221-3p | AC006548.28 | hsa-miR-34a-5p | RP11-617F23.1 |
| hsa-miR-186-5p | AC159540.1 | hsa-miR-27b-3p | LINC00925 | hsa-miR-146b-5p | RP11-617F23.1 |
| hsa-miR-34a-5p | CTD-2537I9.12 | hsa-miR-125b-5p | FLJ16779 | hsa-miR-27b-3p | RP1-239B22.5 |
| hsa-miR-25-3p | GTF3C2-AS1 | hsa-miR-34a-5p | FLJ16779 | hsa-miR-27b-3p | CTA-797E19.1 |
| hsa-miR-378h | AC007292.6 | hsa-miR-186-5p | RP11-686D22.4 | hsa-miR-25-3p | CTD-2332E11.2 |
| hsa-miR-27b-3p | AC078942.1 | hsa-miR-27b-3p | FAM66A | hsa-miR-34a-5p | SIK3-IT1 |
| hsa-miR-125b-5p | RP3-365O12.2 | hsa-miR-125b-5p | FAM66A | hsa-miR-143-3p | CTA-243E7.1 |
| hsa-miR-186-5p | RP4-671O14.6 | hsa-miR-143-3p | LOXL1-AS1 | hsa-miR-148a-3p | CTA-243E7.1 |
| hsa-miR-143-3p | RP4-671O14.6 | hsa-miR-186-5p | LOXL1-AS1 | hsa-miR-499a-5p | CTA-243E7.1 |
| hsa-miR-125b-5p | RP4-671O14.6 | hsa-miR-374b-5p | LOXL1-AS1 | hsa-miR-27b-3p | CTA-243E7.1 |
| hsa-miR-25-3p | RP4-671O14.6 | hsa-miR-421 | LOXL1-AS1 | hsa-miR-148b-3p | CTA-243E7.1 |
| hsa-miR-421 | TBX5-AS1 | hsa-miR-34a-5p | LINC00595 | hsa-miR-125b-5p | CTA-243E7.1 |
| hsa-miR-378a-3p | TBX5-AS1 | hsa-miR-378h | EPN2-AS1 | hsa-miR-221-3p | CTA-243E7.1 |
| hsa-miR-378h | TBX5-AS1 | hsa-miR-378a-3p | EPN2-AS1 | hsa-miR-26b-5p | CTA-243E7.1 |
| hsa-miR-421 | RP11-613F22.8 | hsa-miR-26b-5p | RP11-430H10.1 | hsa-miR-374b-5p | RP11-61K9.2 |

| | | | | | |
|---|---|---|---|---|---|
| hsa-miR-186-5p | RP11-613F22.8 | hsa-miR-378a-3p | CTD-3193K9.3 | hsa-miR-378a-3p | TEX41 |
| hsa-miR-26b-5p | DDX11-AS1 | hsa-miR-378h | CTD-3193K9.3 | hsa-miR-143-3p | TEX41 |
| hsa-miR-26b-5p | RP3-473B4.3 | hsa-miR-148b-3p | LA16c-390H2.4 | hsa-miR-378h | TEX41 |
| hsa-miR-186-5p | TMCC1-AS1 | hsa-miR-148a-3p | LA16c-390H2.4 | hsa-miR-148b-3p | TEX41 |
| hsa-miR-199a-5p | TMCC1-AS1 | hsa-miR-34a-5p | LINC01252 | hsa-miR-148a-3p | TEX41 |
| hsa-miR-199a-5p | CTD-2542L18.1 | hsa-miR-199a-5p | CTD-2525I3.5 | hsa-miR-186-5p | TEX41 |
| hsa-miR-421 | CTD-2542L18.1 | hsa-miR-186-5p | AC092675.3 | hsa-miR-421 | TEX41 |
| hsa-miR-143-3p | RP11-903H12.3 | hsa-miR-186-5p | CTC-573N18.1 | hsa-miR-25-3p | TEX41 |
| hsa-miR-148a-3p | RTCA-AS1 | hsa-miR-148a-3p | RP11-70D24.2 | hsa-miR-27b-3p | TEX41 |
| hsa-miR-148b-3p | RTCA-AS1 | hsa-miR-148b-3p | RP11-70D24.2 | hsa-miR-125b-5p | TEX41 |
| hsa-miR-148a-3p | RP11-91K9.1 | hsa-miR-27b-3p | CTC-499B15.6 | hsa-miR-186-5p | NEAT1 |
| hsa-miR-148b-3p | RP11-91K9.1 | hsa-miR-143-3p | RP11-24F11.2 | hsa-miR-27b-3p | NEAT1 |
| hsa-miR-143-3p | AC004837.5 | hsa-miR-27b-3p | CTD-2240J17.1 | hsa-miR-140-3p | NEAT1 |
| hsa-miR-34a-5p | AC010127.3 | hsa-miR-143-3p | CTA-929C8.6 | hsa-miR-34a-5p | NEAT1 |
| hsa-miR-421 | AC010127.3 | hsa-miR-199a-5p | CTA-929C8.6 | hsa-miR-26b-5p | NEAT1 |
| hsa-miR-186-5p | AC010127.3 | hsa-miR-186-5p | CTA-929C8.6 | hsa-miR-146b-5p | NEAT1 |
| hsa-miR-140-3p | AC010127.3 | hsa-miR-146b-5p | F10-AS1 | hsa-miR-148a-3p | NEAT1 |
| hsa-miR-27b-3p | CTB-138E5.1 | hsa-miR-146b-5p | RP11-415J8.7 | hsa-miR-125b-5p | NEAT1 |
| hsa-miR-143-3p | RP5-836N17.4 | hsa-miR-374b-5p | AC011343.1 | hsa-miR-148b-3p | NEAT1 |
| hsa-miR-374b-5p | LINC00461 | hsa-miR-34a-5p | AC011343.1 | hsa-miR-374b-5p | NEAT1 |
| hsa-miR-27b-3p | LINC00461 | hsa-miR-140-3p | AC011343.1 | hsa-miR-421 | NEAT1 |
| hsa-miR-125b-5p | LINC00461 | hsa-miR-143-3p | AC011343.1 | hsa-miR-499a-5p | NEAT1 |
| hsa-miR-221-3p | LINC00461 | hsa-miR-421 | RP5-1057B20.3 | hsa-miR-27b-3p | RP11-1085N6.2 |
| hsa-miR-25-3p | LINC00461 | hsa-miR-186-5p | RP5-1057B20.3 | hsa-miR-27b-3p | C9orf41-AS1 |
| hsa-miR-140-3p | RP1-20B11.2 | hsa-miR-27b-3p | RP11-674E16.4 | hsa-miR-148a-3p | NFIA-AS2 |
| hsa-miR-26b-5p | RP11-348N5.7 | hsa-miR-374b-5p | RP11-674E16.4 | hsa-miR-148b-3p | NFIA-AS2 |
| hsa-miR-27b-3p | LINC01280 | hsa-miR-140-3p | CTA-544A11.1 | hsa-miR-27b-3p | RP11-89K21.1 |
| hsa-miR-421 | RP11-804N13.1 | hsa-miR-146b-5p | CTA-544A11.1 | hsa-miR-186-5p | RP11-89K21.1 |

| | | | | | |
|---|---|---|---|---|---|
| hsa-miR-186-5p | RP11-804N13.1 | hsa-miR-186-5p | CTA-544A11.1 | hsa-miR-199a-5p | RP11-65M17.3 |
| hsa-miR-374b-5p | RP13-192B19.2 | hsa-miR-499a-5p | CTA-544A11.1 | hsa-miR-374b-5p | RP11-65M17.3 |
| hsa-miR-186-5p | RP13-192B19.2 | hsa-miR-378a-3p | CTA-544A11.1 | hsa-miR-221-3p | RP11-815J21.1 |
| hsa-miR-143-3p | RP11-737O24.2 | hsa-miR-499a-5p | RP11-2I17.4 | hsa-miR-143-3p | LINC01146 |
| hsa-miR-199a-5p | RP11-798M19.6 | hsa-miR-186-5p | RP11-310H4.3 | hsa-miR-374b-5p | RP11-259N19.1 |
| hsa-miR-143-3p | LA16c-361A3.3 | hsa-miR-27b-3p | RP11-127L20.3 | hsa-miR-378a-3p | RP11-234K24.3 |
| hsa-miR-148b-3p | LA16c-361A3.3 | hsa-miR-148b-3p | CTD-2334D19.1 | hsa-miR-26b-5p | RP11-234K24.3 |
| hsa-miR-148a-3p | LA16c-361A3.3 | hsa-miR-148a-3p | CTD-2334D19.1 | hsa-miR-378h | RP11-234K24.3 |
| hsa-miR-146b-5p | RP11-553L6.2 | hsa-miR-27b-3p | RP11-379K22.2 | hsa-miR-27b-3p | RP11-105N14.2 |
| hsa-miR-499a-5p | RP11-553L6.2 | hsa-miR-25-3p | RP11-379K22.2 | hsa-miR-146b-5p | IDI2-AS1 |
| hsa-miR-499a-5p | LINC00115 | hsa-miR-146b-5p | RP3-466P17.1 | hsa-miR-143-3p | RP5-1057I20.2 |
| hsa-miR-27b-3p | RP11-837J7.4 | hsa-miR-186-5p | RP3-466P17.1 | hsa-miR-374b-5p | RP11-586K2.1 |
| hsa-miR-125b-5p | RP11-227G15.9 | hsa-miR-378a-3p | RP11-3P22.2 | hsa-miR-125b-5p | RP11-586K2.1 |
| hsa-miR-26b-5p | RP4-533D7.4 | hsa-miR-378h | RP11-3P22.2 | hsa-miR-199a-5p | RP11-586K2.1 |
| hsa-miR-26b-5p | AP001429.1 | hsa-miR-125b-5p | CTD-2201I18.1 | hsa-miR-125b-5p | RP11-52J3.2 |
| hsa-miR-27b-3p | AP001429.1 | hsa-miR-186-5p | CTA-212A2.3 | hsa-miR-148a-3p | CTD-3131K8.2 |
| hsa-miR-374b-5p | RP3-523K23.2 | hsa-miR-143-3p | CTA-212A2.3 | hsa-miR-148b-3p | CTD-3131K8.2 |
| hsa-miR-25-3p | RP3-523K23.2 | hsa-miR-34a-5p | CTA-212A2.3 | hsa-miR-26b-5p | RP11-513D5.2 |
| hsa-miR-378h | RP11-323F24.4 | hsa-miR-221-3p | CTA-212A2.3 | hsa-miR-186-5p | KB-1552D7.2 |
| hsa-miR-378a-3p | RP11-323F24.4 | hsa-miR-378a-3p | RP11-374M1.5 | hsa-miR-25-3p | KB-1552D7.2 |
| hsa-miR-34a-5p | RP11-219E7.2 | hsa-miR-378h | RP11-374M1.5 | hsa-miR-125b-5p | KB-1552D7.2 |
| hsa-miR-27b-3p | RP11-586D19.2 | hsa-miR-27b-3p | RP11-855A2.3 | hsa-miR-378a-3p | RP11-211N11.5 |
| hsa-miR-199a-5p | RP11-757G1.6 | hsa-miR-421 | RP11-855A2.3 | hsa-miR-378h | RP11-211N11.5 |
| hsa-miR-221-3p | RP11-757G1.6 | hsa-miR-125b-5p | RP11-855A2.3 | hsa-miR-199a-5p | AC003003.5 |
| hsa-miR-26b-5p | RP11-757G1.6 | hsa-miR-186-5p | RP11-731J8.2 | hsa-miR-25-3p | CTB-5E10.3 |
| hsa-miR-125b-5p | RP11-757G1.6 | hsa-miR-34a-5p | RP11-731J8.2 | hsa-miR-27b-3p | RP11-689B22.2 |
| hsa-miR-186-5p | RP11-757G1.6 | hsa-miR-143-3p | AC099684.1 | hsa-miR-143-3p | LINC00528 |
| hsa-miR-374b-5p | RP11-757G1.6 | hsa-miR-125b-5p | AC099684.1 | hsa-miR-140-3p | LINC00528 |

| | | | | | |
|---|---|---|---|---|---|
| hsa-miR-34a-5p | RP11-757G1.6 | hsa-miR-125b-5p | RP11-42I10.1 | hsa-miR-26b-5p | RP11-181K12.2 |
| hsa-miR-421 | LINC01108 | hsa-miR-26b-5p | RP13-991F5.2 | hsa-miR-186-5p | RP11-281P23.1 |
| hsa-miR-374b-5p | STEAP3-AS1 | hsa-miR-27b-3p | RP13-991F5.2 | hsa-miR-499a-5p | TBL1XR1-AS1 |
| hsa-miR-143-3p | GK-IT1 | hsa-miR-186-5p | AC002429.5 | hsa-miR-143-3p | RP11-266K4.9 |
| hsa-miR-143-3p | LINC00284 | hsa-miR-199a-5p | CCDC26 | hsa-miR-499a-5p | RP11-266K4.9 |
| hsa-miR-27b-3p | LINC00284 | hsa-miR-499a-5p | RP11-255E6.6 | hsa-miR-26b-5p | RP11-280K24.4 |
| hsa-miR-125b-5p | LINC01152 | hsa-miR-125b-5p | CTD-2231E14.8 | hsa-miR-186-5p | RP11-280K24.4 |
| hsa-miR-499a-5p | LINC00222 | hsa-miR-148a-3p | RP11-47L3.1 | hsa-miR-125b-5p | RP5-875O13.1 |
| hsa-miR-26b-5p | LINC00222 | hsa-miR-148b-3p | RP11-47L3.1 | hsa-miR-143-3p | RP5-875O13.1 |
| hsa-miR-143-3p | CTD-2269F5.1 | hsa-miR-34a-5p | ZIM2-AS1 | hsa-miR-146b-5p | LINC00494 |
| hsa-miR-421 | CTD-2269F5.1 | hsa-miR-199a-5p | ZIM2-AS1 | hsa-miR-140-3p | GCSAML-AS1 |
| hsa-miR-25-3p | AC018730.4 | hsa-miR-143-3p | ZIM2-AS1 | hsa-miR-27b-3p | GCSAML-AS1 |
| hsa-miR-26b-5p | RP4-635E18.8 | hsa-miR-378a-3p | DNMBP-AS1 | hsa-miR-421 | GCSAML-AS1 |
| hsa-miR-421 | RP4-635E18.8 | hsa-miR-378h | DNMBP-AS1 | hsa-miR-148a-3p | GCSAML-AS1 |
| hsa-miR-421 | CTD-2377O17.1 | hsa-miR-143-3p | DNMBP-AS1 | hsa-miR-199a-5p | RP11-320P7.2 |
| hsa-miR-148b-3p | DHRS4-AS1 | hsa-miR-25-3p | AC004775.5 | hsa-miR-146b-5p | RP11-320P7.2 |
| hsa-miR-143-3p | DHRS4-AS1 | hsa-miR-378a-3p | RP11-367J11.2 | hsa-miR-27b-3p | LURAP1L-AS1 |
| hsa-miR-148a-3p | DHRS4-AS1 | hsa-miR-378h | RP11-367J11.2 | hsa-miR-421 | ZFAS1 |
| hsa-miR-186-5p | RP11-329J18.3 | hsa-miR-143-3p | RP11-367J11.2 | hsa-miR-27b-3p | ZFAS1 |
| hsa-miR-421 | TMLHE-AS1 | hsa-miR-27b-3p | RP11-367J11.2 | hsa-miR-199a-5p | RP5-1142A6.9 |
| hsa-miR-186-5p | RP11-363J20.1 | hsa-miR-186-5p | RP5-1116H23.6 | hsa-miR-186-5p | CTD-2292P10.4 |
| hsa-miR-27b-3p | SPATA3-AS1 | hsa-miR-499a-5p | LINC01343 | hsa-miR-186-5p | RP11-646I6.6 |
| hsa-miR-26b-5p | AC009505.2 | hsa-miR-34a-5p | AC147651.5 | hsa-miR-421 | AC068039.4 |
| hsa-miR-34a-5p | AC009505.2 | hsa-miR-125b-5p | RP11-141J13.5 | hsa-miR-25-3p | AC068039.4 |
| hsa-miR-143-3p | RP11-445P17.3 | hsa-miR-34a-5p | NAMA | hsa-miR-26b-5p | AC068039.4 |
| hsa-miR-186-5p | RP11-517I3.1 | hsa-miR-146b-5p | RP11-789C2.1 | hsa-miR-374b-5p | AC068039.4 |
| hsa-miR-421 | RP11-705C15.3 | hsa-miR-146b-5p | RP11-417E7.2 | hsa-miR-186-5p | AC068039.4 |
| hsa-miR-186-5p | RP11-705C15.3 | hsa-miR-186-5p | RP11-417E7.2 | hsa-miR-146b-5p | RP11-325K4.2 |

| | | | | | |
|---|---|---|---|---|---|
| hsa-miR-25-3p | LLNLR-470E3.1 | hsa-miR-34a-5p | CTA-414D7.1 | hsa-miR-143-3p | RP11-598F7.4 |
| hsa-miR-146b-5p | LLNLR-470E3.1 | hsa-miR-421 | CTA-414D7.1 | hsa-miR-186-5p | RP11-598F7.4 |
| hsa-miR-125b-5p | RP11-381N20.1 | hsa-miR-125b-5p | CTA-414D7.1 | hsa-miR-421 | RP11-689K5.3 |
| hsa-miR-148a-3p | RP11-73M18.8 | hsa-miR-374b-5p | RP11-556E13.1 | hsa-miR-143-3p | AC096579.13 |
| hsa-miR-148b-3p | RP11-73M18.8 | hsa-miR-143-3p | LINC01141 | hsa-miR-186-5p | RP11-111E14.1 |
| hsa-miR-421 | RP11-434D9.1 | hsa-miR-186-5p | CTB-129O4.1 | hsa-miR-26b-5p | CTA-331P3.1 |
| hsa-miR-146b-5p | RP11-434D9.1 | hsa-miR-143-3p | RP11-158I9.5 | hsa-miR-499a-5p | CTA-331P3.1 |
| hsa-miR-143-3p | MLK7-AS1 | hsa-miR-125b-5p | AP001055.6 | hsa-miR-143-3p | AC019117.1 |
| hsa-miR-186-5p | MLK7-AS1 | hsa-miR-146b-5p | RP11-75L1.1 | hsa-miR-140-3p | AC019117.1 |
| hsa-miR-146b-5p | MLK7-AS1 | hsa-miR-186-5p | RP11-75L1.1 | hsa-miR-499a-5p | AC019181.2 |
| hsa-miR-27b-3p | RP11-356J5.12 | hsa-miR-378h | RP4-594I10.3 | hsa-miR-34a-5p | LINC00640 |
| hsa-miR-374b-5p | RP11-356J5.12 | hsa-miR-378a-3p | RP4-594I10.3 | hsa-miR-221-3p | RP11-1035H13.2 |
| hsa-miR-186-5p | RP11-356J5.12 | hsa-miR-27b-3p | RP4-594I10.3 | hsa-miR-26b-5p | CTD-2506J14.1 |
| hsa-miR-199a-5p | RP11-356J5.12 | hsa-miR-146b-5p | PWRN1 | hsa-miR-374b-5p | RP11-96C23.9 |
| hsa-miR-140-3p | RP11-673E1.4 | hsa-miR-186-5p | PWRN1 | hsa-miR-140-3p | RP11-446J8.1 |
| hsa-miR-199a-5p | RP11-673E1.4 | hsa-miR-421 | PWRN1 | hsa-miR-125b-5p | AC096669.3 |
| hsa-miR-374b-5p | RP11-560G2.1 | hsa-miR-140-3p | PWRN1 | hsa-miR-34a-5p | AC096669.3 |
| hsa-miR-421 | RP4-798A10.2 | hsa-miR-499a-5p | PWRN1 | hsa-miR-378h | CTC-366B18.4 |
| hsa-miR-143-3p | RP11-275I4.2 | hsa-miR-125b-5p | PWRN1 | hsa-miR-378a-3p | CTC-366B18.4 |
| hsa-miR-25-3p | RP11-279O17.1 | hsa-miR-34a-5p | RP11-405M12.4 | hsa-miR-148b-3p | CTC-366B18.4 |
| hsa-miR-499a-5p | SLC16A1-AS1 | hsa-miR-27b-3p | RP11-313D6.3 | hsa-miR-374b-5p | CTC-366B18.4 |
| hsa-miR-143-3p | SLC16A1-AS1 | hsa-miR-143-3p | RP11-161H23.9 | hsa-miR-125b-5p | RP11-244F12.3 |
| hsa-miR-140-3p | AC007292.3 | hsa-miR-34a-5p | F11-AS1 | hsa-miR-148b-3p | RP11-244F12.3 |
| hsa-miR-27b-3p | RP11-840I19.3 | hsa-miR-140-3p | RP11-463D19.1 | hsa-miR-140-3p | RP11-528N21.1 |
| hsa-miR-34a-5p | RP11-840I19.3 | hsa-miR-199a-5p | RP11-575L7.8 | hsa-miR-186-5p | RP11-528N21.1 |
| hsa-miR-140-3p | RP1-105O18.1 | hsa-miR-186-5p | RP3-325F22.5 | hsa-miR-186-5p | RP13-487P22.1 |
| hsa-miR-378a-3p | LINC00313 | hsa-miR-143-3p | AC005253.2 | hsa-miR-143-3p | RP11-104N10.1 |
| hsa-miR-378h | LINC00313 | hsa-miR-499a-5p | RP11-84C10.4 | hsa-miR-26b-5p | CTD-2044J15.1 |

| | | | | | |
|---|---|---|---|---|---|
| hsa-miR-374b-5p | RP11-130L8.2 | hsa-miR-26b-5p | RP11-84C10.4 | hsa-miR-148b-3p | LINC00087 |
| hsa-miR-421 | RP11-309M7.1 | hsa-miR-27b-3p | RP11-84C10.4 | hsa-miR-378a-3p | DIO3OS |
| hsa-miR-221-3p | RP11-96D1.10 | hsa-miR-374b-5p | LINC00890 | hsa-miR-378h | DIO3OS |
| hsa-miR-374b-5p | RP11-338N10.3 | hsa-miR-125b-5p | PSMG3-AS1 | hsa-miR-374b-5p | SNHG22 |
| hsa-miR-378h | AC093818.1 | hsa-miR-34a-5p | AP001628.6 | hsa-miR-148b-3p | RP11-433A10.3 |
| hsa-miR-378a-3p | AC093818.1 | hsa-miR-125b-5p | RP11-288L9.4 | hsa-miR-148a-3p | RP11-433A10.3 |
| hsa-miR-125b-5p | RP11-573D15.2 | hsa-miR-27b-3p | RP11-713D19.1 | hsa-miR-26b-5p | AC053503.4 |
| hsa-miR-186-5p | AC078941.1 | hsa-miR-125b-5p | LINC01224 | hsa-miR-146b-5p | RP11-142C4.6 |
| hsa-miR-148b-3p | AC078941.1 | hsa-miR-186-5p | LINC01224 | hsa-miR-148b-3p | RP11-142C4.6 |
| hsa-miR-148a-3p | AC078941.1 | hsa-miR-34a-5p | LINC01224 | hsa-miR-34a-5p | RP11-142C4.6 |
| hsa-miR-27b-3p | RAPGEF4-AS1 | hsa-miR-143-3p | LINC01224 | hsa-miR-186-5p | AC010976.2 |
| hsa-miR-186-5p | RAPGEF4-AS1 | hsa-miR-27b-3p | LINC01224 | hsa-miR-27b-3p | LINC01567 |
| hsa-miR-25-3p | RAPGEF4-AS1 | hsa-miR-146b-5p | RP11-166P13.4 | hsa-miR-34a-5p | RP11-171I2.4 |
| hsa-miR-221-3p | RAPGEF4-AS1 | hsa-miR-34a-5p | RP5-1071N3.1 | hsa-miR-186-5p | RP11-274H2.5 |
| hsa-miR-186-5p | AC012531.25 | hsa-miR-148b-3p | PSORS1C3 | hsa-miR-25-3p | RP11-274H2.5 |
| hsa-miR-374b-5p | AC012531.25 | hsa-miR-378h | CTD-3128G10.6 | hsa-miR-374b-5p | RP11-152L20.3 |
| hsa-miR-140-3p | RP11-802E16.3 | hsa-miR-378a-3p | CTD-3128G10.6 | hsa-miR-34a-5p | RP11-152L20.3 |
| hsa-miR-374b-5p | CTD-2506P8.6 | hsa-miR-125b-5p | RP1-67A8.3 | hsa-miR-140-3p | GS1-304P7.3 |
| hsa-miR-499a-5p | KB-1208A12.3 | hsa-miR-199a-5p | RP4-800J21.3 | hsa-miR-140-3p | RP11-661A12.12 |
| hsa-miR-148a-3p | RP11-576D8.4 | hsa-miR-186-5p | LEMD1-AS1 | hsa-miR-26b-5p | CTA-256D12.11 |
| hsa-miR-148b-3p | RP11-576D8.4 | hsa-miR-146b-5p | LEMD1-AS1 | hsa-miR-374b-5p | RP11-713N11.6 |
| hsa-miR-221-3p | RP11-576D8.4 | hsa-miR-27b-3p | FOXD2-AS1 | hsa-miR-143-3p | RP11-480A16.1 |
| hsa-miR-186-5p | AC093690.1 | hsa-miR-26b-5p | RP11-588L15.2 | hsa-miR-140-3p | RP11-480A16.1 |
| hsa-miR-27b-3p | AC093690.1 | hsa-miR-27b-3p | RP11-104O19.4 | hsa-miR-186-5p | RP11-399O19.9 |
| hsa-miR-34a-5p | AC093690.1 | hsa-miR-34a-5p | LINC00621 | hsa-miR-125b-5p | RP1-199J3.7 |
| hsa-miR-148b-3p | KIRREL-IT1 | hsa-miR-27b-3p | LINC01562 | hsa-miR-199a-5p | LINC01233 |
| hsa-miR-148a-3p | KIRREL-IT1 | hsa-miR-27b-3p | RP11-18H21.3 | hsa-miR-125b-5p | RP11-301L8.2 |
| hsa-miR-421 | C1orf132 | hsa-miR-140-3p | CTC-498M16.4 | hsa-miR-125b-5p | RP11-165M1.3 |

| | | | | | |
|---|---|---|---|---|---|
| hsa-miR-27b-3p | C1orf132 | hsa-miR-186-5p | RP11-30K9.4 | hsa-miR-186-5p | RP11-1007G5.2 |
| hsa-miR-378a-3p | C1orf132 | hsa-miR-27b-3p | RP6-109B7.2 | hsa-miR-378a-3p | RP11-1007G5.2 |
| hsa-miR-378h | C1orf132 | hsa-miR-26b-5p | RP13-616I3.1 | hsa-miR-143-3p | RP11-1007G5.2 |
| hsa-miR-125b-5p | C1orf132 | hsa-miR-499a-5p | RP11-362F19.1 | hsa-miR-378h | RP11-1007G5.2 |
| hsa-miR-140-3p | C1orf132 | hsa-miR-221-3p | RP11-362F19.1 | hsa-miR-221-3p | RP11-147L13.8 |
| hsa-miR-34a-5p | C1orf132 | hsa-miR-34a-5p | RP11-362F19.1 | hsa-miR-146b-5p | RP11-147L13.8 |
| hsa-miR-34a-5p | RP11-554I8.2 | hsa-miR-27b-3p | RP11-362F19.1 | hsa-miR-26b-5p | RP11-147L13.8 |
| hsa-miR-27b-3p | RP11-9N12.2 | hsa-miR-125b-5p | RP11-362F19.1 | hsa-miR-374b-5p | RP11-147L13.8 |
| hsa-miR-186-5p | RP11-9N12.2 | hsa-miR-146b-5p | RP11-981G7.3 | hsa-miR-125b-5p | RP11-478K15.6 |
| hsa-miR-27b-3p | RP11-61E11.1 | hsa-miR-378a-3p | RP11-1079K10.3 | hsa-miR-27b-3p | RP11-478K15.6 |
| hsa-miR-378h | RP11-61E11.1 | hsa-miR-378h | RP11-1079K10.3 | hsa-miR-125b-5p | KDM5C-IT1 |
| hsa-miR-378a-3p | RP11-61E11.1 | hsa-miR-125b-5p | LINC00838 | hsa-miR-143-3p | RP11-768F21.1 |
| hsa-miR-374b-5p | AC025811.3 | hsa-miR-186-5p | NPPA-AS1 | hsa-miR-143-3p | RP11-116D17.5 |
| hsa-miR-186-5p | AC025811.3 | hsa-miR-148a-3p | RP11-265D19.6 | hsa-miR-34a-5p | RP1-69D17.4 |
| hsa-miR-186-5p | RP11-438B23.2 | hsa-miR-148b-3p | RP11-265D19.6 | hsa-miR-125b-5p | RP1-69D17.4 |
| hsa-miR-125b-5p | RP11-438B23.2 | hsa-miR-186-5p | RP11-20B24.7 | hsa-miR-143-3p | RP4-647C14.2 |
| hsa-miR-421 | RP11-438B23.2 | hsa-miR-199a-5p | AC079466.1 | hsa-miR-125b-5p | RP11-386G11.10 |
| hsa-miR-199a-5p | RP11-438B23.2 | hsa-miR-125b-5p | AC079466.1 | hsa-miR-221-3p | AC069155.1 |
| hsa-miR-143-3p | RP11-438B23.2 | hsa-miR-378a-3p | AC079466.1 | hsa-miR-26b-5p | RP4-760C5.3 |
| hsa-miR-199a-5p | CTD-2369P2.8 | hsa-miR-378h | AC079466.1 | hsa-miR-143-3p | RP4-760C5.3 |
| hsa-miR-143-3p | CTA-992D9.8 | hsa-miR-146b-5p | AC079466.1 | hsa-miR-378a-3p | RP1-17K7.2 |
| hsa-miR-27b-3p | CTA-992D9.8 | hsa-miR-26b-5p | AC079466.1 | hsa-miR-378h | RP1-17K7.2 |
| hsa-miR-186-5p | CTA-992D9.8 | hsa-miR-34a-5p | AC079466.1 | hsa-miR-146b-5p | AC074289.1 |
| hsa-miR-146b-5p | AC000123.4 | hsa-miR-148b-3p | AC079466.1 | hsa-miR-186-5p | RP11-262I2.2 |
| hsa-miR-140-3p | AC000123.4 | hsa-miR-221-3p | AC079466.1 | hsa-miR-125b-5p | RP11-400F19.18 |
| hsa-miR-186-5p | RP11-703M24.5 | hsa-miR-186-5p | AC079466.1 | hsa-miR-148b-3p | RP11-35O15.2 |
| hsa-miR-27b-3p | RP1-45C12.1 | hsa-miR-125b-5p | RP11-732A19.9 | hsa-miR-186-5p | RP11-631N16.4 |
| hsa-miR-421 | AC009784.3 | hsa-miR-199a-5p | RP11-478P10.1 | hsa-miR-186-5p | RP11-180N14.1 |

| | | | | | |
|---|---|---|---|---|---|
| hsa-miR-374b-5p | RP5-921G16.1 | hsa-miR-26b-5p | RP11-478P10.1 | hsa-miR-186-5p | CTB-133G6.2 |
| hsa-miR-140-3p | RP11-102L12.2 | hsa-miR-143-3p | RP11-478P10.1 | hsa-miR-186-5p | CTD-2620I22.3 |
| hsa-miR-27b-3p | RP13-735L24.1 | hsa-miR-27b-3p | LINC01524 | hsa-miR-221-3p | AC073326.3 |
| hsa-miR-186-5p | RP13-735L24.1 | hsa-miR-26b-5p | LINC01524 | hsa-miR-26b-5p | AC073326.3 |
| hsa-miR-221-3p | RP13-735L24.1 | hsa-miR-186-5p | UG0898H09 | hsa-miR-146b-5p | SOX2-OT |
| hsa-miR-199a-5p | RP11-267M23.3 | hsa-miR-374b-5p | UG0898H09 | hsa-miR-143-3p | SOX2-OT |
| hsa-miR-199a-5p | RP11-57A1.1 | hsa-miR-421 | UG0898H09 | hsa-miR-199a-5p | SOX2-OT |
| hsa-miR-378a-3p | RP11-709D24.8 | hsa-miR-140-3p | UG0898H09 | hsa-miR-27b-3p | SOX2-OT |
| hsa-miR-378h | RP11-709D24.8 | hsa-miR-27b-3p | UG0898H09 | hsa-miR-374b-5p | SOX2-OT |
| hsa-miR-140-3p | AC068492.1 | hsa-miR-143-3p | RP11-778J15.1 | hsa-miR-186-5p | SOX2-OT |
| hsa-miR-125b-5p | RP4-591N18.2 | hsa-miR-27b-3p | RP1-179N16.6 | hsa-miR-421 | SOX2-OT |
| hsa-miR-34a-5p | AC004951.6 | hsa-miR-25-3p | LINC00977 | hsa-miR-25-3p | SOX2-OT |
| hsa-miR-125b-5p | AC004951.6 | hsa-miR-27b-3p | AC012368.1 | hsa-miR-378h | SOX2-OT |
| hsa-miR-148b-3p | ITCH-IT1 | hsa-miR-199a-5p | AC009506.1 | hsa-miR-378a-3p | SOX2-OT |
| hsa-miR-148a-3p | ITCH-IT1 | hsa-miR-143-3p | CTD-3222D19.12 | hsa-miR-125b-5p | RP11-90M2.5 |
| hsa-miR-186-5p | RP4-742J24.2 | hsa-miR-186-5p | CTD-3222D19.12 | hsa-miR-143-3p | SLC14A2-AS1 |
| hsa-miR-421 | RP11-282K24.3 | hsa-miR-34a-5p | AP001630.5 | hsa-miR-140-3p | SLC14A2-AS1 |
| hsa-miR-186-5p | RP11-282K24.3 | hsa-miR-143-3p | RP11-1018N14.5 | hsa-miR-148a-3p | SLC14A2-AS1 |
| hsa-miR-143-3p | RP11-282K24.3 | hsa-miR-27b-3p | RP11-1018N14.5 | hsa-miR-125b-5p | SLC14A2-AS1 |
| hsa-miR-374b-5p | RP11-282K24.3 | hsa-miR-27b-3p | IFT74-AS1 | hsa-miR-186-5p | AC005519.4 |
| hsa-miR-374b-5p | RP11-221J22.2 | hsa-miR-125b-5p | AP000350.5 | hsa-miR-34a-5p | CTA-305I2.1 |
| hsa-miR-148b-3p | RP11-221J22.2 | hsa-miR-143-3p | RP11-706O15.1 | hsa-miR-143-3p | RP11-250B2.6 |
| hsa-miR-146b-5p | RP11-358B23.5 | hsa-miR-27b-3p | AC002306.1 | hsa-miR-221-3p | RP11-250B2.6 |
| hsa-miR-374b-5p | RP11-16L21.7 | hsa-miR-27b-3p | CTD-2008P7.1 | hsa-miR-27b-3p | RP11-714G18.1 |
| hsa-miR-499a-5p | AC074286.1 | hsa-miR-34a-5p | RP5-983L19.2 | hsa-miR-421 | RP11-359K18.4 |
| hsa-miR-27b-3p | AC074286.1 | hsa-miR-34a-5p | RP11-923I11.4 | hsa-miR-143-3p | RP11-359K18.4 |
| hsa-miR-146b-5p | AC074286.1 | hsa-miR-374b-5p | CTC-327F10.5 | hsa-miR-199a-5p | RP11-151N17.1 |
| hsa-miR-499a-5p | RP11-53B2.3 | hsa-miR-25-3p | CTC-327F10.5 | hsa-miR-25-3p | RP11-678G15.1 |

| | | | | | |
|---|---|---|---|---|---|
| hsa-miR-26b-5p | RP11-439H8.4 | hsa-miR-27b-3p | RP11-19D2.2 | hsa-miR-125b-5p | RP11-574K11.29 |
| hsa-miR-27b-3p | RP11-439H8.4 | hsa-miR-125b-5p | RP11-19D2.2 | hsa-miR-26b-5p | RP11-574K11.29 |
| hsa-miR-125b-5p | RP11-428G5.5 | hsa-miR-186-5p | CTD-3138B18.6 | hsa-miR-499a-5p | RP11-574K11.29 |
| hsa-miR-186-5p | CTA-276F8.1 | hsa-miR-27b-3p | RP11-96A15.1 | hsa-miR-27b-3p | RP11-574K11.29 |
| hsa-miR-27b-3p | TMPRSS4-AS1 | hsa-miR-140-3p | RP11-529E10.7 | hsa-miR-143-3p | BDNF-AS |
| hsa-miR-140-3p | LINC00347 | hsa-miR-143-3p | PLS3-AS1 | hsa-miR-26b-5p | BDNF-AS |
| hsa-miR-199a-5p | LINC00347 | hsa-miR-27b-3p | LINC01226 | hsa-miR-421 | RP11-114H24.6 |
| hsa-miR-374b-5p | LINC00347 | hsa-miR-186-5p | CTD-2201E18.5 | hsa-miR-499a-5p | RP5-855D21.3 |
| hsa-miR-143-3p | RP11-89N17.3 | hsa-miR-27b-3p | ASTN2-AS1 | hsa-miR-186-5p | RP5-855D21.3 |
| hsa-miR-26b-5p | RP11-13N13.5 | hsa-miR-186-5p | LINC01415 | hsa-miR-143-3p | CTB-73N10.1 |
| hsa-miR-374b-5p | RP11-147L13.14 | hsa-miR-186-5p | MIR133A1HG | hsa-miR-374b-5p | RP11-466F5.10 |
| hsa-miR-421 | RP11-147L13.14 | hsa-miR-221-3p | MIR133A1HG | hsa-miR-125b-5p | KDM4A-AS1 |
| hsa-miR-143-3p | RP11-28G8.1 | hsa-miR-374b-5p | AC004053.1 | hsa-miR-27b-3p | AL133243.3 |
| hsa-miR-27b-3p | RP11-28G8.1 | hsa-miR-186-5p | AC004053.1 | hsa-miR-143-3p | RP4-635E18.7 |
| hsa-miR-143-3p | RP11-43N5.1 | hsa-miR-26b-5p | RP11-151A6.4 | hsa-miR-186-5p | LINC01422 |
| hsa-miR-499a-5p | RP11-713M15.1 | hsa-miR-421 | RP11-151A6.4 | hsa-miR-140-3p | LINC01422 |
| hsa-miR-27b-3p | RP11-274H2.3 | hsa-miR-146b-5p | RP11-151A6.4 | hsa-miR-25-3p | CTD-2278I10.1 |
| hsa-miR-34a-5p | RP11-404O13.1 | hsa-miR-25-3p | CTD-3162L10.1 | hsa-miR-378a-3p | PRSS51 |
| hsa-miR-499a-5p | CTB-113P19.4 | hsa-miR-125b-5p | CTD-3162L10.1 | hsa-miR-378h | PRSS51 |
| hsa-miR-148b-3p | PSMD6-AS2 | hsa-miR-378h | RP11-297C4.2 | hsa-miR-34a-5p | RP5-1042K10.13 |
| hsa-miR-186-5p | RP11-106M7.1 | hsa-miR-378a-3p | RP11-297C4.2 | hsa-miR-499a-5p | RP5-1042K10.13 |
| hsa-miR-27b-3p | RP11-106M7.1 | hsa-miR-374b-5p | RP3-413H6.2 | hsa-miR-186-5p | RP11-73M11.3 |
| hsa-miR-499a-5p | RP11-106M7.1 | hsa-miR-27b-3p | RP11-452C8.1 | hsa-miR-374b-5p | RP11-541P9.3 |
| hsa-miR-148b-3p | RP11-106M7.1 | hsa-miR-186-5p | LINC00525 | hsa-miR-143-3p | RP5-1027G4.3 |
| hsa-miR-148a-3p | RP11-106M7.1 | hsa-miR-26b-5p | CTD-2626G11.2 | hsa-miR-34a-5p | LINC00371 |
| hsa-miR-27b-3p | RP11-166B2.8 | hsa-miR-221-3p | CTD-2626G11.2 | hsa-miR-186-5p | LINC00371 |
| hsa-miR-125b-5p | RNASEH2B-AS1 | hsa-miR-27b-3p | RP11-448G15.3 | hsa-miR-421 | RP13-39P12.3 |
| hsa-miR-140-3p | RNASEH2B-AS1 | hsa-miR-148a-3p | RP5-1021I20.5 | hsa-miR-148b-3p | RP13-39P12.3 |

| | | | | | |
|---|---|---|---|---|---|
| hsa-miR-378h | RNASEH2B-AS1 | hsa-miR-148b-3p | RP5-1021I20.5 | hsa-miR-148a-3p | RP13-39P12.3 |
| hsa-miR-378a-3p | RNASEH2B-AS1 | hsa-miR-186-5p | AC097382.5 | hsa-miR-25-3p | RP11-982M15.8 |
| hsa-miR-26b-5p | RP11-817I4.2 | hsa-miR-27b-3p | TEX26-AS1 | hsa-miR-421 | CECR3 |
| hsa-miR-186-5p | RP11-817I4.2 | hsa-miR-125b-5p | TEX26-AS1 | hsa-miR-25-3p | CECR3 |
| hsa-miR-374b-5p | EPB41L4A-AS1 | hsa-miR-27b-3p | RP11-516C1.1 | hsa-miR-374b-5p | LINC01330 |
| hsa-miR-499a-5p | EPB41L4A-AS1 | hsa-miR-27b-3p | SPACA6P-AS | hsa-miR-125b-5p | LINC01330 |
| hsa-miR-374b-5p | PCGEM1 | hsa-miR-148b-3p | RP11-769O8.1 | hsa-miR-26b-5p | LINC01330 |
| hsa-miR-26b-5p | PCGEM1 | hsa-miR-143-3p | LINC01344 | hsa-miR-148b-3p | KIZ-AS1 |
| hsa-miR-143-3p | RP5-884M6.1 | hsa-miR-374b-5p | RNF139-AS1 | hsa-miR-148a-3p | KIZ-AS1 |
| hsa-miR-146b-5p | RP11-290O12.2 | hsa-miR-26b-5p | RNF139-AS1 | hsa-miR-186-5p | LINC00161 |
| hsa-miR-125b-5p | LINC00488 | hsa-miR-148a-3p | RP11-307C19.2 | hsa-miR-27b-3p | LINC00161 |
| hsa-miR-374b-5p | LINC00488 | hsa-miR-186-5p | DEPDC1-AS1 | hsa-miR-221-3p | RP5-984P4.6 |
| hsa-miR-499a-5p | LINC00488 | hsa-miR-146b-5p | EFCAB14-AS1 | hsa-miR-199a-5p | XXbac-B135H6.18 |
| hsa-miR-34a-5p | RP11-543D5.1 | hsa-miR-27b-3p | RP11-255M2.2 | hsa-miR-34a-5p | RP11-273G15.2 |
| hsa-miR-27b-3p | CTD-2265M8.2 | hsa-miR-148a-3p | RP11-196H14.2 | hsa-miR-148b-3p | RP11-273G15.2 |
| hsa-miR-27b-3p | DLX6-AS1 | hsa-miR-148b-3p | RP11-196H14.2 | hsa-miR-199a-5p | RP11-273G15.2 |
| hsa-miR-26b-5p | DLX6-AS1 | hsa-miR-34a-5p | RP5-858L17.1 | hsa-miR-148a-3p | RP11-273G15.2 |
| hsa-miR-186-5p | DLX6-AS1 | hsa-miR-221-3p | LINC01093 | hsa-miR-34a-5p | CTB-39G8.3 |
| hsa-miR-374b-5p | DLX6-AS1 | hsa-miR-34a-5p | CTC-472C24.1 | hsa-miR-143-3p | NARF-IT1 |
| hsa-miR-421 | DLX6-AS1 | hsa-miR-143-3p | RP11-415J8.3 | hsa-miR-125b-5p | NARF-IT1 |
| hsa-miR-125b-5p | DLX6-AS1 | hsa-miR-199a-5p | RP11-415J8.3 | hsa-miR-25-3p | RP11-672L10.6 |
| hsa-miR-143-3p | DLX6-AS1 | hsa-miR-146b-5p | LUCAT1 | hsa-miR-125b-5p | RP11-672L10.6 |
| hsa-miR-143-3p | CTD-2002H8.2 | hsa-miR-421 | LUCAT1 | hsa-miR-374b-5p | ITPK1-AS1 |
| hsa-miR-378a-3p | CTD-2616J11.16 | hsa-miR-199a-5p | LUCAT1 | hsa-miR-34a-5p | ITPK1-AS1 |
| hsa-miR-378h | CTD-2616J11.16 | hsa-miR-186-5p | LUCAT1 | hsa-miR-26b-5p | CTC-471C19.1 |
| hsa-miR-34a-5p | RP11-12A2.3 | hsa-miR-378h | YEATS2-AS1 | hsa-miR-27b-3p | CTC-471C19.1 |
| hsa-miR-25-3p | RP11-12A2.3 | hsa-miR-378a-3p | YEATS2-AS1 | hsa-miR-186-5p | CTC-471C19.1 |

| | | | | | |
|---|---|---|---|---|---|
| hsa-miR-148b-3p | RP11-127B20.2 | hsa-miR-34a-5p | YEATS2-AS1 | hsa-miR-199a-5p | RP11-132M7.3 |
| hsa-miR-148a-3p | RP11-127B20.2 | hsa-miR-146b-5p | CTD-2501M5.1 | hsa-miR-186-5p | RP11-132M7.3 |
| hsa-miR-221-3p | RP11-127B20.2 | hsa-miR-34a-5p | CTD-2501M5.1 | hsa-miR-421 | RP11-549L6.3 |
| hsa-miR-374b-5p | RP11-127B20.2 | hsa-miR-199a-5p | AC012613.2 | hsa-miR-27b-3p | RP4-778K6.3 |
| hsa-miR-186-5p | LL0XNC01-7P3.1 | hsa-miR-34a-5p | AC012613.2 | hsa-miR-25-3p | CTC-250I14.3 |
| hsa-miR-125b-5p | LL0XNC01-7P3.1 | hsa-miR-143-3p | RP11-166B2.5 | hsa-miR-34a-5p | CADM3-AS1 |
| hsa-miR-148a-3p | LL0XNC01-7P3.1 | hsa-miR-27b-3p | AC008088.4 | hsa-miR-125b-5p | CADM3-AS1 |
| hsa-miR-421 | LL0XNC01-7P3.1 | hsa-miR-148b-3p | AC008088.4 | hsa-miR-378h | RP11-509A17.3 |
| hsa-miR-148b-3p | LL0XNC01-7P3.1 | hsa-miR-148a-3p | AC008088.4 | hsa-miR-378a-3p | RP11-509A17.3 |
| hsa-miR-34a-5p | RP11-638I2.4 | hsa-miR-374b-5p | LINC00969 | hsa-miR-199a-5p | RP11-509A17.3 |
| hsa-miR-146b-5p | CTD-2170G1.2 | hsa-miR-34a-5p | LINC00969 | hsa-miR-125b-5p | RP11-509A17.3 |
| hsa-miR-143-3p | CTD-2170G1.2 | hsa-miR-148b-3p | RP11-872J21.3 | hsa-miR-25-3p | RP11-509A17.3 |
| hsa-miR-148b-3p | CTD-2170G1.2 | hsa-miR-26b-5p | CTD-2085J24.3 | hsa-miR-140-3p | AC012314.8 |
| hsa-miR-148a-3p | CTD-2170G1.2 | hsa-miR-27b-3p | RP11-351J23.2 | hsa-miR-374b-5p | RP11-504P24.9 |
| hsa-miR-34a-5p | CTD-2170G1.2 | hsa-miR-34a-5p | RP11-64B16.4 | hsa-miR-146b-5p | RP11-1E4.1 |
| hsa-miR-499a-5p | RP11-718O11.1 | hsa-miR-421 | RP11-981G7.6 | hsa-miR-143-3p | RP11-148K1.12 |
| hsa-miR-374b-5p | RP11-415I12.3 | hsa-miR-148b-3p | RP11-981G7.6 | hsa-miR-374b-5p | RP11-33B1.3 |
| hsa-miR-374b-5p | RP11-300G22.2 | hsa-miR-148a-3p | RP11-981G7.6 | hsa-miR-421 | RP11-278L15.2 |
| hsa-miR-27b-3p | RP11-66B24.4 | hsa-miR-125b-5p | CASK-AS1 | hsa-miR-186-5p | RP11-278L15.2 |
| hsa-miR-25-3p | RP11-66B24.4 | hsa-miR-34a-5p | LINC01285 | hsa-miR-374b-5p | AC005614.3 |
| hsa-miR-199a-5p | RP11-66B24.4 | hsa-miR-125b-5p | AF196972.9 | hsa-miR-186-5p | AC005614.3 |
| hsa-miR-125b-5p | RP11-66B24.4 | hsa-miR-146b-5p | RP11-565A3.2 | hsa-miR-25-3p | RP11-490B18.6 |
| hsa-miR-186-5p | CTC-523E23.5 | hsa-miR-378h | RP11-565A3.2 | hsa-miR-221-3p | RP11-55K13.1 |

| | | | | | |
|---|---|---|---|---|---|
| hsa-miR-27b-3p | CTC-523E23.5 | hsa-miR-378a-3p | RP11-565A3.2 | hsa-miR-499a-5p | RP11-28F1.2 |
| hsa-miR-125b-5p | RP11-197N18.7 | hsa-miR-27b-3p | RP11-1391J7.1 | hsa-miR-140-3p | RP11-317M11.1 |
| hsa-miR-186-5p | CTD-2282P23.2 | hsa-miR-140-3p | CTD-2184D3.5 | hsa-miR-143-3p | RP11-122C5.3 |
| hsa-miR-199a-5p | AC139100.3 | hsa-miR-125b-5p | CTD-2184D3.5 | hsa-miR-186-5p | LINC00202-1 |
| hsa-miR-140-3p | AC139100.3 | hsa-miR-421 | RP11-504P24.8 | hsa-miR-378h | LINC00202-1 |
| hsa-miR-374b-5p | CH17-262A2.1 | hsa-miR-146b-5p | RP11-504P24.8 | hsa-miR-378a-3p | LINC00202-1 |
| hsa-miR-143-3p | RP11-728E14.3 | hsa-miR-186-5p | RP11-504P24.8 | hsa-miR-125b-5p | RP11-363E6.4 |
| hsa-miR-27b-3p | RP11-728E14.3 | hsa-miR-378h | RP11-504P24.8 | hsa-miR-421 | RP11-667M19.2 |
| hsa-miR-146b-5p | RP11-728E14.3 | hsa-miR-140-3p | AKT3-IT1 | hsa-miR-125b-5p | RP11-768B22.2 |
| hsa-miR-421 | RP11-728E14.3 | hsa-miR-199a-5p | RP11-573D15.8 | hsa-miR-499a-5p | RP11-768B22.2 |
| hsa-miR-26b-5p | RP11-728E14.3 | hsa-miR-143-3p | RP11-573D15.8 | hsa-miR-374b-5p | RP11-77E14.2 |
| hsa-miR-374b-5p | RP11-728E14.3 | hsa-miR-374b-5p | AC005754.8 | hsa-miR-140-3p | RP3-402G11.27 |
| hsa-miR-499a-5p | RP11-728E14.3 | hsa-miR-186-5p | AC005754.8 | hsa-miR-125b-5p | RP11-181G12.4 |
| hsa-miR-143-3p | RP11-66A2.2 | hsa-miR-34a-5p | AC005754.8 | hsa-miR-146b-5p | DGCR5 |
| hsa-miR-34a-5p | RP11-856M7.1 | hsa-miR-148b-3p | RP11-297C4.1 | hsa-miR-186-5p | RP11-50I19.2 |
| hsa-miR-374b-5p | AC090616.2 | hsa-miR-378h | RP11-480D4.2 | hsa-miR-146b-5p | RP11-326E22.1 |
| hsa-miR-199a-5p | AP000345.1 | hsa-miR-378a-3p | RP11-480D4.2 | hsa-miR-143-3p | RP1-78B3.1 |
| hsa-miR-140-3p | RP11-344P13.4 | hsa-miR-125b-5p | RP11-285G1.2 | hsa-miR-374b-5p | RP1-78B3.1 |
| hsa-miR-125b-5p | AC124997.1 | hsa-miR-125b-5p | PRR7-AS1 | hsa-miR-221-3p | RP11-513G11.2 |
| hsa-miR-143-3p | CTD-2313J17.1 | hsa-miR-125b-5p | RP11-709D24.6 | hsa-miR-421 | RP3-467K16.4 |
| hsa-miR-499a-5p | AC003104.1 | hsa-miR-125b-5p | RP11-473C18.3 | hsa-miR-34a-5p | RP3-467K16.4 |
| hsa-miR-374b-5p | AC003104.1 | hsa-miR-378h | RP11-18H21.1 | hsa-miR-499a-5p | RP11-739N10.1 |
| hsa-miR-140-3p | AF127936.3 | hsa-miR-143-3p | RP11-18H21.1 | hsa-miR-199a-5p | RP11-739N10.1 |
| hsa-miR-27b-3p | UBAC2-AS1 | hsa-miR-378a-3p | RP11-18H21.1 | hsa-miR-34a-5p | RP11-739N10.1 |
| hsa-miR-27b-3p | RP11-734K21.5 | hsa-miR-26b-5p | U82695.9 | hsa-miR-148a-3p | RP11-739N10.1 |
| hsa-miR-199a-5p | RPL34-AS1 | hsa-miR-27b-3p | RP5-836J3.1 | hsa-miR-148b-3p | RP11-739N10.1 |
| hsa-miR-374b-5p | RPL34-AS1 | hsa-miR-186-5p | RP11-706O15.3 | hsa-miR-421 | RP11-474P2.5 |
| hsa-miR-499a-5p | RPL34-AS1 | hsa-miR-186-5p | AF131217.1 | hsa-miR-146b-5p | RP11-307C12.13 |

| | | | | | |
|---|---|---|---|---|---|
| hsa-miR-25-3p | RPL34-AS1 | hsa-miR-146b-5p | AF131217.1 | hsa-miR-148a-3p | RP11-78I14.1 |
| hsa-miR-186-5p | RP11-660M5.1 | hsa-miR-27b-3p | AF131217.1 | hsa-miR-148b-3p | RP11-78I14.1 |
| hsa-miR-25-3p | RP1-79C4.1 | hsa-miR-499a-5p | AF131217.1 | hsa-miR-26b-5p | H1FX-AS1 |
| hsa-miR-221-3p | RP1-79C4.1 | hsa-miR-146b-5p | RP11-538D16.2 | hsa-miR-143-3p | RP3-325F22.3 |
| hsa-miR-146b-5p | RP1-79C4.1 | hsa-miR-421 | RP11-173C1.1 | hsa-miR-148b-3p | RP11-459I19.1 |
| hsa-miR-146b-5p | RP11-61L19.3 | hsa-miR-25-3p | RP11-167J8.3 | hsa-miR-148a-3p | RP11-459I19.1 |
| hsa-miR-421 | RP11-486G15.2 | hsa-miR-143-3p | RP11-553A21.3 | hsa-miR-146b-5p | FLJ22763 |
| hsa-miR-25-3p | RP11-717D12.1 | hsa-miR-143-3p | MIR4435-1HG | hsa-miR-221-3p | FLJ22763 |
| hsa-miR-146b-5p | LINC01583 | hsa-miR-378a-3p | MIR4435-1HG | hsa-miR-199a-5p | FLJ22763 |
| hsa-miR-125b-5p | LINC01583 | hsa-miR-378h | MIR4435-1HG | hsa-miR-186-5p | FLJ22763 |
| hsa-miR-27b-3p | GS1-5L10.1 | hsa-miR-421 | MIR4435-1HG | hsa-miR-143-3p | FAM170B-AS1 |
| hsa-miR-143-3p | AC011752.1 | hsa-miR-27b-3p | RP11-266O8.1 | hsa-miR-26b-5p | FAM170B-AS1 |
| hsa-miR-27b-3p | RP3-405J10.3 | hsa-miR-125b-5p | RP11-266O8.1 | hsa-miR-148a-3p | RP11-538I12.3 |
| hsa-miR-148b-3p | RP3-405J10.3 | hsa-miR-199a-5p | RP11-266O8.1 | hsa-miR-27b-3p | KCNMA1-AS3 |
| hsa-miR-143-3p | RP3-405J10.3 | hsa-miR-143-3p | CTD-2609K8.3 | hsa-miR-186-5p | RP3-395M20.8 |
| hsa-miR-374b-5p | RP11-174G6.5 | hsa-miR-27b-3p | RP4-798A10.7 | hsa-miR-378a-3p | RP3-395M20.8 |
| hsa-miR-186-5p | RP11-174G6.5 | hsa-miR-374b-5p | RP4-798A10.7 | hsa-miR-378h | RP3-395M20.8 |
| hsa-miR-199a-5p | LINC01483 | hsa-miR-25-3p | AC019117.2 | hsa-miR-125b-5p | RP3-395M20.8 |
| hsa-miR-143-3p | LINC01483 | hsa-miR-34a-5p | DIAPH2-AS1 | hsa-miR-374b-5p | ACAP2-IT1 |
| hsa-miR-374b-5p | RP11-73M18.6 | hsa-miR-146b-5p | CTD-2621I17.6 | hsa-miR-186-5p | RP11-189E14.5 |
| hsa-miR-146b-5p | CLIP1-AS1 | hsa-miR-27b-3p | CTD-2621I17.6 | hsa-miR-26b-5p | MIR181A1HG |
| hsa-miR-25-3p | RP11-115J16.1 | hsa-miR-27b-3p | RP11-284N8.3 | hsa-miR-143-3p | RP11-173B14.4 |
| hsa-miR-146b-5p | RP11-115J16.1 | hsa-miR-34a-5p | RP11-284N8.3 | hsa-miR-25-3p | RP11-167B3.3 |
| hsa-miR-186-5p | CTC-339O9.1 | hsa-miR-146b-5p | RP11-284N8.3 | hsa-miR-186-5p | AC006159.4 |
| hsa-miR-186-5p | RP11-44F14.2 | hsa-miR-26b-5p | RP11-284N8.3 | hsa-miR-186-5p | DAPK1-IT1 |
| hsa-miR-378h | RP11-632K5.3 | hsa-miR-186-5p | RP11-284N8.3 | hsa-miR-25-3p | DAPK1-IT1 |
| hsa-miR-378a-3p | RP11-632K5.3 | hsa-miR-27b-3p | AC114812.8 | hsa-miR-27b-3p | RP11-356O9.2 |
| hsa-miR-186-5p | CTB-176F20.3 | hsa-miR-26b-5p | AC091153.4 | hsa-miR-186-5p | RP11-356O9.2 |

| | | | | | |
|---|---|---|---|---|---|
| hsa-miR-421 | CTB-176F20.3 | hsa-miR-499a-5p | RP11-95G17.2 | hsa-miR-421 | RP11-513G19.1 |
| hsa-miR-26b-5p | RP3-355L5.4 | hsa-miR-26b-5p | CTB-46B19.2 | hsa-miR-26b-5p | RP11-256L6.3 |
| hsa-miR-27b-3p | CTB-167B5.1 | hsa-miR-374b-5p | AIRN | hsa-miR-148a-3p | HOTTIP |
| hsa-miR-146b-5p | RP11-370B11.3 | hsa-miR-186-5p | RP11-680G24.5 | hsa-miR-148b-3p | HOTTIP |
| hsa-miR-146b-5p | CTD-2540L5.6 | hsa-miR-186-5p | XIST | hsa-miR-143-3p | HOTTIP |
| hsa-miR-34a-5p | RP11-210M15.2 | hsa-miR-374b-5p | XIST | hsa-miR-125b-5p | RP11-77H9.2 |
| hsa-miR-148b-3p | RP11-311F12.1 | hsa-miR-499a-5p | XIST | hsa-miR-125b-5p | C1RL-AS1 |
| hsa-miR-148a-3p | RP11-311F12.1 | hsa-miR-421 | XIST | hsa-miR-146b-5p | AC008269.2 |
| hsa-miR-34a-5p | RP11-311F12.1 | hsa-miR-34a-5p | XIST | hsa-miR-378a-3p | CTD-2528A14.3 |
| hsa-miR-125b-5p | RP11-311F12.1 | hsa-miR-140-3p | XIST | hsa-miR-378h | CTD-2528A14.3 |
| hsa-miR-186-5p | RP11-474D1.3 | hsa-miR-25-3p | XIST | hsa-miR-125b-5p | RP11-20D14.6 |
| hsa-miR-125b-5p | RP11-474D1.3 | hsa-miR-27b-3p | XIST | hsa-miR-186-5p | RP11-20D14.6 |
| hsa-miR-27b-3p | RP11-474D1.3 | hsa-miR-146b-5p | XIST | hsa-miR-25-3p | RP11-20D14.6 |
| hsa-miR-25-3p | RP11-90P5.5 | hsa-miR-148a-3p | XIST | hsa-miR-199a-5p | RP11-20D14.6 |
| hsa-miR-186-5p | RP11-142O6.1 | hsa-miR-378a-3p | CTD-2554C21.2 | hsa-miR-27b-3p | RP11-290F5.2 |
| hsa-miR-221-3p | RP11-142O6.1 | hsa-miR-378h | CTD-2554C21.2 | hsa-miR-143-3p | LINC00298 |
| hsa-miR-499a-5p | RP11-142O6.1 | hsa-miR-125b-5p | RP11-430C7.5 | hsa-miR-27b-3p | LINC00298 |
| hsa-miR-27b-3p | RP11-45A17.2 | hsa-miR-34a-5p | RP11-10L7.1 | hsa-miR-143-3p | AC006460.2 |
| hsa-miR-26b-5p | AC007365.1 | hsa-miR-26b-5p | RP11-10L7.1 | hsa-miR-27b-3p | RP11-15I11.3 |
| hsa-miR-199a-5p | AC007365.1 | hsa-miR-140-3p | RP11-389O22.1 | hsa-miR-26b-5p | DNAJC3-AS1 |
| hsa-miR-146b-5p | COL4A2-AS1 | hsa-miR-143-3p | SH3BP5-AS1 | hsa-miR-125b-5p | DNAJC3-AS1 |
| hsa-miR-199a-5p | RP11-53B2.2 | hsa-miR-25-3p | RP11-11N9.4 | hsa-miR-140-3p | DNAJC3-AS1 |
| hsa-miR-143-3p | KB-1674E1.1 | hsa-miR-146b-5p | CTD-2143L24.1 | hsa-miR-34a-5p | RP11-247I13.11 |
| hsa-miR-374b-5p | KB-1674E1.1 | hsa-miR-221-3p | CTD-2143L24.1 | hsa-miR-499a-5p | CTD-3116E22.8 |
| hsa-miR-186-5p | KB-1674E1.1 | hsa-miR-374b-5p | RP11-554D15.1 | hsa-miR-140-3p | CTD-3116E22.8 |
| hsa-miR-26b-5p | DPH6-AS1 | hsa-miR-421 | RP11-46E17.8 | hsa-miR-374b-5p | CTD-3116E22.8 |
| hsa-miR-27b-3p | DPH6-AS1 | hsa-miR-27b-3p | RP11-46E17.8 | hsa-miR-186-5p | RP11-1060J15.9 |
| hsa-miR-140-3p | DPH6-AS1 | hsa-miR-378a-3p | RP11-46E17.8 | hsa-miR-186-5p | AC015971.2 |

| | | | | | |
|---|---|---|---|---|---|
| hsa-miR-26b-5p | LINC00427 | hsa-miR-378h | RP11-46E17.8 | hsa-miR-27b-3p | C14orf132 |
| hsa-miR-186-5p | ENTPD3-AS1 | hsa-miR-199a-5p | RP11-46E17.8 | hsa-miR-186-5p | C14orf132 |
| hsa-miR-148a-3p | EDRF1-AS1 | hsa-miR-186-5p | RP11-46E17.8 | hsa-miR-34a-5p | RP11-744I24.3 |
| hsa-miR-148b-3p | EDRF1-AS1 | hsa-miR-27b-3p | RP4-569D19.8 | hsa-miR-26b-5p | AP000230.1 |
| hsa-miR-374b-5p | AC105760.3 | hsa-miR-27b-3p | RP11-95O2.5 | hsa-miR-186-5p | AP000230.1 |
| hsa-miR-199a-5p | RP11-2O17.2 | hsa-miR-146b-5p | RP11-95O2.5 | hsa-miR-140-3p | LINC01398 |
| hsa-miR-143-3p | RP11-661C3.2 | hsa-miR-374b-5p | RP11-258C19.7 | hsa-miR-143-3p | LINC01337 |
| hsa-miR-26b-5p | CTD-3195I5.3 | hsa-miR-140-3p | LINC01585 | hsa-miR-34a-5p | RP11-197K3.1 |
| hsa-miR-125b-5p | CTD-3195I5.3 | hsa-miR-125b-5p | AC018816.3 | hsa-miR-140-3p | AC011893.3 |
| hsa-miR-140-3p | RP11-145M4.2 | hsa-miR-374b-5p | RP11-268P4.6 | hsa-miR-125b-5p | C1orf147 |
| hsa-miR-125b-5p | AC007966.1 | hsa-miR-26b-5p | ITPKB-IT1 | hsa-miR-140-3p | CDC42-IT1 |
| hsa-miR-374b-5p | RP5-1092A3.4 | hsa-miR-199a-5p | RP11-386J22.3 | hsa-miR-186-5p | CDC42-IT1 |
| hsa-miR-186-5p | RP11-626G11.4 | hsa-miR-125b-5p | RP11-386J22.3 | hsa-miR-146b-5p | LINC01094 |
| hsa-miR-186-5p | RP3-394A18.1 | hsa-miR-186-5p | RP11-386J22.3 | hsa-miR-221-3p | KB-1507C5.3 |
| hsa-miR-34a-5p | RP3-394A18.1 | hsa-miR-140-3p | RP11-386J22.3 | hsa-miR-125b-5p | RP11-348J12.2 |
| hsa-miR-26b-5p | RP3-394A18.1 | hsa-miR-378h | AC012370.3 | hsa-miR-125b-5p | RP11-421P23.2 |
| hsa-miR-143-3p | RP3-394A18.1 | hsa-miR-378a-3p | AC012370.3 | hsa-miR-499a-5p | RP11-474G23.3 |
| hsa-miR-148a-3p | RP3-394A18.1 | hsa-miR-221-3p | AC012370.3 | hsa-miR-140-3p | SLC6A1-AS1 |
| hsa-miR-199a-5p | RP3-394A18.1 | hsa-miR-26b-5p | RP11-554A11.4 | hsa-miR-125b-5p | RP11-640N11.2 |
| hsa-miR-148b-3p | RP3-394A18.1 | hsa-miR-421 | RP11-554A11.4 | hsa-miR-143-3p | RP11-640N11.2 |
| hsa-miR-27b-3p | RP3-394A18.1 | hsa-miR-499a-5p | RP3-368A4.6 | hsa-miR-186-5p | RP11-277A4.4 |
| hsa-miR-25-3p | RP3-394A18.1 | hsa-miR-374b-5p | RP3-368A4.6 | hsa-miR-143-3p | AC009120.5 |
| hsa-miR-374b-5p | RP3-394A18.1 | hsa-miR-421 | RP3-368A4.6 | hsa-miR-34a-5p | AC009120.5 |
| hsa-miR-378h | RP3-394A18.1 | hsa-miR-186-5p | RP3-368A4.6 | hsa-miR-26b-5p | PRKCA-AS1 |
| hsa-miR-146b-5p | RP3-394A18.1 | hsa-miR-27b-3p | RP3-368A4.6 | hsa-miR-186-5p | PRKCA-AS1 |
| hsa-miR-125b-5p | RP3-394A18.1 | hsa-miR-143-3p | LINC01447 | hsa-miR-374b-5p | RP11-493E12.2 |
| hsa-miR-221-3p | RP3-394A18.1 | hsa-miR-125b-5p | LINC01447 | hsa-miR-186-5p | CTC-506B8.1 |
| hsa-miR-378a-3p | RP3-394A18.1 | hsa-miR-34a-5p | AC087501.1 | hsa-miR-143-3p | SSBP3-AS1 |

| | | | | | |
|---|---|---|---|---|---|
| hsa-miR-499a-5p | RP3-394A18.1 | hsa-miR-378h | RP11-22A3.2 | hsa-miR-27b-3p | AP000797.4 |
| hsa-miR-378h | RP11-534L20.5 | hsa-miR-26b-5p | RP11-22A3.2 | hsa-miR-378a-3p | CTD-2528A14.5 |
| hsa-miR-378a-3p | RP11-534L20.5 | hsa-miR-378a-3p | RP11-22A3.2 | hsa-miR-378h | CTD-2528A14.5 |
| hsa-miR-27b-3p | RP1-63G5.7 | hsa-miR-221-3p | AC000120.7 | hsa-miR-421 | LINC00862 |
| hsa-miR-34a-5p | RP11-285J16.1 | hsa-miR-26b-5p | AC000120.7 | hsa-miR-143-3p | CCDC144NL-AS1 |
| hsa-miR-186-5p | RP11-285J16.1 | hsa-miR-143-3p | CTB-12O2.1 | hsa-miR-26b-5p | CCDC144NL-AS1 |
| hsa-miR-148b-3p | MORF4L2-AS1 | hsa-miR-378a-3p | CTB-12O2.1 | hsa-miR-27b-3p | CCDC144NL-AS1 |
| hsa-miR-148a-3p | MORF4L2-AS1 | hsa-miR-378h | CTB-12O2.1 | hsa-miR-186-5p | CCDC144NL-AS1 |
| hsa-miR-374b-5p | RP11-52A20.2 | hsa-miR-186-5p | BX255923.3 | hsa-miR-34a-5p | CCDC144NL-AS1 |
| hsa-miR-499a-5p | AC009501.4 | hsa-miR-140-3p | XXbac-BPGBPG55C20.1 | hsa-miR-140-3p | FSIP2-AS1 |
| hsa-miR-499a-5p | MALAT1 | hsa-miR-27b-3p | RP11-799B12.4 | hsa-miR-34a-5p | LINC00900 |
| hsa-miR-26b-5p | MALAT1 | hsa-miR-148a-3p | RP11-798G7.8 | hsa-miR-148a-3p | AC092168.2 |
| hsa-miR-143-3p | MALAT1 | hsa-miR-148b-3p | RP11-798G7.8 | hsa-miR-421 | SLC8A1-AS1 |
| hsa-miR-186-5p | MALAT1 | hsa-miR-499a-5p | RP11-798G7.8 | hsa-miR-26b-5p | SLC8A1-AS1 |
| hsa-miR-146b-5p | AC004485.3 | hsa-miR-378a-3p | RP11-88G17.6 | hsa-miR-27b-3p | SLC8A1-AS1 |
| hsa-miR-186-5p | DLEU2 | hsa-miR-378h | RP11-88G17.6 | hsa-miR-27b-3p | RP1-300G12.2 |
| hsa-miR-374b-5p | RP11-234K19.1 | hsa-miR-186-5p | RP11-20J15.3 | hsa-miR-143-3p | RP11-13P5.2 |
| hsa-miR-199a-5p | RP11-415D17.4 | hsa-miR-26b-5p | RP11-20J15.3 | hsa-miR-146b-5p | RP11-328C8.4 |
| hsa-miR-34a-5p | RP11-818F20.5 | hsa-miR-125b-5p | RP11-20J15.3 | hsa-miR-199a-5p | AC005042.4 |
| hsa-miR-378a-3p | CTA-398F10.2 | hsa-miR-34a-5p | RP11-496I9.1 | hsa-miR-186-5p | RP11-70D24.4 |
| hsa-miR-378h | CTA-398F10.2 | hsa-miR-26b-5p | RP11-325L12.7 | hsa-miR-186-5p | RP11-379B18.6 |
| hsa-miR-125b-5p | CTD-2616J11.3 | hsa-miR-140-3p | RP1-10C16.1 | hsa-miR-125b-5p | RP11-23N2.4 |

| | | | | | |
|---|---|---|---|---|---|
| hsa-miR-146b-5p | RP11-321G12.1 | hsa-miR-125b-5p | AP000289.6 | hsa-miR-27b-3p | LINC01085 |
| hsa-miR-125b-5p | PLCB2-AS1 | hsa-miR-27b-3p | AC007879.2 | hsa-miR-143-3p | LINC01085 |
| hsa-miR-186-5p | RP11-377D9.3 | hsa-miR-378h | CTD-3080P12.3 | hsa-miR-186-5p | RP11-932O9.9 |
| hsa-miR-27b-3p | AC093642.3 | hsa-miR-378a-3p | CTD-3080P12.3 | hsa-miR-421 | RP11-946P6.6 |
| hsa-miR-26b-5p | AC093642.3 | hsa-miR-421 | CTD-3080P12.3 | hsa-miR-143-3p | RP11-742B18.1 |
| hsa-miR-146b-5p | AFAP1-AS1 | hsa-miR-146b-5p | AC137932.6 | hsa-miR-27b-3p | RP11-742B18.1 |
| hsa-miR-421 | AFAP1-AS1 | hsa-miR-499a-5p | RP1-225E12.2 | hsa-miR-374b-5p | KCNMB2-AS1 |
| hsa-miR-186-5p | AFAP1-AS1 | hsa-miR-125b-5p | RP11-70J12.1 | hsa-miR-27b-3p | KCNMB2-AS1 |
| hsa-miR-378a-3p | RP11-676J12.4 | hsa-miR-421 | RP11-166N17.1 | hsa-miR-26b-5p | RP11-295H24.5 |
| hsa-miR-378h | RP11-676J12.4 | hsa-miR-146b-5p | RP11-13N12.1 | hsa-miR-378h | RP11-496N12.6 |
| hsa-miR-221-3p | LINC01215 | hsa-miR-148b-3p | RP11-141C7.4 | hsa-miR-378a-3p | RP11-496N12.6 |
| hsa-miR-27b-3p | LINC01215 | hsa-miR-148a-3p | RP11-141C7.4 | hsa-miR-146b-5p | RP11-214O1.3 |
| hsa-miR-34a-5p | AC092535.3 | hsa-miR-146b-5p | RP11-141C7.4 | hsa-miR-25-3p | AC007308.6 |
| hsa-miR-140-3p | RP13-578N3.3 | hsa-miR-25-3p | RP11-141C7.4 | hsa-miR-125b-5p | AC007308.6 |
| hsa-miR-27b-3p | RP11-221N13.3 | hsa-miR-186-5p | RP11-141C7.4 | hsa-miR-186-5p | RABGAP1L-IT1 |
| hsa-miR-125b-5p | RP11-221N13.3 | hsa-miR-143-3p | SND1-IT1 | hsa-miR-125b-5p | RP11-247A12.7 |
| hsa-miR-186-5p | LINC01555 | hsa-miR-421 | AC006145.4 | hsa-miR-27b-3p | RP11-486O13.4 |
| hsa-miR-25-3p | RP5-956O18.2 | hsa-miR-499a-5p | AC006145.4 | hsa-miR-26b-5p | RP11-246A10.1 |
| hsa-miR-421 | RP11-30L3.2 | hsa-miR-499a-5p | RP11-727F15.13 | hsa-miR-146b-5p | GS1-279B7.1 |
| hsa-miR-146b-5p | RP5-933B4.1 | hsa-miR-374b-5p | RP11-286E11.1 | hsa-miR-146b-5p | RP11-324E6.10 |
| hsa-miR-143-3p | AC016683.5 | hsa-miR-140-3p | RP11-351J23.1 | hsa-miR-146b-5p | GRM5-AS1 |
| hsa-miR-26b-5p | RP11-297N6.4 | hsa-miR-221-3p | RP11-351J23.1 | hsa-miR-148b-3p | PAQR9-AS1 |
| hsa-miR-140-3p | RP11-64D24.2 | hsa-miR-125b-5p | RP11-114F10.3 | hsa-miR-378h | PAQR9-AS1 |
| hsa-miR-143-3p | RP1-35C21.2 | hsa-miR-34a-5p | AC006116.19 | hsa-miR-378a-3p | PAQR9-AS1 |
| hsa-miR-125b-5p | MIR497HG | hsa-miR-34a-5p | LAMA5-AS1 | hsa-miR-199a-5p | PAQR9-AS1 |
| hsa-miR-186-5p | RP11-214K3.20 | hsa-miR-186-5p | LINC00540 | hsa-miR-378h | LINC00505 |
| hsa-miR-499a-5p | RP11-411B10.2 | hsa-miR-374b-5p | RP11-111M22.4 | hsa-miR-378a-3p | LINC00505 |
| hsa-miR-374b-5p | RP11-411B10.2 | hsa-miR-34a-5p | TNK2-AS1 | hsa-miR-27b-3p | RP11-354E11.2 |

| | | | | | |
|---|---|---|---|---|---|
| hsa-miR-125b-5p | SRRM2-AS1 | hsa-miR-148b-3p | LINC00894 | hsa-miR-499a-5p | RP11-354E11.2 |
| hsa-miR-148b-3p | RP11-548L20.1 | hsa-miR-148a-3p | LINC00894 | hsa-miR-34a-5p | CTD-2600O9.2 |
| hsa-miR-148a-3p | RP11-548L20.1 | hsa-miR-374b-5p | RP11-645C24.5 | hsa-miR-148b-3p | RP11-785G17.1 |
| hsa-miR-186-5p | RP11-212I21.4 | hsa-miR-26b-5p | RP11-433C9.2 | hsa-miR-26b-5p | RP11-159H10.3 |
| hsa-miR-374b-5p | RP11-212I21.4 | hsa-miR-499a-5p | RP11-674P19.2 | hsa-miR-143-3p | RP11-449J21.5 |
| hsa-miR-125b-5p | LINC00843 | hsa-miR-26b-5p | RP11-674P19.2 | hsa-miR-26b-5p | DLEU7-AS1 |
| hsa-miR-26b-5p | LINC00843 | hsa-miR-25-3p | RP11-674P19.2 | hsa-miR-140-3p | LINC00486 |
| hsa-miR-499a-5p | RP11-57H12.5 | hsa-miR-140-3p | RP11-674P19.2 | hsa-miR-148a-3p | LINC00486 |
| hsa-miR-125b-5p | RP11-260E18.1 | hsa-miR-374b-5p | RP11-674P19.2 | hsa-miR-26b-5p | ZNF793-AS1 |
| hsa-miR-186-5p | RP11-1D12.2 | hsa-miR-199a-5p | RP11-758M4.4 | hsa-miR-148b-3p | LINC01539 |
| hsa-miR-199a-5p | RP11-480C16.1 | hsa-miR-374b-5p | RP11-758M4.4 | hsa-miR-148a-3p | LINC01539 |
| hsa-miR-27b-3p | RP11-480C16.1 | hsa-miR-34a-5p | RP1-122P22.4 | hsa-miR-186-5p | LINC01539 |
| hsa-miR-374b-5p | RP11-480C16.1 | hsa-miR-34a-5p | RP11-615I2.6 | hsa-miR-143-3p | LINC01539 |
| hsa-miR-374b-5p | RP11-932O9.10 | hsa-miR-125b-5p | AC004448.5 | hsa-miR-148a-3p | RP11-141O11.2 |
| hsa-miR-146b-5p | RP11-932O9.10 | hsa-miR-143-3p | AC004448.5 | hsa-miR-148b-3p | RP11-141O11.2 |
| hsa-miR-186-5p | AP001628.7 | hsa-miR-34a-5p | AC004448.5 | hsa-miR-34a-5p | RP4-539M6.14 |
| hsa-miR-27b-3p | RP11-10C8.2 | hsa-miR-378h | AC004448.5 | hsa-miR-146b-5p | AC015849.16 |
| hsa-miR-199a-5p | RP1-5O6.4 | hsa-miR-378a-3p | AC004448.5 | hsa-miR-378h | AC015849.16 |
| hsa-miR-146b-5p | MIR143HG | hsa-miR-26b-5p | MEG9 | hsa-miR-378a-3p | AC015849.16 |
| hsa-miR-186-5p | RP11-488L18.10 | hsa-miR-186-5p | KB-1980E6.3 | hsa-miR-140-3p | AC015849.16 |
| hsa-miR-146b-5p | LINC00861 | hsa-miR-374b-5p | KB-1980E6.3 | hsa-miR-186-5p | RP11-578F21.6 |
| hsa-miR-143-3p | LINC00861 | hsa-miR-140-3p | RP11-370A5.1 | hsa-miR-374b-5p | RP11-578F21.6 |
| hsa-miR-186-5p | LINC00861 | hsa-miR-143-3p | RP1-144F13.3 | hsa-miR-34a-5p | UBE2Q1-AS1 |
| hsa-miR-374b-5p | LINC00861 | hsa-miR-186-5p | AP000654.4 | hsa-miR-148a-3p | UBE2Q1-AS1 |
| hsa-miR-25-3p | LINC00861 | hsa-miR-27b-3p | LINC01267 | hsa-miR-27b-3p | LINC00475 |
| hsa-miR-143-3p | RP11-10C24.1 | hsa-miR-143-3p | RP11-356M20.3 | hsa-miR-125b-5p | LINC00475 |
| hsa-miR-125b-5p | LL22NC03-102D1.18 | hsa-miR-143-3p | RP11-351M8.1 | hsa-miR-140-3p | LINC00475 |

| | | | | | |
|---|---|---|---|---|---|
| hsa-miR-186-5p | RP11-43F13.3 | hsa-miR-125b-5p | RP11-351M8.1 | hsa-miR-374b-5p | LINC00475 |
| hsa-miR-148a-3p | AC007182.6 | hsa-miR-186-5p | LINC00271 | hsa-miR-421 | LINC00475 |
| hsa-miR-148b-3p | AC007182.6 | hsa-miR-27b-3p | RP11-809O17.1 | hsa-miR-34a-5p | LINC00475 |
| hsa-miR-374b-5p | RP11-243A14.1 | hsa-miR-125b-5p | RP11-809O17.1 | hsa-miR-27b-3p | LARS2-AS1 |
| hsa-miR-421 | RP11-173A16.2 | hsa-miR-125b-5p | RP11-503C24.6 | hsa-miR-140-3p | RASGRF2-AS1 |
| hsa-miR-140-3p | RP11-146I2.1 | hsa-miR-26b-5p | RP11-408O19.5 | hsa-miR-143-3p | RP11-508N12.2 |
| hsa-miR-148a-3p | CTC-265F19.3 | hsa-miR-143-3p | LINC01013 | hsa-miR-125b-5p | RP11-508N12.2 |
| hsa-miR-186-5p | CRYM-AS1 | hsa-miR-27b-3p | LINC01013 | hsa-miR-143-3p | AC107081.5 |
| hsa-miR-27b-3p | CRYM-AS1 | hsa-miR-143-3p | AC005537.2 | hsa-miR-25-3p | LINC00563 |
| hsa-miR-140-3p | RP11-474P2.6 | hsa-miR-374b-5p | AC005537.2 | hsa-miR-378h | CTB-91J4.1 |
| hsa-miR-26b-5p | RP11-474P2.6 | hsa-miR-374b-5p | AC010091.1 | hsa-miR-378a-3p | CTB-91J4.1 |
| hsa-miR-378a-3p | RP11-474P2.6 | hsa-miR-143-3p | RP11-108M9.3 | hsa-miR-186-5p | CTA-292E10.9 |
| hsa-miR-378h | RP11-474P2.6 | hsa-miR-125b-5p | RP11-108M9.3 | hsa-miR-146b-5p | CTA-292E10.9 |
| hsa-miR-374b-5p | RP11-474P2.6 | hsa-miR-143-3p | CTD-2024I7.13 | hsa-miR-374b-5p | CTA-292E10.9 |
| hsa-miR-186-5p | RP11-474P2.6 | hsa-miR-374b-5p | CTD-2024I7.13 | hsa-miR-378h | CTA-292E10.9 |
| hsa-miR-34a-5p | RP11-6I2.3 | hsa-miR-499a-5p | RP11-344N10.5 | hsa-miR-378a-3p | CTA-292E10.9 |
| hsa-miR-421 | RP11-6I2.3 | hsa-miR-186-5p | RP11-344N10.5 | hsa-miR-26b-5p | CTA-292E10.9 |
| hsa-miR-140-3p | RP11-148B6.1 | hsa-miR-374b-5p | TRPC5OS | hsa-miR-499a-5p | CTA-292E10.9 |
| hsa-miR-27b-3p | RP11-473M20.9 | hsa-miR-34a-5p | RP11-456H18.2 | hsa-miR-143-3p | CTA-292E10.9 |
| hsa-miR-125b-5p | RP5-1184F4.5 | hsa-miR-143-3p | LINC00856 | hsa-miR-25-3p | CTA-292E10.9 |
| hsa-miR-26b-5p | RP5-1184F4.5 | hsa-miR-378a-3p | LINC00856 | hsa-miR-221-3p | CTA-292E10.9 |
| hsa-miR-499a-5p | CTD-2341M24.1 | hsa-miR-26b-5p | AC005082.12 | hsa-miR-199a-5p | CTA-292E10.9 |
| hsa-miR-143-3p | LINC01206 | hsa-miR-199a-5p | AP002954.4 | hsa-miR-140-3p | CTA-292E10.9 |
| hsa-miR-140-3p | LINC01206 | hsa-miR-148b-3p | AP002954.4 | hsa-miR-499a-5p | ADAM20P1 |
| hsa-miR-27b-3p | LINC01206 | hsa-miR-378a-3p | TPTEP1 | hsa-miR-378a-3p | RP5-1028K7.2 |
| hsa-miR-146b-5p | LINC01206 | hsa-miR-378h | TPTEP1 | hsa-miR-378h | RP5-1028K7.2 |
| hsa-miR-186-5p | LINC01206 | hsa-miR-199a-5p | TPTEP1 | hsa-miR-186-5p | RP11-445L13__B.3 |

| | | | | | |
|---|---|---|---|---|---|
| hsa-miR-199a-5p | LINC01206 | hsa-miR-143-3p | TPTEP1 | hsa-miR-125b-5p | RP11-356J5.13 |
| hsa-miR-186-5p | RP11-230F18.5 | hsa-miR-374b-5p | TPTEP1 | hsa-miR-34a-5p | RP11-356J5.13 |
| hsa-miR-125b-5p | ZNF436-AS1 | hsa-miR-146b-5p | TPTEP1 | hsa-miR-27b-3p | RP11-90B9.3 |
| hsa-miR-199a-5p | ZNF436-AS1 | hsa-miR-421 | TPTEP1 | hsa-miR-140-3p | AC018890.6 |
| hsa-miR-186-5p | RP11-1078H9.5 | hsa-miR-27b-3p | SEMA6A-AS1 | hsa-miR-143-3p | RP11-57H14.4 |
| hsa-miR-125b-5p | CTA-246H3.12 | hsa-miR-148a-3p | SEMA6A-AS1 | hsa-miR-146b-5p | RP11-145P16.3 |
| hsa-miR-140-3p | CTA-246H3.12 | hsa-miR-146b-5p | DARS-AS1 | hsa-miR-125b-5p | RP11-758H9.2 |
| hsa-miR-421 | CTA-246H3.12 | hsa-miR-25-3p | DARS-AS1 | hsa-miR-143-3p | RP11-863P13.3 |
| hsa-miR-34a-5p | RP11-476D10.1 | hsa-miR-140-3p | DARS-AS1 | hsa-miR-26b-5p | KCNMA1-AS1 |
| hsa-miR-27b-3p | ZBTB20-AS1 | hsa-miR-186-5p | DARS-AS1 | hsa-miR-34a-5p | KCNMA1-AS1 |
| hsa-miR-140-3p | RP11-422J15.1 | hsa-miR-374b-5p | DARS-AS1 | hsa-miR-186-5p | RP11-1C8.5 |
| hsa-miR-186-5p | RP11-1060G2.2 | hsa-miR-143-3p | DARS-AS1 | hsa-miR-143-3p | RP11-1C8.5 |
| hsa-miR-26b-5p | DOCK4-AS1 | hsa-miR-27b-3p | RP11-103B5.4 | hsa-miR-148b-3p | RP11-274B21.9 |
| hsa-miR-374b-5p | DOCK4-AS1 | hsa-miR-140-3p | RP1-90G24.11 | hsa-miR-148a-3p | RP11-274B21.9 |
| hsa-miR-34a-5p | LINC00384 | hsa-miR-378a-3p | AC005281.2 | hsa-miR-374b-5p | RP11-274B21.9 |
| hsa-miR-378h | LINC00384 | hsa-miR-378h | AC005281.2 | hsa-miR-146b-5p | RP1-134E15.3 |
| hsa-miR-25-3p | LINC00384 | hsa-miR-499a-5p | AF131215.4 | hsa-miR-125b-5p | RP11-700H6.2 |
| hsa-miR-378a-3p | LINC00384 | hsa-miR-186-5p | AF131215.4 | hsa-miR-140-3p | USP46-AS1 |
| hsa-miR-374b-5p | AC005550.3 | hsa-miR-221-3p | AF131215.4 | hsa-miR-27b-3p | CTC-360G5.9 |
| hsa-miR-143-3p | AC090587.2 | hsa-miR-421 | FGD5-AS1 | hsa-miR-125b-5p | VWA8-AS1 |
| hsa-miR-421 | RP3-461P17.10 | hsa-miR-421 | RP11-305O6.3 | hsa-miR-186-5p | RP11-16C1.2 |
| hsa-miR-140-3p | RP11-65J3.15 | hsa-miR-146b-5p | RP11-70D24.3 | hsa-miR-186-5p | RP1-265C24.8 |
| hsa-miR-143-3p | RP1-90G24.6 | hsa-miR-27b-3p | RP11-886P16.6 | hsa-miR-374b-5p | RP1-265C24.8 |
| hsa-miR-186-5p | RP11-235G24.1 | hsa-miR-499a-5p | RP5-855D21.2 | hsa-miR-143-3p | AC022819.3 |
| hsa-miR-143-3p | RP11-809C18.3 | hsa-miR-143-3p | CTD-3051D23.4 | hsa-miR-143-3p | VIPR1-AS1 |
| hsa-miR-27b-3p | RP11-406H23.2 | hsa-miR-34a-5p | RP11-359G22.2 | hsa-miR-125b-5p | RP5-1142A6.8 |
| hsa-miR-378a-3p | RP1-213J1P__B.1 | hsa-miR-140-3p | RP11-186N15.3 | hsa-miR-143-3p | RP11-138I17.1 |
| hsa-miR-378h | RP1-213J1P__B.1 | hsa-miR-421 | RP11-376M2.2 | hsa-miR-34a-5p | LINC00272 |

| | | | |
|---|---|---|---|
| hsa-miR-421 | LINC00645 | hsa-miR-378h | ANKRD10-IT1 |

**Table S6.** Core genes obtained by cytoHubba's five methods.

| Name | BC | Clo | Degree | EPC | RAD |
|---|---|---|---|---|---|
| *KLC2_16990_AP* | 809.31 | 57.25 | 2 | 40.86 | 4.17 |
| *KLC2_16992_RI* | 809.31 | 57.25 | 2 | 38.94 | 4.17 |
| *ABAT_33905_AA* | 3345.61 | 58.25 | 2 | 39.10 | 4.23 |
| *hsa-miR-125b-5p* | 13604.63 | 85.67 | 55 | 55.98 | 4.83 |
| *hsa-miR-148a-3p* | 5927.85 | 63.68 | 27 | 51.18 | 4.12 |
| *hsa-miR-25-3p* | 5095.70 | 61.67 | 19 | 47.40 | 4.34 |
| *hsa-miR-499a-5p* | 6582.81 | 64.35 | 28 | 43.62 | 4.13 |
| *RP6-159A1.4* | 809.31 | 57.25 | 2 | 41.15 | 4.17 |
| *LL0XNC01-7P3.1* | 809.31 | 57.25 | 2 | 41.01 | 4.17 |
| *CTD-3162L10.1* | 1594.77 | 56 | 2 | 40.88 | 4.10 |
| *AC004448.5* | 2042.34 | 57.5 | 2 | 38.05 | 4.19 |
| TOP 15 | 809.31 | 50.83 | 2 | 36.761 | 3.84 |

**Table S7.** Correlation between *ABAT* methylation probes and *ABAT_33905_AP* expression in KIRC.

| Methylation probe | Cor | P-value |
|---|---|---|
| cg02564313 | -0.07523 | 0.165739 |
| cg16586594 | 0.061454 | 0.25775 |
| cg01221157 | 0.02398 | 0.659032 |
| cg07439546 | -0.04176 | 0.442113 |
| cg00220102 | 0.062111 | 0.25269 |
| cg16704802 | -0.10655 | 0.049294 |
| cg22217807 | -0.12534 | 0.020605 |
| cg03389720 | 0.094953 | 0.079956 |
| cg08241330 | 0.063134 | 0.244938 |
| cg07740911 | -0.01757 | 0.746525 |
| cg04332619 | 0.045426 | 0.403043 |
| cg05372963 | 0.018267 | 0.736787 |
| cg09489090 | -0.00927 | 0.864564 |
| cg10292855 | 0.028503 | 0.599919 |
| cg26915924 | 0.044824 | 0.40931 |
| cg07431888 | -0.01913 | 0.724844 |
| cg07336964 | -0.04526 | 0.404789 |
| cg26662674 | 0.054508 | 0.315567 |
| cg20587543 | -0.07961 | 0.142366 |
| cg08877512 | 0.058399 | 0.282207 |
| cg03441945 | 0.076003 | 0.161405 |
| cg01881182 | 0.034771 | 0.522226 |
| cg06604249 | -0.06997 | 0.19743 |
| cg05007180 | -0.04022 | 0.459183 |
| cg03698956 | -0.03438 | 0.526938 |
| cg03500056 | 0.022528 | 0.678488 |

| | | |
|---|---|---|
| cg08834902 | 0.067387 | 0.214525 |
| cg00231389 | 0.062271 | 0.251467 |
| cg26778345 | 0.073246 | 0.177199 |
| cg03142071 | -0.08381 | 0.122416 |
| cg03718620 | -0.05242 | 0.334511 |
| cg15890111 | 0.016985 | 0.754642 |
